# Effects of α-Cyclodextrin on Cholesterol Control and Hydrolyzed Ginseng Extract on Glycemic Control in People With Prediabetes: A Randomized Clinical Trial

**DOI:** 10.1001/jamanetworkopen.2020.23491

**Published:** 2020-11-17

**Authors:** Erica Bessell, Nicholas R. Fuller, Tania P. Markovic, Namson S. Lau, Jessica Burk, Chelsea Hendy, Tegan Picone, Ang Li, Ian D. Caterson

**Affiliations:** 1 The Boden Collaboration, Charles Perkins Centre, The University of Sydney, Sydney, Australia; 2 Metabolism and Obesity Services, Royal Prince Alfred Hospital, Camperdown, Australia; 3 Sydney Health Economics, Sydney Local Health District, Camperdown, Australia

## Abstract

**Importance:**

Effective strategies for preventing type 2 diabetes are needed. Many people turn to complementary medicines, but there is little well-conducted scientific evidence to support their use.

**Objective:**

To assess the efficacy of α-cyclodextrin for cholesterol control and that of hydrolyzed ginseng for glycemic control in people with prediabetes and overweight or obesity.

**Design, Setting, and Participants:**

This 6-month double-blind, placebo-controlled, randomized clinical trial, with a 2 × 2 factorial design, was conducted between July 2015 and October 2018 at 2 locations in Sydney, Australia. Eligible participants were aged 18 years or older, had a body mass index (weight in kilograms divided by height in meters squared) of 25 or higher, and had prediabetes within 6 months of study entry according to the American Diabetes Association guidelines. Data analysis was performed from May to August 2019.

**Interventions:**

Participants were randomized to 1 of 4 groups to take active or placebo versions of each supplement (α-cyclodextrin plus hydrolyzed ginseng, α-cyclodextrin plus placebo, placebo plus hydrolyzed ginseng, or placebo plus placebo) for 6 months. All participants received dietetic advice for weight loss.

**Main Outcomes and Measures:**

The primary outcomes were the differences in total cholesterol and fasting plasma glucose between groups after 6 months. The primary analysis used the intention-to-treat principle. Multiple predetermined subsample analyses were conducted.

**Results:**

A total of 401 participants were eligible for the study (248 women [62%]; mean [SD] age, 53.5 [10.2] years; mean [SD] body mass index, 34.6 [6.2]). One hundred one patients were randomized to receive α-cyclodextrin plus hydrolyzed ginseng, 99 were randomized to receive α-cyclodextrin plus placebo, 101 were randomized to receive placebo plus hydrolyzed ginseng, and 100 were randomized to receive placebo plus placebo. For 200 participants taking α-cyclodextrin compared with 201 participants taking placebo, there was no difference in total cholesterol after 6 months (−1.5 mg/dL; 95% CI, −6.6 to 3.5 mg/dL; *P* = .51). For 202 participants taking hydrolyzed ginseng compared with 199 participants taking placebo, there was no difference in fasting plasma glucose after 6 months (0.0 mg/dL; 95% CI, −1.6 to 1.8 mg/dL; *P* = .95). Use of α-cyclodextrin was associated with constipation (16 participants vs 4 participants; *P* = .006) and cough (8 participants vs 1 participant; *P* = .02). Use of hydrolyzed ginseng was associated with rash and pruritus (13 participants vs 2 participants; *P* = .006). Only 37 of 401 participants (9.2%) experienced these adverse events.

**Conclusions and Relevance:**

Although they are safe for use, there was no benefit found for either α-cyclodextrin for cholesterol control or hydrolyzed ginseng for glycemic control in people with prediabetes and overweight or obesity.

**Trial Registration:**

Australian New Zealand Clinical Trials Registry Identifier: ACTRN12614001302640

## Key Points

**Question:**

Can α-cyclodextrin and hydrolyzed ginseng aid in cholesterol and glycemic control, respectively, in people with prediabetes and overweight or obesity?

**Findings:**

In this double-blind, placebo-controlled, randomized clinical trial of 401 participants, there was no significant difference in total cholesterol in those taking α-cyclodextrin or in fasting plasma glucose in those taking hydrolyzed ginseng after 6 months, compared with placebo.

**Meaning:**

There was no clinically relevant beneficial effect of α-cyclodextrin on cholesterol control or hydrolyzed ginseng on glycemic control in people with prediabetes and overweight or obesity.

## Teaser

This randomized clinical trial assesses the efficacy of α-cyclodextrin for cholesterol control and that of hydrolyzed ginseng for glycemic control in people with prediabetes and overweight or obesity.

## Introduction

The number of people with type 2 diabetes has been steadily increasing and is predicted to continue to increase.^1^ In Australia, the prevalence tripled from 1990 to 2014, and a further 2 million Australians are estimated to have prediabetes^2^ (impaired fasting plasma glucose [FPG] and/or impaired glucose tolerance). Obesity is often associated with either prediabetes or type 2 diabetes, and with 31% of the Australian adult population having obesity (and a further 36% overweight),^3^ interventions to reduce weight and improve health are a priority.

Complementary medicines are now a $5 billion industry in Australia,^4^ with people using these alternative treatments not only for preventive health but to manage chronic disease. However, quality evidence is lacking regarding the efficacy and safety of many complementary medicines used in the treatment of obesity and type 2 diabetes. Two such medicines are α-cyclodextrin and ginseng.^5^

α-Cyclodextrin, a soluble fiber derived from corn starch, has a unique structure allowing it to bind more triglycerides than most fibers,^6^ up to 9 times its weight.^7^ A few small, short-term clinical trials^8,9^ have shown promising results for weight loss, weight maintenance, and cholesterol-lowering in people with overweight and obesity.

Ginseng’s effects on glycemic control have recently been studied.^5,10,11^ The ginsenoside components of ginseng,^12,13^ especially compound K, a final metabolite of protopanaxadiol ginsenosides by intestinal bacteria,^14,15^ are believed to provide the beneficial glycemic effect. Compound K is created synthetically by hydrolyzing ginseng in the laboratory. Hydrolyzed ginseng has been tested in a few small, short-term clinical trials^16,17^ and was found to improve fasting and postprandial glucose levels in people with prediabetes and type 2 diabetes.

This double-blind, placebo-controlled, randomized clinical trial was designed to produce more robust evidence for the use of 2 complementary medicines using the same marketed products tested in previous trials.^8,9,16^ To our knowledge, it is the largest and longest clinical trial investigating the effects of these 2 medicines and the first to investigate either product in people with prediabetes and overweight or obesity. The primary objectives of this trial were to determine the efficacy of α-cyclodextrin for cholesterol control and the efficacy of hydrolyzed Korean ginseng (*Panax ginseng*), which is rich in compound K, for glycemic control.

## Methods

### Study Design and Participants

This double-blind, randomized clinical trial was conducted between July 2015 and October 2018 at the Royal Prince Alfred and Nepean Hospitals, Australia. The trial was investigator initiated and designed and conducted independently of SFI Research Pty Ltd, which provided the investigational and placebo products and funding to support the conduct of the trial. The Human Research Ethics Committees at Sydney Local Health District and the University of Sydney approved this trial. After being provided information about the trial, participants signed and dated a consent form before any trial-specific procedures were conducted. This study follows Consolidated Standards of Reporting Trials (CONSORT) reporting guideline.

To be eligible, participants had to be aged 18 years or older, provide evidence of prediabetes within 6 months of study entry, and have a body mass index (BMI; weight in kilograms divided by height in meters squared) of 25 or higher. Prediabetes was defined according to the American Diabetes Association guidelines,^18^ including FPG of 100 to 125 mg/dL (to convert glucose to millimoles per liter, multiply by 0.0555), 2-hour postchallenge (oral glucose tolerance test) plasma glucose of 140 to 199 mg/dL, or glycated hemoglobin (HbA_1c_) of 5.7% to 6.4% (to convert to proportion of total hemoglobin, multiply by 0.01). The trial protocol, including a full list of the eligibility criteria, has been published previously (Supplement 1).^19^

### Randomization and Masking

Eligible participants who consented to participate were randomized to 1 of 4 groups in a 1:1:1:1 ratio, using simple block randomization with a computer-generated randomization program. Group allocation was completed by an investigator after eligibility was confirmed and before the participant’s baseline visit. Each participant received either 2 active products (α-cyclodextrin [αCD] plus hydrolyzed ginseng extract [HGE]), 1 active product and 1 placebo product (αCD plus placebo or placebo plus HGE), or 2 placebo products (placebo plus placebo). Participants and investigators were blinded as to which group the participant had been allocated. All containers of the investigational product or placebo were packaged identically, apart from the information of blinded group allocation (ie, group A, B, C, or D).

### Procedures

Participants attended monthly visits during the 6-month intervention and were asked to take 12 pills daily (2 capsules before and 2 tablets after breakfast, lunch, and dinner). Each capsule contained either 160 mg of HGE or placebo (maltodextrin), and each tablet contained either 1000 mg of αCD or placebo (microcrystalline cellulose). The placebo pills were indistinguishable from their active counterparts. Participants were provided a 1-month supply of pills at each visit, and leftover pills were returned at subsequent visits to monitor compliance. Participants met with a study dietitian monthly and received personalized advice for prevention of type 2 diabetes, focusing on a healthy, hypocaloric diet, moderate intensity exercise, and behavior management.^19^

### Outcomes

Anthropometric, medical, and behavioral outcome measures were collected before, during, and after the intervention; details have been published previously.^19^ The primary outcomes of total cholesterol and FPG and the secondary outcomes of weight, HbA_1c_, low-density lipoprotein (LDL) cholesterol, and triglycerides were measured at the screening or baseline visit, and at the 6-month visit (end of intervention period). Safety measurements assessed over the course of the trial included routine pathology tests and clinical vital signs.^19^ Adverse events were monitored at every visit.^19^

### Statistical Analysis

The primary objectives of this trial were to determine the efficacy of αCD for lipid control and that of HGE for glycemic control—namely, the difference in total cholesterol and FPG between experiment and placebo groups after 6 months of treatment. An estimated 200 participants were required to provide 80% power to detect a mean reduction of 8.5 mg/dL in FPG in those taking HGE compared with placebo, at a 2-sided significance level of 2.5% and allowing for a dropout rate of 15% at 6 months. Under the same conditions, 143 participants were required to detect a mean reduction of 19 mg/dL (to convert to millimoles per liter, multiply by 0.0259) in fasting total cholesterol in those taking αCD compared with placebo. As such, 401 participants were recruited and randomized to 1 of 4 groups.

Data analysis was performed using SPSS statistical software version 22 (IBM) and Excel software version 16 (Microsoft). For the efficacy variables, an intention-to-treat (ITT) analysis was conducted, with all participants who were randomized and attended the baseline visit included. Multiple imputation by predictive mean matching (*k* = 1) using demographic and clinical characteristics as covariates was used to impute missing values from the screening, baseline, 3-month, and 6-month visits. Thirty imputed data sets were created and amalgamated for the analyses listed later. Data analysis was performed from May to August 2019.

The 2 × 2 factorial design of this trial allowed for 2 groups to be combined to assess the effect of αCD vs placebo and HGE vs placebo, as well as the combined effect of αCD and HGE vs placebo. These comparisons were conducted using an analysis of covariance with adjustment for the initial observation. Predetermined subsample analyses were conducted to test the observed effects in participants who completed the intervention (attended the 6-month visit), were compliant with the supplements (consumed at least 80% of the required pills for at least 50% of the time they were in the trial), met the guidelines for prediabetes at their screening visit, and had elevated cholesterol at screening (total cholesterol >212 mg/dL).

The χ^2^ test or Fisher exact test, when necessary, was used to compare the number of participants experiencing adverse events of interest across participants on HGE, αCD, and placebo. Pathology and clinical measurements collected at 6 months were analyzed to detect differences between αCD vs placebo and HGE vs placebo using analysis of covariance, with adjustment for the initial observation. For each safety measure, these analyses only included participants with no missing data.

For all statistical tests, 2-sided *P* < .05 was considered statistically significant. Descriptive values are presented as a mean (SD) unless otherwise indicated. The test results for differences between groups are presented as estimated differences with 95% CIs and *P* values.

## Results

### Participants

The Figure depicts the participant flow. Between July 2015 and March 2018, 401 participants (248 women [62%]; mean [SD] age, 53.5 [10.2] years; mean [SD] BMI, 34.6 [6.2]) were enrolled in the trial, with the final participants completing in October 2018. One hundred one patients were randomized to receive α-cyclodextrin plus hydrolyzed ginseng, 99 were randomized to receive α-cyclodextrin plus placebo, 101 were randomized to receive placebo plus hydrolyzed ginseng, and 100 were randomized to receive placebo plus placebo. The dropout rate was 17% (68 participants) at the end of the 6-month intervention period. Fifteen noncompleters received α-cyclodextrin plus hydrolyzed ginseng, 16 received HGE only, 26 received αCD only, and 11 received double placebo. The difference in the number of noncompleters among the 4 groups was significant (*P* = .03), but no reason was identified (Figure).

**Figure.**
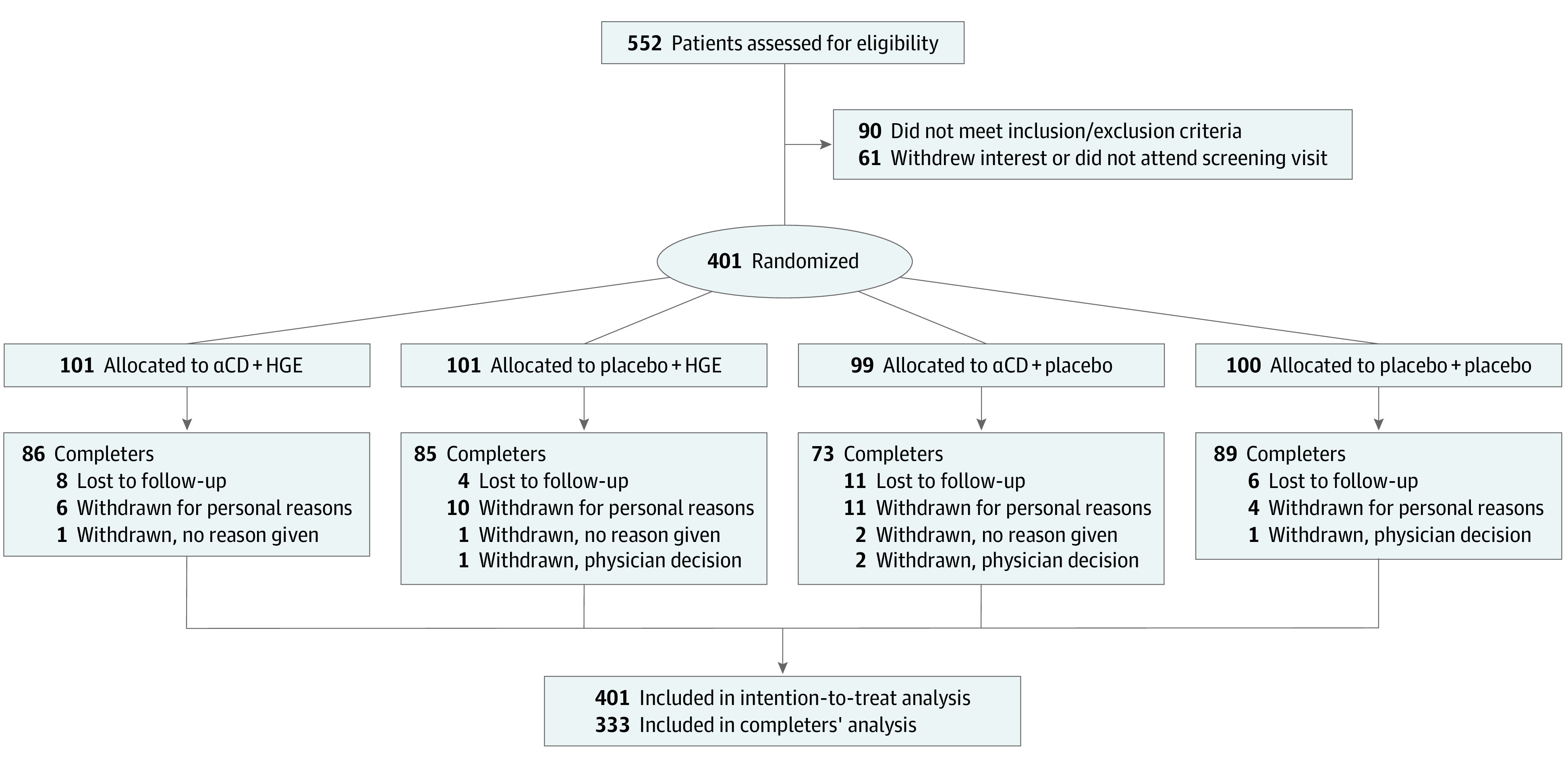
Participant Flow Through Screening, Randomization, Follow-up, and Analysis αCD indicates α-cyclodextrin; and HGE, hydrolyzed ginseng extract.

Baseline demographic and clinical characteristics were well matched across the 4 groups (Table 1). Although all participants had external results meeting the diagnosis of prediabetes within 6 months of screening,^19^ only 226 participants (56%) met the guidelines for prediabetes on the basis of blood samples taken at their screening visit. FPG ranged from 74 to 141 mg/dL (mean [SD], 97 [11] mg/dL), and HbA_1c_ ranged from 4.5% to 6.7%. Among patients who met the guidelines for prediabetes, the mean (SD) FPG was 105 (9) mg/dL. The number of participants who met each guideline for prediabetes at their screening visit are shown in Table 2.

**Table 1.** Baseline Characteristics of the Participants

Characteristic	Mean (SD)				
	HGE + αCD (n = 101)	HGE only (n = 101)	αCD only (n = 99)	Double placebo (n = 100)	Overall (N = 401)
Site, participants, No.					
Royal Prince Alfred	83	89	85	78	335
Nepean	18	12	14	22	66
Female, participants, No. (%)	66 (65)	62 (61)	61 (62)	59 (59)	248 (62)
Age, y	52.8 (10.7)	54.0 (9.3)	53.6 (9.7)	53.7 (11.1)	53.5 (10.2)
Weight, kg	97.6 (19.3)	99.4 (22.5)	96.1 (20.0)	99.5 (18.1)	98.2 (20.0)
Body mass index^a^	34.3 (5.8)	35.1 (7.1)	34.2 (6.4)	35.0 (5.5)	34.6 (6.2)
Prediabetes, participants, No. (%)^b^	52 (52)	57 (56)	54 (55)	63 (63)	226 (56)
Fasting plasma glucose, mg/dL	97 (11)	97 (11)	99 (11)	97 (9)	97 (11)
HbA_1c_, %	5.5 (0.3)	5.6 (0.3)	5.6 (0.4)	5.6 (0.3)	5.6 (0.3)
Cholesterol, mg/dL					
Total	216 (39)	224 (39)	216 (39)	224 (46)	220 (39)
High total, participants, No. (%)^c^	53 (52)	65 (64)	56 (57)	54 (54)	228 (57)
LDL	139 (31)	143 (35)	139 (31)	143 (35)	139 (35)
HDL	54 (12)	54 (12)	54 (12)	54 (12)	54 (12)
Triglycerides	124 (53)	150 (71)	133 (71)	168 (310)	142 (159)

Abbreviations: αCD, α-cyclodextrin; HbA_1c_, glycated hemoglobin; HDL, high-density lipoprotein; HGE, hydrolyzed ginseng extract; LDL, low-density lipoprotein.

SI conversion factors: To convert fasting glucose to mmol/L, multiply by 0.0555; total, LDL and HDL cholesterol to mmol/L, multiply by 0.0259; and triglycerides to mmol/L, multiply by 0.0113.

^a^ Body mass index is calculated as weight in kilograms divided by height in meters squared.

^b^ Denotes proportion of participants who met the guidelines for prediabetes at their screening visit.

^c^ Denotes proportion of participants with total cholesterol greater than 212 mg/dL at their screening visit.

**Table 2.** Participants Who Met the Guidelines for Prediabetes at Screening for Fasting Plasma Glucose or HbA_1c_ Levels^a^

Criteria for prediabetes	Participants, No. (%)	Mean (SD)
Fasting plasma glucose at screening, mg/dL		
<100 (healthy range)	234 (58)	90 (5)
100-125 (range for prediabetes)	162 (40)	106 (5)
≥126 (range for type 2 diabetes)	5 (1)	132 (5)
HbA_1c_ at screening, %		
<5.7 (healthy range)	246 (61)	5.4 (0.2)
5.7-6.4 (range for prediabetes)	152 (38)	5.9 (0.2)
≥6.5 (range for type 2 diabetes)	3 (1)	6.6 (0.1)
Participants who met either guideline for prediabetes	226 (56)	
Fasting plasma glucose, mg/dL		105 (9)
HbA_1c_, %		5.7 (0.3)

Abbreviation: HbA_1c_, glycated hemoglobin.

SI conversion factors: To convert fasting glucose to mmol/L, multiply by 0.0555; HbA_1c_ to proportion of total hemoglobin, multiply by 0.01.

^a^ Guidelines for prediabetes were taken from the American Diabetes Association.^18^

Only 164 participants (41%) were considered compliant to taking both investigational products, ranging from 36% to 49% of participants compliant across the 4 groups; the difference among groups was not significant. Of these compliant participants, 90% completed the 6-month intervention period. In terms of each investigational product, 176 participants (44%) were considered compliant with the αCD or placebo regimen, and 195 participants (49%) were considered compliant with the HGE or placebo regimen.

### Cholesterol Control

There was no significant difference in total cholesterol from screening to 6 months between participants taking αCD or placebo (−1.5 mg/dL; 95% CI, −6.6 to 3.5 mg/dL; *P* = .51) (Table 3). No significant effect of αCD on total cholesterol was observed in 333 participants who completed the intervention period (−3.5 mg/dL; 95% CI, −8.9 to 2.3 mg/dL; *P* = .23) (eTable 1 in Supplement 2), 176 participants who were compliant with the αCD or placebo regimen (1.2 mg/dL; 95% CI, −7.0 to 9.3 mg/dL; *P* = .80) (eTable 2 in Supplement 2), or the 228 participants who had high cholesterol at their screening visit (0.8 mg/dL; 95% CI, −6.6 to 8.1 mg/dL; *P* = .82) (eTable 3 in Supplement 2). These subsample analyses were adequately powered (with ≥143 participants) to detect a difference according to the power calculations. Among participants who completed the intervention period, there was a slightly greater reduction in LDL cholesterol in those taking αCD compared with placebo at 6 months but the difference was not significant (−5.0 mg/dL; 95% CI, −9.7 to 0.0 mg/dL; *P* = .05) (eTable 1 in Supplement 2). This finding was not seen in the ITT analysis (Table 3), nor was it replicated in other subsample analyses. No significant interactions were observed between αCD and HGE for total cholesterol in the ITT or completers analysis.

**Table 3.** Comparison of Outcomes at 6 Months Between Participants Taking αCD and Participants Taking Placebo

Characteristic	Mean (SD)				Adjusted difference (95% CI)^a^	*P* value
	αCD (n = 200)		Placebo (n = 201)			
	Baseline	Month 6	Baseline	Month 6		
Weight, kg	96.9 (19.7)	93.6 (19.4)	99.4 (20.4)	95.4 (19.6)	0.64 (−0.23 to 1.52)	.15
Body mass index^b^	34.2 (6.1)	33.0 (6.0)	35.0 (6.4)	33.7 (6.3)	0.12 (−0.19 to 0.42)	.45
Weight loss, %	NA	3.4 (4.2)	NA	4.0 (4.3)	−0.52 (−1.35 to 0.32)	.22
Fasting plasma glucose, mg/dL	97 (11)	96 (11)	97 (9)	96 (11)	0.7 (−1.1 to 2.3)	.41
HbA_1c_, %	5.6 (0.4)	5.5 (0.4)	5.6 (0.3)	5.5 (0.3)	0.02 (−0.02 to 0.06)	.35
Cholesterol, mg/dL						
Total	216 (39)	216 (35)	224 (42)	220 (35)	−1.5 (−6.6 to 3.5)	.51
LDL	139 (31)	135 (31)	143 (35)	139 (31)	−3.5 (−7.7 to 1.2)	.13
HDL	54 (12)	54 (12)	54 (12)	54 (12)	0.4 (−0.8 to 1.9)	.40
Triglycerides	124 (62)	133 (80)	159 (221)	142 (80)	0.9 (−11.5 to 13.3)	.92

Abbreviations: αCD, α-cyclodextrin; HbA_1c_, glycated hemoglobin; HDL, high-density lipoprotein; LDL, low-density lipoprotein; NA, not applicable.

SI conversion factors: To convert fasting plasma glucose to mmol/L, multiply by 0.0555; HbA_1c_ to proportion of total hemoglobin, multiply by 0.01; total, LDL, and HDL cholesterol to mmol/L, multiply by 0.0259; and triglycerides to mmol/L, multiply by 0.0113.

^a^ Adjusted for baseline observation.

^b^ Body mass index is calculated as weight in kilograms divided by height in meters squared.

### Glycemic Control

There were no significant differences in FPG (0.0 mg/dL; 95% CI, −1.6 to 1.8 mg/dL; *P* = .95) or HbA_1c_ (−0.02%; 95% CI, −0.06% to 0.01%; *P* = .22) in participants taking HGE compared with placebo in the ITT (Table 4) or the completers analysis of 333 participants (FPG, 0.2 mg/dL; 95% CI, −1.8 to 2.2 mg/dL; *P* = .86; HbA_1c_, −0.02%; 95% CI, −0.07 to 0.02%; *P* = .36) (eTable 4 in Supplement 2), in 195 participants who were compliant with the HGE or placebo regimen (FPG, 1.1 mg/dL; 95% CI, −1.3 to 3.4 mg/dL; *P* = .39; HbA_1c_, −0.02%; 95% CI, −0.07% to 0.04%; *P* = .53) (eTable 5 in Supplement 2), or 226 participants who met the guidelines for prediabetes at screening (FPG, 0.0 mg/dL; 95% CI, −2.7 to 2.7 mg/dL; *P* = .99; HbA_1c_, −0.01%; 95% CI, −0.07% to 0.05%; *P* = .76) (eTable 6 in Supplement 2). The subsample of participants who met the guidelines for prediabetes at screening was of adequate size (with ≥200 participants) to detect a difference according to the power calculations. There were no significant interactions between αCD and HGE for FPG or HbA_1c_ in the ITT or completers analysis.

**Table 4.** Comparison of Outcomes at 6 Months Between Participants Taking HGE and Participants Taking Placebo

Characteristic	Mean (SD)				Adjusted difference (95% CI)^a^	*P* value
	HGE (n = 202)		Placebo (n = 199)			
	Baseline	Month 6	Baseline	Month 6		
Weight, kg	98.5 (21.0)	94.5 (20.3)	97.8 (19.1)	94.5 (18.7)	−0.59 (−1.47 to 0.29)	.19
Body mass index^b^	34.7 (6.5)	33.3 (6.3)	34.6 (6.0)	33.5 (6.0)	−0.32 (−0.63 to −0.02)	.04^c^
Weight loss, %	NA	4.0 (4.5)	NA	3.4 (4.0)	0.63 (−0.20 to 1.47)	.14
Fasting glucose, mg/dL	97 (11)	96 (11)	99 (11)	96 (11)	0.0 (−1.6 to 1.8)	.95
HbA_1c_, %	5.6 (0.3)	5.5 (0.3)	5.6 (0.4)	5.5 (0.4)	−0.02 (−0.06 to 0.01)	.22
Cholesterol, mg/dL						
Total	220 (39)	220 (35)	220 (42)	216 (35)	1.5 (−3.5 to 6.6)	.51
LDL	139 (35)	139 (31)	139 (35)	139 (31)	0.4 (−3.9 to 5.0)	.84
HDL	54 (12)	54 (12)	54 (12)	54 (12)	−0.8 (−1.9 to 0.8)	.33
Triglycerides	133 (62)	133 (80)	150 (221)	133 (80)	7.1 (−6.2 to 19.5)	.29

Abbreviations: HbA_1c_, glycated hemoglobin; HDL, high-density lipoprotein; HGE, hydrolyzed ginseng extract; LDL, low-density lipoprotein; NA, not applicable.

SI conversion factors: To convert fasting plasma glucose to mmol/L, multiply by 0.0555; HbA_1c_ to proportion of total hemoglobin, multiply by 0.01; total, LDL, and HDL cholesterol to mmol/L, multiply by 0.0259; and triglycerides to mmol/L, multiply by 0.0113.

^a^ Adjusted for baseline observation.

^b^ Body mass index is calculated as weight in kilograms divided by height in meters squared.

^c^ Statistically significant difference between groups, after adjustment for baseline in the 2 × 2 factorial model (*P* < .05).

### Change in Weight

Among 165 participants who were compliant with both investigational products, those taking HGE weighed significantly less than participants taking placebo at 6 months (−1.63 kg; 95% CI, −3.03 to −0.22 kg; *P* = .02) (eTable 7 in Supplement 2). These participants had a significantly higher percentage weight loss than participants taking placebo (1.7%; 95% CI, 0.3% to 3.1%; *P* = .02) (eTable 7 in Supplement 2). This effect of HGE on weight was not observed in the ITT (−0.59 kg; 95% CI, −1.47 to 0.29 kg; *P* = .19) (Table 4) or completers analysis (−0.54 kg; 95% CI, −1.45 to 0.38 kg; *P* = .25) (eTable 4 in Supplement 2) but in the ITT analysis, participants taking HGE had a significantly lower BMI than participants taking placebo at 6 months (−0.32; 95% CI, −0.63 to −0.02; *P* = .04) (Table 4). No such associations were observed for participants taking αCD vs placebo. No significant interactions were observed between αCD and HGE for percentage weight loss in the ITT or completers analysis.

### Safety

Both investigational products appeared to be safe for use on the basis of the biochemical and clinical safety measures analyzed after 6 months’ treatment. The number of participants reporting adverse events that were at any stage rated as possibly or probably related to the investigational product are given in eTable 8 in Supplement 2. Two of these adverse events were more commonly reported in participants taking αCD compared with those taking placebo: constipation (13 participants vs 2 participants; *P* = .006) and cough (8 participants vs 1 participant; *P* = .02). Rash or pruritus was reported more often with HGE compared with placebo (13 participants vs 2 participants; *P* = .006). Only 37 of 401 participants (9.2%) were affected by these adverse events. None of the 13 serious adverse events reported was considered to be related to the investigational products.

## Discussion

This randomized clinical trial found no significant effect of αCD on cholesterol control or of HGE on glycemic control in people with prediabetes and overweight or obesity at the end of the 6-month intervention. Both supplements were generally safe, but a small number of participants reported constipation or cough associated with αCD and rash or pruritus associated with HGE.

Although no significant effect of αCD on cholesterol control or weight was seen in the ITT analysis, a slight but nonsignificant reduction was observed for LDL cholesterol in the completers’ analysis, suggesting that αCD may have some metabolic effect even if it is not clinically relevant. The consensus is that products able to lower LDL cholesterol levels by 10% or more are worthwhile for cardiovascular disease risk management, even if long-term efficacy for cardiovascular end points has not been established.^20^ Two previous studies with αCD^8,9^ had shown promising results, but with their small sample sizes (41 and 47 participants), further investigations, as conducted in this trial, were warranted. In the first human trial of αCD,^8^ participants with type 2 diabetes and obesity taking αCD gained 0.3 kg during the 3-month study, whereas those taking placebo gained 1.5 kg, which was a significant increase from baseline. There were reductions in total cholesterol levels in participants taking αCD, but only in those with hypertriglyceridemia at baseline.^8^ In another study^9^ with a 2-month intervention period in overweight but otherwise healthy participants, those taking αCD had lower weight (−0.4 kg), total cholesterol (−12 mg/dL), and LDL cholesterol (−8 mg/dL) compared with participants taking placebo. A slightly larger study^21,22^ of 75 healthy participants with mean BMI 25 and mean total cholesterol of approximately 170 mg/dL at baseline, reported no changes in the lipid profile after 3 months of supplementation with αCD.

HGEs have been studied for their glucose-lowering effects in participants with impaired FPG and type 2 diabetes in only 2 previous short studies with small participants numbers.^16,17^ One 8-week study with 23 participants^16^ reported significantly reduced FPG and postprandial glucose at 60 minutes with HGE supplementation and the other, a 4-week study with 42 participants,^17^ reported a significant reduction in postprandial glucose at 120 minutes postmeal and the glucose area under the curve compared with placebo. In the current trial, glucose tolerance tests were not conducted and HbA_1c_ (which provides an assessment of the average plasma glucose level over the preceding 3 months) was used instead because it is easier to perform and less burdensome for the participant. No significant differences were observed for these measures at 6 months in participants taking HGE compared with placebo. The degree of glucose intolerance in each cohort may explain the difference between the findings of the current trial and the previous studies. The baseline FPG was 108 mg/dL in one study,^16^ and in the other, the FPG in the ginseng group reduced from 117 mg/dL at baseline to 110 mg/dL after 4 weeks.^17^ In the current trial, the mean FPG at baseline was 97 mg/dL because the participants’ eligibility was based on pathology results demonstrating prediabetes from FPG or HbA_1c_ taken within 6 months before their screening visit. However, even among participants who met the guidelines for prediabetes at screening, the mean FPG at baseline was still lower at 105 mg/dL and there was no benefit of HGE supplementation. It is possible that HGE may have an effect in people with greater dysglycemia.

### Strengths and Limitations

To our knowledge, this is the largest and longest clinical trial investigating the effects of these 2 medicines and the first to investigate either product in people with prediabetes and overweight or obesity. The strengths of this research include the large sample size and the use of the reference standard double-blind, placebo-controlled design. The completer analyses help to confirm the results observed in the ITT analysis, and similar results were seen in the subsample analyses.

This study also has limitations that should be acknowledged. Only 44% of participants in this trial were compliant with the αCD or placebo regimen, and 49% of participants were compliant with the HGE or placebo regimen, which may have limited the ability to observe an effect. The large number of tablets or capsules (12 per day) that were required to be taken is likely an important factor. However, even when analyses were conducted using data from the 164 compliant participants, still no significant effects were observed for the primary outcomes. In compliant participants, there was a statistically significant but small effect on weight in those taking HGE compared with placebo after 6 months (−1.63 kg; 95% CI, −3.03 to −0.23 kg; *P* = .02) but this is unlikely to be of any clinical relevance.^23^ The low level of compliance has implications for the real world because 6 tablets of αCD and 6 capsules of HGE would be required for any potential effect. Although 6 pills is less burdensome than 12 pills daily, it is well documented that medication nonadherence is a common issue even when few pills are required daily and nonadherence is especially common in people taking medications to treat chronic conditions, such as type 2 diabetes and hypercholesterolemia.^24^

## Conclusions

This double-blind, placebo-controlled, randomized clinical trial of participants with prediabetes and overweight or obesity showed no significant effect of αCD on cholesterol or of HGE on glycemic control over 6 months. In participants who completed the 6-month intervention, there was a slight reduction in LDL cholesterol among participants taking αCD compared with placebo but the difference was not significant. Although both supplements can be used with safety in otherwise healthy adults with prediabetes and overweight or obesity, these supplements cannot be recommended for diabetes prevention.

## Notes

Supplement 1. **Trial Protocol**

Supplement 2. **eTable 1**. Comparison of Outcomes at Six Months Between Participants Taking Alpha-Cyclodextrin (n = 159) and Participants Taking Placebo (n = 174) in the Completers Analysis **eTable 2.** Comparison of Outcomes at Six Months Between Participants Taking Alpha-Cyclodextrin (n = 86) and Participants Taking Placebo (n = 90) in the Subsample of Participants Who Were Compliant With the Dosing Regimen **eTable 3.** Comparison of Outcomes at Six Months Between Participants Taking Alpha-Cyclodextrin (n = 109) and Participants Taking Placebo (n = 119) in the Subsample of Participants Who Had High Cholesterol at Baseline **eTable 4.** Comparison of Outcomes at Six Months Between Participants Taking Hydrolysed Ginseng Extract (n = 171) and Participants Taking Placebo (n = 162) in the Completers Analysis **eTable 5.** Comparison of Outcomes at Six Months Between Participants Taking Hydrolysed Ginseng Extract (n = 99) and Participants Taking Placebo (n = 96) in the Subsample of Participants Who Were Compliant With the Dosing Regimen **eTable 6.** Comparison of Outcomes at Six Months Between Participants Taking Hydrolysed Ginseng Extract (n = 109) and Participants Taking Placebo (n = 117) in the Subsample of Participants Who Met the Guidelines for Prediabetes at Baseline **eTable 7.** Comparison of Outcomes at Six Months Between Participants Taking Hydrolysed Ginseng Extract (n = 79) and Participants Taking Placebo (n = 85) in the Subsample of Participants Who Were Compliant With the Dosing Regimen for Both Supplements **eTable 8.** Number of Participants Reporting Adverse Events Rated as “Possibly” or “Probably” Related to the Investigational Products Between Baseline and Six Months

Supplement 3. **Data Sharing Statement**
