# Supplementary material for: Effects of α-Cyclodextrin on Cholesterol Control and Hydrolyzed Ginseng Extract on Glycemic Control in People With Prediabetes: A Randomized Clinical Trial
Source: JAMA Netw Open. 2020 Nov 17;3(11):e2023491. doi: 10.1001/jamanetworkopen.2020.23491 (PMC7672512; doi:10.1001/jamanetworkopen.2020.23491)
Supplement: Supplement 1. — Trial Protocol [file jamanetwopen-e2023491-s001.pdf]

double blinded, randomised controlled trial to determine<br>a) the efficacy of FBCx (a formula based on α-cyclodextrin (marketed under the trade name FBCx)) on cholesterol control, and<br>b) the efficacy of GinstT15 (a ginseng extract formula based on Compound K) on glycaemic control	
Version & Date:	Version 7.0: 21 February 2017
Protocol Number	SFI 121
Sponsor	The University of Sydney<br>NSW 2006 Australia<br>Ph: +61 2 9036 2222
Principal Investigators	Professor Ian Caterson/Dr Nicholas Fuller (The University agrees that should Prof Caterson not remain as PI, he will be replaced by another Director of the Institute as co-PI with Dr Fuller)
Principal Investigator address	The Boden Institute<br>Charles Perkins Centre D17<br>The University of Sydney NSW 2006<br>Ph: +61 2 8627 1932<br>Fax: +61 2 8627 0141
Associate Investigators:	Professor Stephen Colagiuri,<br>Associate Professor Tania Markovic,<br>Associate Professor Amanda Salis,<br>Mr James Gerofi,<br>Ms Mackenzie Fong,<br>Dr Kathryn Williams,<br>Dr Namson Lau,<br>Dr Claire Madigan,<br>Dr Clare Manns,<br>Dr Jessica Swinbourne,<br>Associate Professor Kyoung Kon Kim,<br>Associate Professor Gareth Denyer,<br>Associate Professor Andrew Holmes,<br>Dr Stefanie Schurer,<br>Dr Agnieszka Tymula,<br>Ms Lisa Leung

Authors<br><br>Name and address	Dr Nicholas Fuller,<br>Professor Ian Caterson,<br>Professor Stephen Colagiuri,<br>Professor Deborah Schofield,<br>Associate Professor Andrew Holmes,<br>Associate Professor Tania Markovic,<br>Associate Professor Amanda Salis,<br>Associate Professor Kyoung Kon Kim,<br>Associate Professor Gareth Denyer,<br>Dr Kathryn Williams,<br>Dr Namson Lau,<br>Dr Claire Madigan,<br>Dr Stefanie Schurer,<br>Dr Agnieszka Tymula,<br>Professor John Cawley<br>The Boden Institute, Charles Perkins Centre D17 The University of Sydney NSW 2006
Study centre<br><br>Name, address and telephone number	The Boden Institute<br>Charles Perkins Centre D17<br>The University of Sydney NSW 2006<br>Ph: +61 2 8627 1932<br>Fax: +61 2 8627 0141
Biostatistician<br><br>Name and address	Dr Nicholas Fuller<br>The Boden Institute<br>The University of Sydney<br>Charles Perkins Centre D17<br>The University of Sydney NSW 2006<br>E: nick.fuller@sydney.edu.au<br>Ph: +61 2 8627 1932<br>Fax: +61 2 8627 0141

STUDY ACKNOWLEDGMENT/CONFIDENTIALITY

By signing this Protocol, the Investigator(s) acknowledges and agrees:

The Protocol contains all necessary details for conducting the study. The Investigator will conduct this study as detailed herein, in compliance with Good Clinical Practice (GCP) and the applicable regulatory requirements, and will make every reasonable effort to complete the study within the time designated.

The Protocol and all relevant information on the drug relating to pre-clinical and prior clinical experience, which was furnished by the Funding Body, SFI, will be made available to all physicians, nurses and other personnel who participate in the conducting of this study. The Investigator will discuss this material with them to assure that they are fully informed regarding the drug(s) and the conduct of the study.

This document contains information that is privileged or confidential. As such, it may not be disclosed unless specific prior permission is granted in writing by the Sponsor or such disclosure is required by federal or other laws or regulations. Persons to whom any of this information is to be disclosed must first be informed that the information is confidential. These restrictions on disclosure will apply equally to all future information supplied, which is indicated as privileged or confidential.

The Sponsor will have access to any source documents from which Case Report Form (CRF) information may have been generated. The CRFs and other data pertinent to this study are the sole property of the Sponsor.

The conduct and results of this study will be kept confidential. The results of this study may be published.

**Investigator Signatory:**<br>NAME AND TITLE:	Signature:<br>Date:

**Investigator Signatory:**<br>NAME AND TITLE:	Signature:<br>Date:

TABLE OF CONTENTS

STUDY ACKNOWLEDGMENT/CONFIDENTIALITY 3

TABLE OF CONTENTS 4

ABBREVIATIONS AND DEFINITIONS OF TERMS 9

1 Synopsis 11

2 Introduction 17

3 Objectives 24

4 Study Design 26

5 Study Population 28

5.1 Number of Participants 28
5.2 Inclusion Criteria 28
5.3 Exclusion Criteria 28
5.4 Other Eligibility Criteria Considerations 30
5.5 Study Duration 30

6 Study Assessments and Procedures 31

6.1 Screening Evaluation (Visit 1) (Day -7) 31
6.2 Study Procedures 32
6.2.1 Baseline Visit (Visit 2) (Day 0) 32
6.2.2 Treatment Visit (Visit 3) (Month 1) 33
6.2.3 Treatment Visit (Visit 4) (Month 2) 34
6.2.4 Treatment Visit (Visit 5) (Month 3) 34
6.2.5 Treatment Visit (Visit 6) (Month 4) 35
6.2.6 Treatment Visit (Visit 7) (Month 5) 35
6.2.7 Follow-Up Visit (Visit 8) (Month 6) 35
6.2.8 Follow-Up Visit (Visit 9) (Month 7) 37
6.2.9 Follow-Up Visit (Visit 10) (Month 8) 37
6.2.10 Follow-Up Visit (Visit 11) (Month 9) 37
6.2.11 Final Visit (Visit 12) (Month 12) 38
6.2.12 Termination Visit 38
6.3 Efficacy Assessments 39
6.3.1 SF-36 Health Survey 39
6.3.2 Assessment of Quality of Life-8D (AQOL-8D) Questionnaire 39
6.3.3 Patients' Global Impression of Change (PGIC) Scale 39
6.3.4 Pain Rating Scales 39
6.3.5 Weight Locus of Control (WLOC) Questionnaire 39

6.3.6 Target Weight (Goal Weight) ... 39
6.3.7 Goal Commitment Scale ... 40
6.3.8 Effort Scale ... 40
6.3.9 Expected Weight ... 40
6.3.10 General Self-Efficacy Scale ... 40
6.3.11 Five Factor Model and Vignettes ... 40
6.3.12 Risk and Time Preferences ... 40
6.3.13 Outcome-Expectancies Scale ... 41
6.3.14 Dissatisfaction Scale ... 41
6.3.15 Economics of Obesity ... 41
6.3.16 Dietary Intake ... 41
6.3.17 Accelerometer ... 41
6.3.18 Medication Compliance ... 41
6.3.19 Stool Collection Protocol ... 42
6.3.19.1 Sampling Schedule ... 42
6.3.19.2 Specimen Labelling ... 42
6.3.19.3 Materials Needed ... 43
6.3.19.4 Collection Method ... 43
6.3.20 Practitioners intuition ... 43
6.4 Study Restrictions ... 43
6.4.1 Dietary ... 43
6.4.2 Smoking and Alcohol Intake ... 44
6.4.3 Confinement ... 44
6.4.4 Concomitant Medication ... 45
6.5 Safety Assessments ... 45
6.5.1 Physical Examination ... 45
6.5.2 12-Lead ECG ... 47
6.5.3 Laboratory Safety Testing ... 48
6.5.3.1 Biochemistry ... 49
6.5.3.2 Haematology ... 49
6.5.3.3 Study Specific tests ... 49
6.5.3.4 Handgrip Strength test ... 49
6.5.3.5 Dual-energy X-ray absorptiometry (DXA) scan ... 50
6.5.3.6 Cognitive Function ... 51
6.5.3.7 Urinalysis ... 51
6.5.3.9 Microbiota Dynamics Assessment ... 52
6.5.3.10 Supra-gingival Plague Analysis 53

6.5.4 Adverse Events (AE) 52

7 Investigational product(s) (IP) 53

7.1 Description of Investigational Products (IP) 53

7.2 Dose Justification 53

7.3 Comparator Justification 54

7.4 Administration 54

7.5 Randomisation 55

7.6 Unblinding 56

7.7 Product Labelling 56

7.8 Handling and Storage of Study Drugs 57

7.9 Dispensing of Study Drugs 57

8 Adverse Events (AE) and Serious Adverse Events (SAE) 58

8.1 Definition of an Adverse Event 58

8.2 Definition of a Serious Adverse Event (SAE) 59

8.3 Clinical Laboratory AEs, and SAEs 60

8.4 Time Period, Frequency, and Method of Detecting AEs and SAEs 60

8.5 Recording of AEs and SAEs 60

8.6 Prompt Reporting of SAEs 61

8.7 Expeditable Events 62

8.8 Evaluating AEs and SAEs 62

8.8.1 Assessment of Intensity 62

8.8.2 Assessment of Causality 62

8.8.3 Assessment of Expectedness 63

8.9 Follow-up of AEs and SAEs 64

8.10 Post-study AEs and SAEs 64

9 Participant Completion and Discontinuation 65

9.1 Participant Completion 65

9.2 Stopping Rules / Discontinuation Criteria 65

9.3 Voluntary Participant Withdrawal 65

9.4 Early Termination of the Study 65

10 Case Report Form (CRF) 66

11 Data Analysis and Statistical Considerations 66

11.1 Hypotheses 66

11.2 Endpoints 66

11.3 Sample Size 66

11.4 Statistical Analysis 67

12 Data Collection and Management ... 68

13 Monitoring and Quality Assurance ... 68

13.1 Curriculum Vitae and Other Documentation ... 68

14 Investigator Responsibility ... 69

15 Study Report ... 69

16 Administrative Procedures ... 69

16.1 Ethical Considerations ... 69

16.2 Ethical Review Committee ... 70

16.3 Regulatory Authorities ... 70

16.4 Informed Consent ... 70

16.5 Participant Reimbursement ... 71

16.6 Emergency Contact with Investigators ... 71

16.7 Notification of Primary Care Physician ... 71

16.8 Investigator Indemnification ... 71

16.9 Payments ... 71

16.10 Protocol Amendments ... 72

16.11 Protocol Compliance ... 72

16.12 Archives: Retention of Study Records ... 73

16.13 Archives: Retention of Other Study Specific Samples ... 73

17 References 74

APPENDIX A Faecal Fat collection 80

APPENDIX B SF-36® Health Survey 90

APPENDIX C Assessment of Quality of Life (AQOL) 8D Questionnaire 93

APPENDIX D Patients' Global Impression of Change (PGIC) Scale 100

APPENDIX E Pain rating scales 101

APPENDIX F Weight Locus of Control (WLOC) Questionnaire 105

APPENDIX G Target Weight (Goal Weight) 106

APPENDIX H Goal Commitment Scale 107

APPENDIX I Effort Scale 108

APPENDIX J Expected Weight 109

APPENDIX K General Self-Efficacy Scale 110

APPENDIX L Five Factor Model and Vignettes 112

APPENDIX M Risk and Time Preferences 116

APPENDIX N Outcome-Expectancies Scale 119

APPENDIX O Dissatisfaction Scale 120

APPENDIX P Economics of Obesity 121

APPENDIX Q Participant Food Diary 125

APPENDIX R Australian Type 2 Diabetes Risk Assessment Tool 129

APPENDIX S Dental data collection sheet 132

APPENDIX T Oral questionnaire 133

List of tables

Table 4 Schedule of procedures for all treatment arms of the protocol 27

Table 6.3.19.1 Schedule for Microbiota Sampling and main study collection 42

Table 6.5.1 BMI Classification 47

Table 6.5.3.1 Laboratory tests conducted at each visit 48

Table 6.5.3.2 Laboratory tests conducted for the Microbiota Dynamics Cohort 49

List of figures

Figure 6.3.19.2 Specimen Labelling 40

Figure 7.4 Four arms of the trial 51

ABBREVIATIONS AND DEFINITIONS OF TERMS

®	Registered Product
ADA	American Diabetes Association
AE	Adverse Event
α-CD	α-cyclodextrin
ALT	Alanine Transaminase
AST	Aspartate Transaminase
BMI	Body Mass Index
CRF	Case Report Form
CK	Compound K
CTN	Clinical Trial Notification
DXA	Dual-energy X-ray Absorptiometry
ECG	Electrocardiogram
eGFR	Estimated Glomerular Filtration Rate
eCRF	Electronic Case Report Form
FBCx	α-cyclodextrin
GCP	Good Clinical Practice
GINST15	Panaxadiol Ginsenoside
HbA1c	Glycated Haemoglobin
HDL	High Density Lipoprotein
Hr	Hour
HREC	Human Research Ethics Committee
hsCRP	High-sensitivity C-reactive protein
IB	Investigators Brochure
IP	Investigational Product
ICH	International Conference on Harmonisation
LDL	Low Density Lipoprotein
ml	Millilitre
mmol/L	Millimole per Litre
NHMRC	National Health and Medical Research Council
QALY	Quality Adjusted Life Year
SAE	Serious Adverse Event
SF	Short Form

SLHD	Sydney Local Health District
SCFA	Short-Chain Fatty Acids
SPC	Study Product Containers
SFI	SOHO Flordis International
TSH	Thyroid Stimulating Hormone
TGA	Therapeutic Goods Administration

1 Synopsis

Study Title:	A double blinded, randomised controlled trial to determine<br>a) the efficacy of FBCx (a formula based on α-cyclodextrin (marketed under the trade name FBCx)) on cholesterol control, and<br>b) the efficacy of Ginst15 (a ginseng extract formula based on Compound K) on glycaemic control
Development Phase:	Phase III
Study Population:	Participants will be male or female aged ≥ 18 years, with a Body Mass Index (BMI) ≥ 25 kg/m^2, and have pre-diabetes as defined by the American Diabetes Association (ADA) guidelines.<br>Participants will be recruited from the current Boden Institute database, the Sydney Local Health District (SLHD) intranet, the University of Sydney website, the SFI Research Study website, social media, by advertising in local newspapers and radio stations, SFI Recruitment Database, SFI Clinical Registry, and electronic patient recruitment platforms Clariness, Clinlife and Trialfacts. Participants will also be recruited through health awareness stands, education sessions, editorial pieces, primary care, pharmacies and advertising on the Australian Clinical Trials website.
Study Drugs:	FBCx (a formula based on α-cyclodextrin) (2 X 1000 mgs three times daily) (Placebo matched)<br>Ginst15 (a ginseng extract formula based on Compound K) (2 X 160 mgs three times daily) (Placebo matched)
No. Participants:	400 evaluable Participants
Centre:	The Boden Institute
Study Duration:	12 months total (6 months intervention and 6 months follow- up)
Study Start:	January 2015
Study Completion:	July 2017

Study Endpoints:	The primary endpoints of this study is to determine:<br>• The efficacy of FBCx (a formula based on α-cyclodextrin (marketed under the trade name FBCx)) on cholesterol control, and<br>• The efficacy of Ginst15 (a ginseng extract formula based on Compound K) on glycaemic control.<br>The secondary endpoint of this study is to determine:<br>• The effect of each product and the combination of products on absolute weight loss (kg) and percentage of weight loss, relative to baseline body weight.

Study Objectives:<br><br>Primary and Secondary	Primary Objectives<br>1. To determine the efficacy of FBCx (a formula based on α-cyclodextrin (marketed under the trade name FBCx) on cholesterol control in an overweight or obese population with pre-diabetes.<br>2. To investigate the efficacy of Ginst15 (a ginseng extract formula based on Compound K) on glycaemic control in an overweight or obese population with pre-diabetes.<br><br>Secondary Objectives<br>1. The effect of each product and the combination of products absolute on weight loss (kg) and percentage of weight loss, relative to baseline body weight.<br>2. To investigate the difference in full lipid profile (including total cholesterol, HDL, LDL), apolipoprotein B, plasma glucose, and HbA1c between groups.<br>3. To compare the percentage of Participants in each category of glucose tolerance (normal, impaired fasting glucose, and type 2 diabetes) between groups.<br>4. To examine changes in body composition by waist circumference, fat mass, fat free mass, and blood pressure.<br>5. To investigate the difference in other biochemical parameters (liver enzymes and inflammatory markers) between groups.<br>6. To measure the side effect profile of each product using AE reporting.<br>7. To investigate the change in vitality between groups using the vitality scale of the SF 36 Health Survey.<br>8. To investigate the relationship between goals, outcome-expectancies, self-efficacy, commitment and weight change across all groups.<br>9. To investigate the relationship between unmet goals, effort, dissatisfaction and weight change across all groups.<br>10. To investigate the effect of the intervention type on changes in personality (Five Factor Model, self-efficacy) and preferences (risk and time).<br>11. To investigate whether efficacy of the intervention type depends on the change in personality/preferences.<br>12. To determine the cost-effectiveness of each intervention relative to the lifestyle programme alone by measuring the cost per QALY.<br>13. To determine whether weight loss results in better financial outcomes for Participants.<br>14. To measure the change in gut hormones and adipokines between groups.<br>15. To investigate the long-term outcome of the gut microbiota profile between groups.

Study Objectives:<br>Primary and Secondary	16. To compare the metabolic activity of gut microbiota between all groups (short chain fatty acids (SCFA) production in plasma and faeces).<br>17. To investigate the dynamics of the microbiota community as a result of dietary supplementation and elimination of FBCx and Ginst15 across all groups.<br>18. To determine the difference in handgrip strength throughout the study.<br>19. To determine circulating hormones or factors that influence muscle mass or strength, such as but not limited to insulin like growth-factor 1 (IGF-1), IGF-1 binding proteins and cortisol.<br>20. To evaluate the efficacy of different weight maintenance approaches in preventing weight regain after a weight loss phase.<br>21. To identify the behavioural strategies people use and the psychological processes that enable them to adopt weight management behaviours (prompted by regular self-weighing).<br>22. To determine whether practitioner's intuition predicts weight loss.<br>23. To measure the change in cognitive function between groups and by weight loss achieved (0±1%, 5-10%, >10% weight loss).<br>24. To identify if a change in diet modifies the oral health in obese people.
Study Design:	This will be a double-blinded, randomised controlled trial with a 2 x 2 factorial design.
Eligibility Criteria (Inclusion and Exclusion):	Participants will be considered for the study if they have the following:<br>• Aged ≥ 18 years of age.<br>• BMI ≥ 25 kg/m^2<br>• Pre-diabetes (determined by bloods at the Screening visit)<br>Pre-diabetes criteria are based on ADA guidelines. To be eligible the Participants must have:<br>- a fasting plasma glucose ≥ 5.6-6.9 mmol/L AND/OR<br>- 2 hour post-challenge (oral glucose tolerance test) plasma glucose ≥ 7.8-11.0 mmol/L AND/OR<br>- HbA1c ≥ 5.7-6.4%<br>Participants will be excluded for any of the following reasons:<br>• Type 2 diabetes<br>Type 2 diabetes criteria are based on ADA guidelines. Participants will be excluded from the study if they have:<br>- a fasting plasma glucose ≥ 7.0 mmol/L AND/OR<br>- 2hour post-challenge (oral glucose tolerance test) plasma glucose ≥ 11.1 mmol/L AND/OR<br>- HbA1c ≥ 6.5%

	• Use of anti-lipidaemic medications for cholesterol control<br>• Use of anti-diabetic medications for pre-diabetes<br>• Type 1 diabetes<br>• Unstable angina or recent onset of cardiovascular disease (within 1 month of Screening)<br>• Bariatric surgery
Eligibility Criteria (Inclusion and Exclusion):	• A history of significant liver, kidney or gastrointestinal disease AND/OR<br>o ALT or AST > 2.5 times upper limit of normal<br>o serum creatinine > 1.5 times upper limit of normal or<br>o eGFR < 60ml/min/1.73m^2 or presence of microalbuminuria<br>• Chronic diarrhoea, bowel motility problems, or other conditions that could affect intestinal fat absorption<br>• Untreated thyroid disease<br>• Greater than 10% change in body weight over the past 3 months<br>• Alcohol or illicit drug abuse<br>• Pregnant or breastfeeding women, and women who might be planning pregnancy during the duration of the study<br>• Use of weight loss medications and other drugs that may affect body weight e.g. anti-psychotics, anti-depressants, or corticosteroids<br>• Taking the following medications which may show reduced absorption of the investigational products: antibiotics, anticoagulants, anticonvulsants, antiarrhythmics, immunosuppressants, or any other drug that is necessary to take with a meal. Short-term and prophylactic antibiotics may be taken during study participation for up to 14 days, but they should be taken at least 2 hours apart from the study drug<br>• Commencement of a new prescription medication within 3 months of Screening or change in dose regimen of a prescription medication within 1 month of Screening<br>• A history or presence of malignancy [completely resected basal or squamous cell carcinoma of the skin if treatment completed > 6 months prior to enrolment and Participant is in remission for > 5 years prior to Screening remain eligible]<br>• Inability to read and write English<br>• A history of frequently changed smoking habits, in addition to smoking cessation within 6 months prior to Screening. Those who wish to take on the advice of a 'Quit' smoking programme at the time of Screening will be eligible to start the trial after 6 months<br>• Participants may also be excluded, if in the opinion of the study Investigators, they have some other condition or disorder that may adversely affect the outcome of the study or the safety of the Participant

	• Participation in a clinical trial in the last month<br>• Unable to commit to the appointment schedule or perform the tasks required in the study.
Study Intervention:	Participants will be randomised to one of four groups. Each Participant will be enrolled in the lifestyle programme. The initial six months will be focused on weight loss. The weight loss diet (total fat intake of < 30% and saturated fat < 10% of total energy) will be hypo-caloric with a 500 kilocalorie deficit (based on the Harris Benedict Equation for estimating energy requirements). All Participants will continue to be followed up by onsite assessments at months 9 and 12 and will be randomised to a weight loss maintenance intervention at month 6.
Safety Parameters:	Adverse Event and Serious Adverse Event monitoring.
Laboratory Parameters/Analysis:	TSH, Creatinine, eGFR, FBC, Fasting glucose, HbA1c, Full lipid profile, Liver enzymes, hsCRP, Apolipoprotein B, plasma SCFA, faecal SCFA, and microbiota community 16s rDNA profile.
Total Blood Volume:	~ 50 ml of blood
Sample Size Determination:	The study is powered for the smallest difference between two of the groups for each outcome, with the larger sample size being adopted.
Statistical Analyses:	Data will be analysed for normality using the Shapiro-Wilk test. For analysis of differences between treatments, an analysis of co-variance (ANCOVA), or the non-parametric equivalent will be used. For within group changes, repeated measures ANOVA will be used. As well as an analysis of completers (those that attend the final visit), an intention to treat (ITT) analysis will also be included. For the ITT analysis, dropouts will be treated using a variety of sensitivity analyses.

2 Introduction

Type 2 diabetes mellitus is the fastest growing chronic illness in Australia, and over 3.2 million people are estimated to have pre-diabetes and diabetes, with a further 275 individuals being diagnosed with diabetes every day. As diabetes is the 6th leading cause of death in Australia, interventions to prevent this condition and its complications are a priority for Australian society.

Alpha-Cyclodextrin

Dietary fibres have long been known to bind to dietary fat; typically on a 1:1 or perhaps as high as a 1:1.4 ratio. Such small ratios make the common dietary fibres impractical as an aid for weight loss and would require the individual to ingest very large amounts of fibre to remove a significant amount of dietary fat and this is not practical. Therefore, there is importance in finding a more efficient fat binder. One such example is FBCx (a formula based on α-cyclodextrin (α-CD)), a polysaccharide, derived from corn, of six glucose units that are covalently attached end to end via α-1, 4 linkages. Artiss and colleagues (2006) demonstrated that α-CD could bind triglyceride and at ratios that are significantly different from the 1:1 that is typical for dietary fibres. This animal study demonstrated that α-CD has the unique ability to complex with and prevent the absorption of 9 times its own weight in dietary fat. It appears that α-CD forms a very stable complex or emulsion with the dietary fat in the stomach and remains bound to the fat through the gastro-intestinal (GI) tract, first preventing the absorption of the bound fat in the small intestine and then fermentation by the intestinal flora.

Previous research examining the effect of α-CD on weight loss has shown that is effective in reducing and/or maintaining body weight in obese patients with type 2 diabetes, without the need for reduced energy intake or an increased exercise programme (Grunberger, 2007). In this study both cholesterol and triglyceride levels were reduced in those who had hyper-triglyceridaemia (Grunberger, 2007). There was no change in high density lipoprotein (HDL) cholesterol levels. Furthermore, significantly elevated adiponectin levels indicated an increase in insulin sensitivity which might delay the onset of usage of insulin and/or reduce the amount of supplemental insulin required in these participants (Grunberger, 2007). Another study has also shown reduced atherogenic lipoproteins (apolipoprotein-B) with the supplementation of α-CD in healthy overweight individuals (Comerford, 2011).

Supplementation of α-CD potentially alters the activities and growth of certain microbiota in the gut, such as known cyclodextrin utilizer *B. thetaiotaomicron*. Following oral administration, α-CD is fermented by intestinal microbiota to short chain fatty acids (mainly acetate, propionate, and butyrate) prior entering the circulation (Van Ommen, 2004).

Hydrolysis by gut microbiota may lessen the fat-binding benefits of α-CD and therefore may alter its efficacy in promoting weight loss.

Compound K

The root of ginseng has been used for remedies in traditional Chinese medicine, most recently for improving glycaemic control. The pharmacological properties of ginseng are mainly attributed to the ginsenosides, the active components found in the extracts of different species of ginseng (Attele, 1999). Numerous studies have been conducted and found that these have anti-diabetic properties (Yokozawa, 1985; Attele, 2002; Vuksan, 2005). Ginst15 (a ginseng extract formula based on Compound K) (CK), which is a final metabolite of protopanaxadiol ginsenosides following a hydrolysis by intestinal microbiota (Akao, 1998; Tawab, 2003), has been shown to be the active ingredient for this anti-diabetic action. CK enhances insulin secretion by acting directly on the pancreas (presumably). Significant improvements have been shown in both plasma glucose and insulin levels in diabetic *db/db* mice when treated with CK and metformin combination therapy versus metformin therapy alone (Yoon, 2007).

As an adjunct to the trial with respect to the current treatment for overweight and obesity, blood (to measure gut hormones and adipokines) and stool samples (to measure gut microbiota) will be collected at specified time points. It has been shown that energy restricted diets have a negative effect on the circulating mediators of appetite, which encourages weight regain (Sumithran, 2011) and therefore long-term strategies to counteract this change are needed to prevent obesity relapse. Furthermore, with the advances in molecular biology techniques, the association between changes in the gut microbiota with diet and human diseases or disorders is becoming more evident. Of particular relevance to this study, the composition of the colonic microbiota and the balance of its metabolic products are strongly influenced by degree of weight loss, by diet, and in particular, by the intake of resistant carbohydrates and dietary fibre (Scott, 2008).

Based upon these properties of α-CD and CK, the current double blinded, randomised controlled trial is designed to investigate the beneficial effects of these products in overweight and obese participants with pre diabetes. It is proposed that these products may over-lap in their function and therefore are being compared in the same trial in the prevention of type 2 diabetes.

Weight loss goals

There is a debate in the literature regarding the impact of goals on participants' weight loss in behavioural weight loss treatments (Stubbs, 2011; Linde, 2004; Foster, 1997; Foster, 2004; Wadden, 2004). One perspective postulates that unrealistic, high goals are

detrimental to weight loss. On the contrary, some researchers have suggested that larger goals may actually stimulate larger weight losses. This perspective is based on empirical research reporting a positive linear relationship between goals and performance outcomes (Locke, 1990; Locke, 2002). At present, the evidence is inconsistent regarding the relationship between goals and weight loss in overweight and obese individuals participating in treatment. Given these discrepancies, it is plausible that other psychological processes are involved in this relationship. Goal-Setting Theory (Locke, 1990, Locke, 2002) acknowledges that although high goals may be related to superior performance outcomes, there are other important factors in this relationship including an individual level of self-efficacy, outcome-expectancies and commitment to achieving that goal. To date, previous research has been compromised by the common failure to address the moderating impact of these important constructs within the relationship between goals and weight loss.

The theoretical relationship between effort, dissatisfaction, negative behavioural consequences and goal abandonment has also not been carefully investigated in a weight loss context. Most participants in weight loss treatments aim to lose more weight than is characteristic of those interventions and as a result, in general, participants do not meet their goals (Foster, 1997; Foster, 2004; Jeffrey, 1998; Wadden, 2003). Research in the broader psychological literature has demonstrated that the pursuit of unattainable goals can lead to negative behavioural consequences including goal abandonment and depressive focusing style (Carver, 1990; Cervone, 1993; Higgins, 1987). Further research is needed to better understand these relationships.

The aim of this sub-study is two-fold: To test goal-setting theory within a weight loss context and then to better understand the relationship between unmet goals, dissatisfaction and negative behavioural consequences.

Weight loss maintenance

Although there are successful interventions that help people to lose weight many people regain the weight they have lost and thus weight maintenance remains a critical challenge. The period after initial weight loss is when people are at highest risk of weight gain. Few people (1 in 10) recover from even minor lapses of 1–2 kg of regain in weight (Wing, 2006) Therefore preventing small regains from turning into larger relapses appears critical for weight loss maintenance.

A systematic review of randomised controlled trials of weight loss maintenance interventions for obese adults identified that the greatest need for further research was in the area of lifestyle interventions focused on supporting people to manage their weight in the longer term by regulating food intake and increasing physical activity (Dombrowski, 2014). There were four important features of effective weight maintenance interventions,

all of which are consistent with self-regulation theory; these being goal setting, self-monitoring of weight and behaviour, action plans for weight control through dietary and physical activity behaviours and plans on how to cope with risk factors for weight regain and relapse prevention.

Self-weighing and accountability

The potential efficacy of self-weighing is based on the principles of self-regulation theory. Self-regulation has been described as a process that has three distinct stages; self-monitoring, self-evaluation and self-reinforcement. The awareness fostered during self-monitoring is considered an essential initial step in promoting and sustaining behaviour change. Self-monitoring in the context of self-weighing can show individuals how their behaviour affects their weight and allows them to adjust their behaviour to achieve their goals. It is simple for a health professional or public health communication to advocate and it is simple for people to understand and implement. It is the kind of behaviour that could become habitual. Trials have shown that participants can adhere to daily self-weighing (Steinberg, 2013). Presently one trial in the UK is investigating the effectiveness of self-weighing for weight loss maintenance (Madigan, 2015). Participants in the intervention group are asked to weigh themselves daily and record their weight. They are also sent generic reminder text messages and receive three phone calls to encourage self-weighing (Madigan, 2015). The participants are given scales that send the weight through a cellular system to a database in real time. In this trial the scales are being used as an objective measure of self-weighing and the participants are told their weights will not be checked. That said, participants often report feeling pleased that someone will be watching their weight and perceive they are accountable to someone. This sense of accountability may help people to stay on track with their weight maintenance or it may act in combination with self-weighing as an effective strategy. A systematic review of self-weighing for weight loss found that in sub group analyses, trials with accountability had greater weight loss (Madigan, 2015). However no trial has directly assessed this.

No previous study has collected data about how people use the reading on the scale as a tool to change their physical activity or dietary behaviours. In a small subgroup of participants this will be examined by collecting data about their thoughts and behaviours when they step on the scales and reflect on their weight.

The aim of this sub-study is to evaluate the effectiveness of the concept of accountability on weight regain six months after completing a weight loss programme and to examine the behavioural strategies used in combination with self-weighing.

Effect of weight-loss and maintenance programmes on change in patterns of thought

Both our weight-loss and maintenance programmes are likely to change the way our patients think about themselves and their environment. Patterns of thought – embodied in personality traits and preferences - have been linked frequently to healthy behaviours and obesity (e.g. Cobb-Clark, 2014; Kern, 2009; Courtemanche, 2014; Ikeda, 2010; Smith, 2005; Yamada, 2013). The literature is still divided on whether such patterns of thought are fixed in adulthood and whether interventions can change them (Cobb-Clark, 2012, 2013; Schurer, 2015; Tymula, 2013).

In this sub-study we assess whether the programmes are successful in changing otherwise stable patterns of thought and beliefs, whether the success of these programmes depends on (a) the initial level of patterns of thought of a patient, or (b) the patient's ability to change his or her patterns of thought as the intervention proceeds.

Knowledge of the magnitude of change in patterns of thought can allow us to calculate the wider economic benefits of our intervention programmes in terms of gained hours in the labour market and reduced health care costs. This can be done by calculating by how many standard deviations measured patterns of thought - derived from standard instruments such as the Five Factor Model and risk and time preferences– increase or decrease, and then using this empirically derived magnitude of change to calculate the gains in productivity due to a change in these patterns of thought provided in previous studies (See Cobb-Clark, 2012; 2013). For instance, if our weight maintenance intervention increases self-efficacy – one of many proxies of patterns of thought – by 1 standard deviation, then we know from the previous literature that such a change implies a 0.1 standard deviation increase in hourly wages in the Australian context (See Cobb-Clark, 2011).

To be able to most objectively measure these patterns of thought, we collect data on Five Factor Model (Conscientiousness, Openness to Experience, Agreeableness, Extraversion, Neuroticism) and questions about the personality of hypothetical persons to control for scaling bias in self-assessments. These were developed by Stefanie Schurer and Rebecca Edwards adapted from Bolt et al. (2014) for the purpose of a student survey that is currently underway at the University of Sydney (See www.sydney.edu.au/economics/survey). To control for gender biases in the evaluation of hypothetical persons, we randomly assign female and male gender to each of the three vignettes a patient is asked to evaluate. We will also collect task-based measures of risk and time preferences. We propose to use standard tasks from economics developed by Holt & Laury (2002) and Andreoni & Sprenger (2012) to measure risk and time preferences in obese participants to verify whether the intervention affects these decision-making fundamentals that have been previously identified to correlate with being overweight.

Economics of obesity

There is a large literature in economics that finds that heavier individuals are less likely to be employed and, when employed, tend to earn less (see e.g. Cawley, 2004; Averett, 2011). (This disadvantage is greater for women than men). Some studies have been conducted that seek to measure whether this relationship is causal; that is, whether being heavier lowers wages. These studies rely on natural experiments, which have some advantages but also have the disadvantage that they are not based on a randomized controlled trial. In this study we will also seek whether weight loss is followed by improvements in employment and wages or salary. In addition, we seek to measure the mechanism of any such changes: specifically, whether the weight loss results in fewer days missed due to poor health, or less discrimination in the workplace on the basis of the subject's weight. Collectively, these results will greatly improve our understanding of whether weight lowers employment and wages, and if so, whether it is due to poor health or employer discrimination.

Can practitioners predict who will do well at weight loss?

Many practitioners will have an intuition of those who will do well on a weight loss programme or those who may not lose weight. However it is not known whether such intuition is valid or whether it is misconceived. This intuition is based on the behaviour of participants and therefore it is important to identify what factors are taken into account to make this judgement. As an observational study it will be determined whether practitioners intuition predicts weight loss and what factors influence this perception.

Effect of weight loss on cognitive function

A recent meta-analysis reported that obesity in mid-life is associated with lower brain volume and that it also increases the risk of Alzheimer's disease and vascular dementia by nearly 100% (Loef, 2013; Meg, 2014). Despite evidence showing that obesity-related neuro-cognitive risk factors (for example, deficits in working memory, learning, psychomotor speed and executive function) can lead to dementia, literature investigating if neuro-cognitive functions are altered after undergoing a weight loss intervention is sparse.

A recent systematic and meta-analytic review which investigated the effectiveness of intentional weight loss on cognitive function in overweight and obese adults identified the possible mechanistic role of insulin resistance in cognitive decline. Greater insulin resistance was associated with an Alzheimer disease-like pattern or reduced cerebral glucose metabolic rate in adults with pre-diabetes or newly diagnosed type 2 diabetes mellitus (Siervo, 2011). Therefore, lifestyle weight loss interventions may be effective strategies to improve cognition and, therefore, prevent Alzheimer disease, but further studies are warranted to establish this link. The authors of this review (Siervo, 2011) also

noted a lack of randomised controlled clinical trials (only two) in this field, and that stability of improved cognitive function following weight loss had not yet been investigated. Hence, it is unknown whether the beneficial effects of weight loss can be extended to improvements in cognitive function and is an area that warrants further research.

With respect to the specific complementary medicines being investigated in this trial, ginseng has shown promise of a cognitive enhancing effect (Lee, et al., 2008), but recent systematic reviews have highlighted the need for randomised, double-blind, placebo-controlled trials with large sample sizes to investigate the effect of ginseng in different populations (Geng et al., 2010; Lee et al., 2009).

This proposed research outcome will assess the change in cognitive function between groups and the association between weight loss and cognitive function. This study will also investigate weight loss responders (≥5% of weight loss, and ≥10% of weight loss) to those who lose no weight (0±1%), to establish whether there is a certain weight loss that corresponds to improvements in cognitive function. The research is this field is sparse and this will be a world first pilot study conducted in those with obesity and pre-diabetes with a longer-term follow-up of 12 months.

<u>Association of diet and oral health in obesity.</u>

Periodontitis is a silent or asymptomatic condition, characterized by destruction of periodontal tissues and ultimately loss of teeth (Dye, 2012). It is associated with various chronic conditions (diabetes mellitus, cardiovascular diseases and aspiration pneumonia) and has a negative impact on a person's quality of life (Buset et al., 2016, Genco and Genco, 2014). The prevalence of periodontitis in the Australian population is reported to be 24.5% (Roberts-Thomson and Do, 2007).

Obesity is also a risk factor for periodontitis (Suvan et al., 2011, Nascimento et al., 2016) and is associated with increased levels and proportions of periodontal pathogens, especially in patients with periodontitis (Maciel et al., 2016). However, the explanatory factor between obesity and periodontitis is yet to be discovered. Obese individuals with periodontitis have increased levels of pro-inflammatory cytokines as compared to obese individuals without periodontitis and non-obese individuals (Zimmermann et al., 2013, Pradeep et al., 2015).

Diet is a modifiable risk factor for obesity (El Ghoch et al., 2016). We hypothesize that diet is associated with both obesity and periodontitis, influences the composition of dental plaque microflora and helps to explain the association between obesity and periodontitis.

3 Objectives

Primary Objectives

1. To determine the efficacy of FBCx (a formula based on ọ-cyclodextrin (marketed under the trade name FBCx)) on cholesterol control in an overweight or obese group with pre-diabetes.
2. To investigate the efficacy of Ginst15 (a ginseng extract formula based on Compound K) on glycaemic control in an overweight or obese group with pre-diabetes.

Secondary Objectives

1. The effect of each product and the combination of products absolute on weight loss (kg) and percentage of weight loss, relative to baseline body weight.
2. To examine the changes in full lipid profile (including total cholesterol, high-density lipoprotein (HDL), low-density lipoprotein (LDL)), apolipoprotein B, plasma glucose, and glycated haemoglobin (HbA1c) between groups.
3. To compare the percentage of Participants in each category of glucose tolerance (normal, impaired fasting glucose, and type 2 diabetes) between groups at study completion.
4. To examine changes in body composition by waist circumference, fat mass, fat free mass, and blood pressure between groups.
5. To examine the changes in other biochemical parameters (liver enzymes and inflammatory markers) between groups.
6. To measure the side effect profile of each product using adverse event (AE) reporting.
7. To investigate the change in vitality between groups using the vitality scale of the short form (SF) SF-36 Health Survey.
8. To investigate the relationship between goals, outcome-expectancies, self-efficacy, commitment and weight change across all groups
9. To investigate the relationship between unmet goals, effort, dissatisfaction and weight change across all groups.
10. To investigate the effect of the intervention type on changes in personality (Five Factor Model, self-efficacy) and preferences (risk and time).
11. To investigate whether efficacy of the intervention type depends on the change in personality/preferences.
12. To determine the cost-effectiveness of each intervention relative to the lifestyle programme alone by measuring the cost per quality adjusted life year (QALY).

13. To determine whether weight loss results in better financial outcomes for Participants
14. To measure the change in gut hormones and adipokines between groups.
15. To investigate the long-term outcome of the gut microbiota profile between all groups.
16. To compare the metabolic activity of gut microbiota between groups (short chain fatty acids (SCFA) production in plasma and faeces).
17. To investigate the dynamics of the microbiota community as a result of dietary supplementation and elimination of FBCx and Ginst15 across all groups.
18. To determine the difference in handgrip strength throughout the study.
19. To determine circulating hormones or factors that influence muscle mass or strength, such as but not limited to insulin like growth-factor 1 (IGF-1), IGF-1 binding proteins and cortisol.
20. To evaluate the efficacy of three follow up programmes in preventing weight regain after a weight loss phase.
21. To identify the behavioural strategies people use and the psychological processes that enable them to adopt weight management behaviours (prompted by regular self-weighing).
22. To determine whether practitioners' intuition predicts weight loss.

23. To measure the change in cognitive function between groups and by weight loss achieved (0±1%, 5-10%, >10% weight loss).

24. To identify if a change in diet modifies the oral health in obese people.

4 Study Design

The study will be a single site Investigator initiated and designed, double blinded, randomised controlled trial conducted in Sydney, Australia.

The Boden Institute will recruit 400 evaluable Participants. Recruitment will take place through the existing Boden Institute database via email, the Sydney Local Health District (SLHD) intranet, the University of Sydney website, the SFI Research Study website (Participants will be required to complete the “Australian Type 2 Diabetes Risk Screening Assessment Tool” (Appendix R) to determine their pre-diabetic eligibility), social media, and advertising in local newspapers and radio stations. Participants will also be recruited through editorial pieces, health awareness stands, education sessions, primary care, pharmacies and advertising on the Australian Clinical Trials website.

Due to the number of expected participants and the stringent eligibility criteria of the study, SFI will be assisting with the recruitment through regular newsletters to GPs on their recruitment database, and to Participants who have previously expressed their consent for their details to be kept for future clinical trials (SFI Clinical Registry). Electronic patient recruitment platforms Clariness, Clinlife and Trialfacts will also be employed for participant recruitment.

Interested Participants will be provided with an information sheet containing details on the study (Participant Information Statement).

Potential Participants will be phone screened. The “Australian Type 2 Diabetes Risk Assessment Tool” (Appendix R) and a Participant’s most recent pathology results will be used to determine pre-diabetic eligibility for the study. Providing they do not meet any of the exclusionary criteria, they will then be asked to attend a Screening visit with one of the Investigators. Potential Participants who do not have blood results within the last 6 months will be asked to fast for this visit and a blood sample will be taken.

The potential Participant will give written consent before any procedures are performed. Please refer to “Table 4 - Schedule of procedures” for an exact description of the procedures that will take place at each visit.

Table 4 Schedule of procedures for all treatment arms of the protocol

Visit Number	1	2	3	4	5	6	7	8	9	10	11	12
**Month**	-1 wk	Day 0	1	2	3	4	5	6	7	8	9	12
**Days**	Screen	Baseline	28 +/-7	56 +/-7	84 +/-7	112 +/-7	140 +/-7	168 +/-7	196 +/-7	224 +/-7	273 +/-7	357 +/-7
Consent, Height, Medical History	✓											
Urine collection*	✓				✓			✓				✓
Body Weight	✓	✓	✓	✓	✓	✓	✓	✓			✓	✓
Waist Circumference	✓	✓	✓	✓	✓	✓	✓	✓			✓	✓
Electrocardiogram	✓											
Blood Pressure and Heart Rate	✓				✓			✓			✓	✓
Blood collection	✓				✓			✓				✓
Handgrip strength test		✓			✓			✓				✓
Dispense stool collection kit (main study)	✓						✓				✓	
Stool collection (main study)		✓						✓				✓
Dispense stool collection kit (Microbiota Dynamics Cohort)		✓	✓					✓	✓			
Weekly stool collection (Microbiota Dynamics Cohort)			✓	✓					✓	✓		
DXA Scan		✓						✓				✓
Cognitive function tests	✓						✓					✓
Dispense study compound		✓	✓	✓	✓	✓	✓					
Reconcile study compound			✓	✓	✓	✓	✓	✓				
Dietitian review		✓	✓	✓	✓	✓	✓	✓			✓	✓
Completion of questionnaires		✓						✓				✓
Record current/new medications	✓	✓	✓	✓	✓	✓	✓	✓			✓	✓
AE monitoring		✓	✓	✓	✓	✓	✓	✓			✓	✓
Oral examination		✓						✓				
ESTIMATED TIME (minutes)	90	205	40	40	120	40	70	205	5	5	30	195

* For those of childbearing potential.

5 Study Population

5.1 Number of Participants

A total of 400 evaluable eligible Participants will be recruited into the study. The study is powered for the smallest difference between two of the groups for each outcome, with the larger sample size being adopted (See 11.3 - Sample Size).

5.2 Inclusion Criteria

Participants will be considered for the study if they meet the following criteria:

- Aged ≥ 18 years of age
- BMI ≥ 25 kg/m^2
- Pre-diabetes (determined by blood test results at the Screening visit or 6 months prior to Screening)

To be eligible for the pre-diabetes criteria based on American Diabetes Association (ADA) guidelines, Participants must have:

- a fasting plasma glucose ≥ 5.6-6.9 mmol/L AND/OR
- 2 hour post-challenge (oral glucose tolerance test) plasma glucose ≥ 7.8-11.0 mmol/L AND/OR
- HbA1c ≥ 5.7-6.4%

5.3 Exclusion Criteria

Participants will be excluded for any of the following reasons:

- Type 2 diabetes

The criteria for the diagnosis of type 2 diabetes will also be based on ADA guidelines. Participants will be excluded from the study if they have:

- a fasting plasma glucose ≥ 7.0 mmol/L AND/OR
- 2 hour post-challenge (oral glucose tolerance test) plasma glucose ≥ 11.1 mmol/L AND/OR
- HbA1c ≥ 6.5%

- Use of anti-lipidaemic medications for cholesterol control
- Use of anti-diabetic medications for pre-diabetes
- Type 1 diabetes

- Unstable angina or recent onset of cardiovascular disease (within 1 month of Screening)
- Bariatric surgery
- A history of significant liver, kidney or gastrointestinal disease AND/OR
    - ALT or AST > 2.5 times upper limit of normal
    - serum creatinine > 1.5 times upper limit of normal or
    - eGFR < 60ml/min/1.73m^2 or presence of microalbuminuria
- Chronic diarrhoea, bowel motility problems, or other conditions that could affect intestinal fat absorption
- Untreated thyroid disease
- Greater than 10% change in body weight over the past 3 months
- Alcohol or illicit drug abuse
- Pregnant or breastfeeding women, and women who might be planning pregnancy during the duration of the study
- Use of weight loss medications and other drugs that may affect body weight e.g. anti-psychotics, anti-depressants, or corticosteroids
- Taking the following medications which may show reduced absorption of the IPs: antibiotics, anticoagulants, anticonvulsants, antiarrhythmics, immunosuppressants, or any other drug that is necessary to take with a meal. Short-term and prophylactic antibiotics may be taken during study participation for up to 14 days, but they should be taken at least 2 hours apart from the study drug
- Commencement of a new prescription medication within 3 months of Screening or change in dose regimen of a prescription medication within 1 month of Screening
- A history or presence of malignancy [completely resected basal or squamous cell carcinoma of the skin if treatment completed > 6 months prior to enrolment and Participants in remission for > 5 years prior to Screening remain eligible
- Inability to read and write English
- A history of frequently changed smoking habits, in addition to smoking cessation within 6 months prior to Screening. Those who wish to take on the advice of a 'Quit' smoking programme at the time of Screening will be eligible to start the trial after 6 months
- Participants may also be excluded, if in the opinion of the study Investigators, they have some other condition or disorder that may adversely affect the outcome of the study or the safety of the Participant
- Participation in a clinical trial in the last month

- Unable to commit to the appointment schedule or perform the tasks required in the study.

5.4 Other Eligibility Criteria Considerations

To assess any potential impact on Participant eligibility with regard to safety, the Investigator must refer to the Investigational Brochure (IB) for detailed information regarding warnings, precautions, contraindications, AEs, and other significant data pertaining to the investigational product (IP) being used in this study.

5.5 Study Duration

The study will be of 12 months total duration. There will be six months of active intervention during the treatment phase in one of four groups (FBCx + placebo, placebo + Ginst15, FBCx + Ginst15, and combined placebo). After the 6 month active intervention, Participants will be randomised to one of three weight maintenance programmes and followed up for a further 6 month period, making the study a total of 1 year duration.

6 Study Assessments and Procedures

The Participants who have not met any of the exclusion criteria during the telephone screening will attend the Screening Evaluation. The Participant will be required to give written consent before any procedures are performed. A copy of the Participant Information Statement and Participant Consent Form will be emailed to the Participant prior to the Screening visit as well as being given to the Participant at the Screening visit. They will be provided ample time to read the Participant Information Statement and ask any questions relating to the study.

The following measurements and procedures will be performed as part of the Screening visit:

6.1 Screening Evaluation (Visit 1) (Day -7)

At Screening, (1 week prior to Baseline visit), the following measurements and personal history will be recorded:

- Consent
- Height
- Medical history (including any forthcoming procedures)
- Body weight
- Waist circumference
- Electrocardiogram (ECG)
- Blood pressure and heart rate
- Concomitant medications

Medical history should include specific questions on cardiac history, angina, breathlessness, swollen ankles, sleep apnoea, urinary incontinence, impaired fertility, depression, anxiety and functional limitations such as joint pain and decreased mobility.

Their pre-diabetes status will also be confirmed at the Screening visit. The following laboratory samples will be taken:

- Fasting blood glucose
- Glycated Haemoglobin (HbA1c)
- Full lipid profile (total cholesterol, HDL-cholesterol, LDL-cholesterol, total cholesterol: HDL ratio, triglycerides)
- Apolipoprotein B
- High-sensitivity C-reactive protein (hs-CRP)
- Liver enzymes

- Creatinine and eGFR calculation (for those who do not have results within 6 months of Screening)
- Thyroid stimulating hormone (for those who do not have results within 6 months of Screening)
- Full blood count (for those who do not have results within 6 months of Screening)
- Gut hormones and adipokines – ghrelin, leptin, adiponectin, cholecystokinin, glucagon-like peptide-1
- Urine collection (female of child bearing potential)
- Cognitive function tests (substudy)

All Participants in each arm will be given the following:

- Instructions for Stool Specimen Collection
- Stool Collection Kit
- Food Diary
- Accelerometer pedometer

All Participants will be given a food diary that is to be completed prior to attending the Baseline visit. All Participants will be asked to record their dietary intake for three days (2 working days and 1 weekend day).

All Participants will be provided with an accelerometer pedometer, instructed how to use it as this will be used to measure their activity level for five days before the Baseline visit.

6.2 Study Procedures

If any Participant does not meet the inclusion criteria they will be recorded as a screen failure. If the Participant meets all of the inclusion criteria and none of the exclusion criteria, they will be booked in for a Baseline appointment.

6.2.1 Baseline Visit (Visit 2) (Day 0)

At the Baseline visit (7 days after Screening+/-1), the following activities for all Participants will occur:

- Body weight
- Waist circumference
- Dispense Stool Collection Kit (Microbiota Dynamics Cohort)
- Collect stool specimens (All Participants - Main Study)
- Dual-energy X-ray absorptiometry (DXA) scan

- Handgrip strength test
- Dispensing of 1 month supply of supplement (active or placebo)
- Dietitian review, collection of diet diary and dispensing of new diary
- Completion of questionnaires
- Review and record changes to concomitant medications and dose
- Oral examination and questionnaires (substudy)
- AE monitoring

Participants will receive a 1 month supply of supplements at Baseline, Month 1, 2, 3, 4 and 5 visits.

At the Baseline visit (1 week after Screening), all Participants will meet the study Dietitian for advice on their individual hypo-caloric diet for weight loss as well as when and how often to take the study supplement (placebo or active). Advice will also be given on other healthy lifestyle measures including behavioural change, alcohol intake, and smoking.

The dental staff will carry out an oral examination on all participants who have consented to be in the sub-study. The participants will be asked to complete a questionnaire (Appendix S) regarding their oral habits/perceptions and to undergo an oral examination of all their teeth. Name, age, gender and postcode (rural/urban dweller) and the number of decayed, missing and filled teeth (DMFT) will be recorded on a chart (Appendix T). The health of the participant's gums will be assessed using the computerised charting on the Florida Probe System. Supra-gingival plaque samples will be collected, kept at -80°C and analysed for microbiological identification.

During the initial six months of the study, all Participants will be encouraged not to change their medication usage to determine the true effect of the study interventions. A thorough medication review will take place again at three months to ensure adherence to good clinical management and practice for all the Participants.

As the study is blinded, all Participants in the study have the same evaluations at each visit.

The dietitian will also record how much weight loss the participant will achieve at 6 months and the reasons why they believe this to be the case.

6.2.2 Treatment Visit (Visit 3) (Month 1)

At Visit 3 (28 days after the Baseline visit +/-7), the following activities will occur:

- Body weight

- Waist circumference
- Dispense Stool Collection Kit (Microbiota Dynamics Cohort)
- Collect stool specimens (Microbiota Dynamics Cohort)
- Dispensing of 1 month supply of supplement (active or placebo)
- Reconcile returned study supplements
- Dietitian review, collection of diet diary and dispensing of new diary
- Review and record changes to concomitant medications and dose
- AE monitoring

6.2.3 Treatment Visit (Visit 4) (Month 2)

At Visit 4 (56 days after the Baseline visit +/-7), the following activities will occur:

- Body weight
- Waist circumference
- Collect stool specimens (Microbiota Dynamics Cohort)
- Dispensing of 1 month supply of supplement (active or placebo)
- Reconcile returned study supplements
- Dietitian review, collection of diet diary and dispensing of new diary
- Review and record changes to concomitant medications and dose
- AE monitoring

6.2.4 Treatment Visit (Visit 5) (Month 3)

At Visit 5 (84 days after the Baseline visit +/-7), the following activities will occur:

- Urine collection (female of child bearing potential)
- Body weight
- Waist circumference
- Blood pressure and heart rate
- Blood collection
- Handgrip strength test
- Dispensing of 1 month supply of supplement (active or placebo)
- Reconcile returned study supplements
- Dietitian review, collection of diet diary and dispensing of new diary
- Review and record changes to concomitant medications and dose
- AE monitoring

6.2.5 Treatment Visit (Visit 6) (Month 4)

At Visit 6 (112 days after the Baseline visit +/-7), the following activities will occur:

- Body weight
- Waist circumference
- Dispensing of 1 month supply of supplement (active or placebo)
- Reconcile returned study supplements
- Dietitian review, collection of diet diary and dispensing of new diary
- Review and record changes to concomitant medications and dose
- AE monitoring

6.2.6 Treatment Visit (Visit 7) (Month 5)

At Visit 7 (140 days after the Baseline visit +/-3), the following activities will occur:

- Body weight
- Waist circumference
- Dispensing of 1 month supply of supplement (active or placebo)
- Reconcile returned study supplements
- Dietitian review, collection of diet diary and dispensing of new diary
- Review and record changes to concomitant medications and dose
- AE monitoring
- Dispense Stool Collection Kit (All Participants – Main Study)
- Cognitive function tests (substudy)

Participants will also be given an information sheet and consent form about the weight loss maintenance trial.

6.2.7 Follow-Up Visit (Visit 8) (Month 6)

Participants will no longer be required to take the investigational supplements. Participants will be randomised to one of three groups at the start of the follow-up period. Randomisation will be stratified by the amount of weight loss at 6 months (>5% versus <5%). The behavioural techniques of each group are described fully in Table 5.

Group 1: Usual care

Participants will receive generic encouragement to continue with their weight loss behaviours learned during the weight loss programme.

Group 2: self-weighing

Participants will receive the usual care intervention but will also be asked to weigh themselves daily and record it on a record card. (The differences between groups two and three are that they will not receive scales (that return weights) or have accountability i.e. they will not receive additional emails or phone calls if weight changes, or if they do not weigh themselves. (If participants do not have scales they will be provided with some).

Group 3: Self-weighing plus accountability

Participants will be given a set of weighing scales and be asked to weigh daily. These weighing scales send the weight to an online programme that participants can access to examine their progress (www.bodytracecom). The investigators will also be able to access each participant's data. If participants do not weigh themselves for 7 days, or their weight increases by 2 kg in 7 days, participants will be emailed to remind them to weigh themselves regularly, or if they have gained weight to remind them to restart the use of the behaviours they learned during their 6-month weight loss consultations.

A sample of participants in group three will be invited to take part in the sub study investigating the psychological and behavioural processes participants adopt when weighing themselves daily. Participants will download a Smartphone voice recording memo application (Transcribeme) and will be asked to 'talk out loud' about their thoughts, feelings and behaviours they adopt after stepping on the scale. This data will be collected at month nine. At Visit 8 (168 days after the Baseline visit +/-7), the activities will occur:

- Urine collection (female of child bearing potential)
- Body weight
- Waist circumference
- Blood pressure and heart rate
- Blood collection
- Handgrip strength test
- Dispense Stool Collection Kit (Microbiota Dynamics Cohort)
- Collect stool specimens (Main Study)
- Dual-energy X-ray absorptiometry (DXA) scan
- Reconcile returned study supplements
- Dietitian review, collection of diet diary

- Completion of questionnaires
- Review and record changes to concomitant medications and dose
- AE monitoring
- Randomisation to weight loss maintenance (one of three groups)

The dental staff will carry out an oral examination on all participants who have consented to be in the sub-study. The participants will be asked to complete a questionnaire (Appendix S) regarding their oral habits/perceptions and to undergo an oral examination of all their teeth. Name, age, gender and postcode (rural/urban dweller) and the number of decayed, missing and filled teeth (DMFT) will be recorded on a chart (Appendix T). The health of the participant's gums will be assessed using the computerised charting on the Florida Probe System. Supra-gingival plaque samples will be collected, kept at -80°C and analysed for microbiological identification.

6.2.8 Follow-Up Visit (Visit 9) (Month 7)

At Visit 9 (196 days after the Baseline visit +/-7), the following activities will occur:

Microbiota Dynamics Cohort

- Collect stool specimens (Microbiota Dynamics Cohort)
- Dispense Stool Collection Kit (Microbiota Dynamics Cohort)

6.2.9 Follow-Up Visit (Visit 10) (Month 8)

At Visit 10 (224 days after the Baseline visit +/-7), the following activities will occur:

- Collect stool specimens (Microbiota Dynamics Cohort)

6.2.10 Follow-Up Visit (Visit 11) (Month 9)

At Visit 11 (273 days after the Baseline visit +/-7), the following activities will occur:

- Body weight
- Waist circumference
- Blood pressure and heart rate
- Dispense Stool Collection Kit (All Participants - Main Study)
- Dietitian review, collection of diet diary and dispensing of new diary
- Review and record changes to concomitant medications and dose
- Collection of weight record card from the self-weighing intervention group

- AE monitoring

6.2.11 Final Visit (Visit 12) (Month 12)

At Visit 12 (357 days after the Baseline visit +/-7), the following activities will occur:

- Urine collection (female of child bearing potential)
- Body weight
- Waist circumference
- Blood pressure and heart rate
- Blood collection
- Handgrip strength test
- Dual-energy X-ray absorptiometry (DXA) scan
- Cognitive function tests (substudy)
- Collect stool specimens (All Participants - Main Study)
- Dietitian review and collection of diet diaries
- Collection of weight record card from the self-weighing intervention group
- Completion of questionnaires
- Review and record changes to concomitant medications and dose
- AE monitoring

6.2.12 Termination Visit

The Termination visit is the same as visit 12, and can be conducted at any time during the study after the Baseline visit:

- Body weight
- Waist circumference
- Urine collection (female of child bearing potential)
- Blood pressure and heart rate
- Blood collection
- Handgrip strength test
- Dual-energy X-ray absorptiometry (DXA) scan
- Cognitive function tests (substudy)
- Collect stool specimens (All Participants - Main Study)
- Dietitian review and collection of diet diaries
- Completion of questionnaires

- Review and record changes to concomitant medications and dose
- AE monitoring

6.3 Efficacy Assessments

6.3.1 SF-36 Health Survey

The Quality of Life questionnaire to assess vitality and other components of quality of life will be completed by Participants at Baseline, Month 6, and Month 12. See Appendix B.

6.3.2 Assessment of Quality of Life-8D (AQOL-8D) Questionnaire

The AQOL-8D assesses quality of life with reference to independent living, happiness, mental health, coping, relationships, self-worth, pain and senses. It will be completed by Participants at Baseline, Month 6, and Month 12. See Appendix C.

6.3.3 Patients' Global Impression of Change (PGIC) Scale

The PGIC Scale asks the Participant to evaluate the effectiveness of treatment. It will be completed by Participants at Baseline, Month 6, and Month 12. See Appendix D.

6.3.4 Pain Rating Scales

The Pain Rating Scale assesses the severity and quality of pain experienced by the Participant. It will be completed at Baseline, Month 6, and Month 12. See Appendix E.

6.3.5 Weight Locus of Control (WLOC) Questionnaire

The WLOC questionnaire assesses the Participants' perception of the degree of control that they have with regards to their weight. It will be completed by Participants at Baseline, Month 6, and Month 12. See Appendix F.

6.3.6 Target Weight (Goal Weight)

Participants will be asked to specify whether they have a target weight (or goal weight) for the present weight loss attempt. If yes, participants will be asked to indicate their target weight (or goal weight) in 6 months time. It will be completed at Baseline, Month 6 and Month 12. See Appendix G.

6.3.7 Goal Commitment Scale

This scale assesses Participants commitment to their weight loss goals. It will be completed at Baseline, Month 6 and Month 12. See Appendix H.

6.3.8 Effort Scale

This scale assesses Participants perceived effort to be and invested into achieving their weight loss goal. It will be completed at Baseline, Month 6 and Month 12. See Appendix I.

6.3.9 Expected Weight

Participants will be asked to indicate their expected weight (in kg) in 6 months time. It will be completed by Participants at Baseline, Month 6 and Month 12. See Appendix J.

6.3.10 General Self-Efficacy Scale

This scale assesses optimistic self-beliefs relating to different difficult demands throughout life. It will be completed by Participants at Baseline, Month 6 and Month 12. See Appendix K.

6.3.11 Five Factor Model and Vignettes

This scale assesses personality at the broadest level. Especially conscientiousness and openness to experience are linked to health behaviours, understanding of tasks and the interest to learn new tasks. To be able to control for the scaling bias in personality assessment, each participant is asked to rate the personality of three hypothetical persons. It will be completed by Participants at Baseline, Month 6 and Month 12. See Appendix L.

6.3.12 Risk and Time Preferences

Individual willingness to take risks and willingness to wait for delayed gratification (labelled time preference by economists) are thought to be the key variables explaining individual behaviour in the economics discipline. Increased willingness to wait for delayed gratification has been associated with lower BMI (Ikeda et al. 2010, Smith et al. 2005) and risk preferences have been shown to be affected by thirst and hunger (Yamada, 2013). We propose to use standard tasks from economics developed by Holt and Laury (2002) and Andreoni and Sprenger (2012) to measure risk and time preferences in obese participants to verify whether the intervention affects these decision-making fundamentals that have been previously identified to correlate with being overweight. It will be completed by Participants at Baseline, Month 6 and Month 12. See Appendix M.

6.3.13 Outcome-Expectancies Scale

This scale assesses Participants expectancies regarding the outcomes of losing weight (e.g., "I will feel physically more attractive"). It will be completed by Participants at Baseline, Month 6 and Month 12. See Appendix N.

6.3.14 Dissatisfaction Scale

This single-item question asks Participants to what extent they feel dissatisfied with their current weight loss progress. It will be completed by Participants at Month 6 and Month 12. See Appendix O.

6.3.15 Economics of Obesity

This questionnaire assesses whether weight loss is followed by improvements in employment and wages or salary. It will be completed by Participants at Baseline, Month 6 and Month 12. See Appendix P.

6.3.16 Dietary Intake

All Participants will be required to keep 3 day diet diaries (2 working days and 1 week-end day) at Baseline (day 0), Month 1, 2, 3, 4, 5, 6, 9, and 12 as a measure of dietary compliance. See Appendix Q.

6.3.17 Accelerometer

An accelerometer pedometer will be provided to each Participant at the Screening visit for the study duration. Participants will be able to keep the accelerometer pedometer at the end of the study. This will act as a motivational tool to increase their activity level. Participants will be asked to wear the device for 5 days prior to Baseline (day 0), Month 1, 2, 3, 4, 5, 6, 9, and 12.

6.3.18 Medication Compliance

Measurement of medication non-adherence is crucial to identify Participants at risk for poor outcomes. During the treatment period for each arm of the trial, the study site will complete an accountability log for each Participant recording the date, the visit, and amount of supplements dispensed, as well as the date, the visit, and amount of supplements returned.

A Participant whose compliance is less than 80% on three occasions will not be withdrawn from the study, but will be treated as a non-complier. If a Participant wishes to withdraw from the

study an Investigator will actively attempt to continue collecting data from them if the Participant consents to do so.

6.3.19 Stool Collection Protocol

6.3.19.1 Sampling Schedule

Participants will be provided with stool specimen collection kits at the Screening visit, Month 5, and 9 as in Table 6.3.19.1. The Participants will be asked to collect a sample, record the date, and immediately freeze their stool specimen at their home. Frozen stool samples are to be delivered to the clinic at Baseline, Month 6 – which is the end of intervention period, and Month 12 - end of follow up period.

Participants in the Microbiota Dynamics Cohort will be given 4 stool specimen collection kits at Baseline, Month 1, 6, and 7. These Participants will be asked to collect a stool specimen once a week over an 8 week period at the start and end of the intervention period. The frozen stool specimens are to be returned to the clinic at Month 1, 2, 7, and 8. See Table 6.3.19.1.

Table 6.3.19.1 Schedule for Microbiota Sampling and main study collection

Visit Number	1	2	3	4	7	8	9	10	11	12
Month	0	0	1	2	5	6	7	8	9	12
**Main Study – all Participants**										
Participants receive collection kit	✓				✓				✓	
Return frozen specimen to clinic		✓				✓				✓
**Microbiota Dynamics Cohort – subsample of Participants**										
Participants receive collection kit		✓	✓			✓	✓			
Return frozen specimen to clinic			✓	✓			✓	✓		

6.3.19.2 Specimen Labelling

At the time of provision of the stool specimen collection kit, a pre-printed label should be placed on the exterior wall of the container. Site staff will write the Participant ID Number, Visit Number, and the Treatment Group Number (1, 2, 3, or 4) on each container. For example, the label should bear the following information (Figure 6.3.19.2) at the time of the return:

Figure 6.3.19.2 Specimen Labelling

Participant ID	: XXXXXX
Date received	: 16/JUN/2014 (dd/mmm/yyyy)
Month	: 12
Treatment group	: C

6.3.19.3 Materials Needed

One unit of stool specimen collection kit will contain:

- 1 sterile stool container with pre-printed label
- 1 specimen collection bag
- 1 pair of latex gloves
- Specimen collection instructions

6.3.19.4 Collection Method

The following information and the kit for stool specimen collection will be provided to the Participants at the designated visits (Appendix A). Upon delivery of the specimen to the clinic, the containers will be inspected for integrity and cleanliness. The site staff should make sure that the Participant ID Number, Visit Number, and the Treatment Group Number (A, B, C, D) have been recorded. Stool specimens should be immediately stored in -20^{0}C freezer at the clinic. The stool sample collection log will be completed by the site staff.

6.3.20 Practitioners intuition

After the baseline appointment the dietitian will write down how much weight they expect the participant to lose and their reasons why they have come to this conclusion.

6.4 Study Restrictions

6.4.1 Dietary

The Participant will meet the study Dietitian for advice on their individual hypo-caloric diet for weight loss as well as when and how often to take the supplements (placebo or active). The study Dietitian will discuss the food diary at the Baseline visit and each subsequent visit (except for visit 9 and 10 where there is no contact with the study Dietitian). The study

Dietitian will review the behavioural modification programme with each of the study Participants and the importance of compliance throughout the 12 month period of the study, which will be monitored by the Dietitian during face-to face meetings at monthly clinic visits. This approach is based on the Type 2 Diabetes Lifestyle Intervention Program and focuses on a healthy, low calorie, low fat diet, with exercise of moderate intensity, such as brisk walking.

The specific goals for this programme are as follows:

- >5% weight reduction with an ideal weight reduction of ≥10%
- Total fat intake of ≤ 30% of total energy
- Saturated fat < 10% of total energy
- Fibre intake of ≥ 15g/1000 kcal
- Moderate exercise ≥ 30 min/day
- Diet high in whole grains, fruit & vegetables

The study Dietitian will provide a written guide as to the specific types of foods and the quantities which can be consumed, in addition to an exercise regimen, which will assist in the compliance to this programme. This guide will be personalised by the Dietitian for each Participant, in consultation with the Participant, based on their specific preferences, nutritional requirements, medical status and dietary and exercise goals. This guide will be reviewed at the monthly clinic visits and adjusted as required, based on changes in weight and exercise tolerance, relative to their previous clinic visits (Appendix Q).

6.4.2 Smoking and Alcohol Intake

Advice will also be given on other healthy lifestyle measures including alcohol intake, and smoking.

6.4.3 Confinement

Pregnant or breastfeeding women and women who may be planning a pregnancy are not eligible. A urine pregnancy test will be conducted on women of child bearing potential at the Screening visit. A positive test at Screening would exclude the female from the study. A positive result during or at the end of the study is regarded as a Serious Adverse Event (SAE).

The Investigator must collect pregnancy information for female trial Participants or female partners of male trial Participants. This includes Participants who become pregnant while

during a stage where the foetus could have been exposed to the investigational medicinal product. Any pregnancy should be reported to the Sponsor using the SAE form and the Participant should cease taking the supplements. The pregnancy should be followed up by the Investigator until delivery and reported to the Sponsor.

6.4.4 Concomitant Medication

Concomitant medications will be recorded at each visit. During the initial six months of the study, Participants will be encouraged not to change their medication usage to determine the true effect of the study interventions.

6.5 Safety Assessments

6.5.1 Physical Examination

Physical examination will include height, body weight, BMI, waist circumference, heart rate and blood pressure:

Height

Height will be measured at the Screening visit using a wall mounted stadiometer accurate to 0.5 cm.

The Participant will be in the standing position, shoes removed, with their heels, buttocks and shoulder blades in contact with the vertical surface of the stadiometer. The moveable headboard will be lowered gently until it touches the crown of the head. Height will be recorded in centimetres to the nearest centimetre.

Body Weight

At each visit body weight will be measured on calibrated scales shortly after the Participant empties their bladder. The Participant is to be in light indoor clothing with pockets empty and without shoes, belts, jewellery, or other accessories. Body weight will be recorded in kilograms to the nearest 0.1 kilogram and measured at every clinic visit.

Body Mass Index (BMI)

BMI will be recorded from the Participant's recorded weight and height using the following equation:

$$\frac{\text{weight (kg)}}{\text{height (m)}^2}$$

This will be calculated to the nearest one-tenth unit to determine their eligibility for the trial at Screening.

Waist Circumference

Waist circumference will be measured with a flexible tape measure with the Participant wearing light clothing, at the mid-point between the highest point of the iliac crest and lowest part of the costal margin in the mid-axillary line in expiration. Measurements will be collected at each clinic visits and recorded in centimeters to the nearest 0.5 centimeter.

Blood Pressure

Systolic blood pressure and diastolic blood pressure will be measured every three months using a digital sphygmomanometer. The procedure for measurements is:

1. A quiet room with minimal extraneous activity and temperature fluctuation is to be used.
2. Participant should empty his/her bladder prior to blood pressure measurements.
3. Participant should be seated in a chair with back supported and arms bared and supported at heart level.
4. Measurement should begin after at least 5 minutes of rest.
5. The appropriate cuff size must be used to ensure accurate measurements. The upper arm should be wholly encircled by at least 80% of the total length of the cuff. The appropriate cuff is to be applied around the upper arm so that the midpoint of the length of the cuff lies over the brachial artery and mid-height of the cuff is at heart level. The lower edge of the cuff should be across the natural crease of the inner aspect of the elbow.
6. Blood pressure will be measured in the same arm each time except at the Screening visit when the blood pressure will be measured in both arms. A second reading will then be taken in the arm with the highest reading. If a difference of > 10 mmHg is found between the first and second reading in systolic or diastolic blood pressure, a third reading will be taken.
7. Blood pressure will be measured twice, the measurements separated by at least two minutes.
8. Both systolic and diastolic blood pressure will be recorded, as well as heart rate using the same digital sphygmomanometer. The mean of the measurements will be used as the basis of evaluation.

Heart Rate

Heart rate will be measured every three months, concurrently with blood pressure, using the same digital sphygmomanometer.

BMI

BMI values for the cut-off points for classification of overweight and obesity vary within different countries. According to the World Health Organization (WHO) Guidelines, a BMI of 18.5 to 24.9 kg/m^2 is normal, 25 to 29.9 kg/m^2 is classified as overweight or pre-obese, over 30 kg/m^2 is classified as obese, which includes obese class I (30 to 34.9 kg/m^2), obese class II (35 to 39.9 kg/m^2) and obese class III (> 40.0 kg/m^2). A BMI of less than 18.5 kg/m^2 is classed as underweight (Table 6.5.1). This classification is based on standards for adults of European descent.

Table 6.5.1 BMI Classification

Classification BMI	(kg/m^2)
Underweight	< 18.5
Normal	18.5 – 24.9
Overweight	25.0 – 29.9
Obese class I	30.0 – 34.9
Obese class II	35.0 – 39.9
Obese class III	> 40.0

6.5.2 12-Lead ECG

A 12-lead resting ECG will be obtained at the Screening visit to check for any recent onset of cardiovascular disease that is not known. The ECG will be read prior to the Baseline visit, signed and dated by a suitably qualified person at the site.

6.5.3 Laboratory Safety Testing

Fasting blood samples will be collected from all Participants at Screening, Month 3, 6 and 12. Please refer to Table 6.5.3.1 and Table 6.5.3.2 for an exact description of the sample collected at each visit. Blood samples will be sent to the laboratory for commercial analysis. A small amount of blood plasma (approximately 10 ml) will also be stored in cryotubes in a -80°C freezer and used at a later date for circulating hormones or factors that influence muscle mass or strength, gut hormone and adipokine analysis, and SCFA measurement. These analyses will be performed at the University of Sydney.

Table 6.5.3.1 Laboratory tests conducted at each visit

VISIT	1	2	5	8	12
**Month**	**0**	**0**	**3**	**6**	**12**
**Day**	-7 (+/-7)	0	84 (+/-7)	168 (+/-7)	357 (+/-7)
Fasting glucose	✓		✓	✓	✓
HbA1c	✓		✓	✓	✓
Full lipid profile	✓		✓	✓	✓
Plasma SCFA	✓		✓	✓	✓
Liver enzymes	✓			✓	✓
hsCRP	✓			✓	✓
Apolipoprotein B	✓			✓	✓
TSH*	✓				
Creatinine and eGFR*	✓				
Full blood count*	✓				
Circulating hormones/factors e.g. IGF-1, IGF-1 binding proteins, cortisol	✓		✓	✓	✓
Adipokines & Gut hormones	✓			✓	✓
Faecal microbiota community profile		✓		✓	✓
Faecal SCFA		✓		✓	✓
Supra-gingival plaque		✓		✓	

*For those who do not have results within 6 months of the Screening visit

Table 6.5.3.2 Laboratory tests conducted for the Microbiota Dynamics Cohort**

Visit	1	2	3	4	5	8	9	10	12
**Month**	**0**	**0**	**1**	**2**	**3**	**6**	**7**	**8**	**12**
**Day**	-7 (+/-7)	0	28(+/-7)	56(+/-7)	84(+/-7)	168(+/-7)	196(+/-7)	224(+/-7)	357(+/-7)
Faecal microbiota community profile		✓	✓	✓		✓	✓	✓	✓
Faecal SCFA		✓	✓	✓		✓	✓	✓	✓
Plasma SCFA	✓				✓			✓	✓

**40 Participants (10 Participants from each treatment group)

6.5.3.1 Biochemistry

Thyroid Stimulating Hormone (TSH), Creatinine and eGFR (for those who do not have results within 6 months of Screening) will be performed at the Screening visit.

6.5.3.2 Haematology

A Full blood count (for those who do not have results within 6 months of Screening) will be performed at the Screening visit.

6.5.3.3 Study Specific tests

Fasting glucose, HbA1c, Full lipid profile, will be collected at Screening, Month 3, 6 and 12. Fasting glucose, HbA1c, Full lipid profile, Liver enzymes, High-sensitivity C-reactive protein (hsCRP), Apolipoprotein B will be collected at Screening, Month 6 and 12. Routine Stool samples will be collected from all Participants at Baseline, Month 6 and 12.

About 40 Participants (10 from each treatment group) will have an additional weekly stool collection for Microbiota Dynamics assessment over two 8-week periods the first from Month 2 to 4, the second from Month 8 to 10. The Microbiota Dynamics assessment Participants will collect twice weekly and will deliver 8 stool specimens, in visits scheduled for Month 1, 2, 7 and 8. Please see Appendix A for instructions.

6.5.3.4 Handgrip Strength test

A handgrip strength test will be performed at Screening, Month 3, 6 and 12 to monitor muscle strength changes throughout the study. The Participant will be required to sit comfortably on a chair that has back support and fixed arms. The Jamar Hydraulic Hand Dynamometer will be used in this test and will take approximately 6 minutes to complete.

6.5.3.5 Dual-energy X-ray absorptiometry (DXA) scan

A dual-energy x-ray absorptiometry (DXA) scan will be performed at Baseline, Month 6 and 12 for analysis of total body fat and fat free mass. The Participant will be required to lie on a padded table. The DXA scan takes approximately 20 minutes to complete. Some Participants may find the special bed a little uncomfortable to lie on. They will be exposed to a tiny amount of X-ray radiation during the DXA scan. Current literature suggests this range is from 0.08-4.6 microsieverts. However, for most machines the dose is of the order of 1 microsievert (1uSv), which is less than everyone receives from natural 'background' radiation each day. The risk is negligible.

6.5.3.6 Cognitive Function

One hundred participants with a BMI ≥ 30 kg/m^2 will be required to undergo a cognitive assessment at Screening, Month 5 and Month 12. The CANTAB system (Cambridge Neuropsychological Test Automated Battery) will be used in this study. The CANTAB console contains a suite of touch screen based automated tests specifically designed to assess cognitive function across a range of domains, including memory, executive function, attention, decision-making and social cognition. The CANTAB system accelerates and facilitates the administration of cognitive function tests by non-specialist researchers. It reduces data errors and is significantly more sensitive at detecting changes in cognitive function than traditional paper tests. This system tested for validity is now used on an international stage. Since this very little has been performed in this field of research, this study will serve as a pilot and hence specific power calculations are unknown. However, a recent trial (sample size = 80) of cognitive remediation therapy in adults with obesity found large effect sizes in cognitive flexibility outcomes in set shifting tasks (Raman, 2016 manuscript in preparation), justifying a sample size of 100 participants.

The specific CANTAB tests to be administered will be the reaction time (RTI), attention switching task (AST), and the stop signal test (SST) as these tests have been shown to be the most sensitive for detecting differences in cognitive function (specifically measuring impulsive action and cognitive flexibility) in cross-sectional studies of normal weight individuals and those with obesity (Grant, 2014; Chamberlain, 2015). An intelligence quotient (IQ) test (National Adult Reading Test-revised (NART-R)) will also be administered to participants at the first visit to allow for comparison to population norms, as well as the Patient Health Questionnaire (PHQ-9) questionnaire to screen out those participants with major psychological disorders. The Kessler Psychological Distress Scale (K10) will be administered along with the specific CANTAB tests at screening, month 5 and month 12. The K10 will assess change in psychological distress throughout the study. Total testing time at each visit will be maximum of 30 minutes.

6.5.3.7 Urinalysis

A urine sample will be collected from women of child bearing potential at Screening, Month 3, 6 and 12 and tested for human chorionic gonadotropin (HCG). Participants with a positive result will be excluded from the study. Please refer to section 6.4.3 for Participants who become pregnant during the study.

6.5.3.8 Faecal Analysis

Stool samples will be collected at Baseline, Month 6 and 12 for faecal microbiota community profile, faecal Short-Chain Fatty Acids (SCFA) and plasma SCFA. Please refer to Table 6.5.3.2 for an exact description of when the samples are collected. All analyses will be performed at the University of Sydney. SCFA concentration in the stool will be measured. DNA profile of the gut microbiota community will be obtained with high throughput sequencing (454 pyrosequencing or Illumina MiSeq) of the 16s rRNA gene.

6.5.3.9 Microbiota Dynamics Assessment

Forty Participants will provide additional stool samples for Microbiota Dynamics. Additional Faecal SCFA and Faecal microbiota stools will be collected at visits scheduled for Months 1, 2, 7 and 8. Plasma SCFA will be collected at Baseline, Months 3, 6 and 12 visits. Please refer to Table 6.5.3.2 for an exact description of when the samples are collected. All analyses will be performed at the University of Sydney.

6.5.3.10 Supra-gingival Plaque Analysis

The samples will be immediately placed in separate microtubes containing 0.15 ml of TE (10 mM Tris-HCl, 1 mM EDTA, pH 7.6) and 100 µl of 0.5 M NaOH will be added to each tube. Subsequently, the samples will be evaluated for their content of yellow, red and green complex bacterial species by the 454 sequencing technique. The 454 sequencing technique allows comprehensive analysis of composition of bacterial communities at the level of species.

6.5.4 Adverse Events (AE)

The Investigator and designated study personnel will monitor each Participant for AE during the study. All AEs reported between consent and final follow- up will be recorded in the source documents and in the Electronic Case Report Form (eCRF). The Investigator or designee will ask the Participant non-leading questions in an effort to detect AEs. Examples of this are:

> "How are you feeling?"
> Or
> "Since you were last asked, have you felt unwell or different from usual?"

In addition, Participants should be encouraged to spontaneously report any unusual feelings or sensations. See Section 8 for full details on adverse experience reporting.

7 Investigational product(s) (IP)

7.1 Description of Investigational Products (IP)

Presentation:

- FBCx: White oblong tablet containing 1000mg of Alpha Cyclodextrin.
- Placebo: White oblong tablet containing inactive compound
- Ginst15: dark brown soft capsule which contains 160 mgs of Ginst15
- Placebo: dark brown soft capsule containing inactive compound

During the treatment phase for 168 +/-7 days (24 weeks) Participants will take a total of 12 pills per day (6 tablets and 6 capsules). Participants will receive a 1 month supply of supplements. Neither the Participants nor the study staff will know to which tablets or capsules each Participant is randomised, therefore each Participant takes exactly the same number of capsules and tablets. Each Participant will take 2 capsules prior to each meal and 2 tablets 20 minutes after each meal, three times per day.

Each compound will be packaged as follows:

- **FBCx tablet** = 210 tablets per month – 1 jar x 210 tablets for 1 month supply.
- **Placebo tablet** = 210 tablets per month – 1 jar x 210 tablets for 1 month supply.
- **Ginst15 capsule** = 210 capsules per month – 1 jar x 210 capsules for 1 month supply.
- **Placebo capsule** = 210 capsules per month – 1 jar x 210 capsules for 1 month.

7.2 Dose Justification

Participants will be given instructions on when to take the supplements and prescribed their individualised diet for weight loss. Each diet will be energy and macronutrient matched with a 500 kilocalorie per day deficit based on Harris Benedict equation.

Participants will receive a 1 month supply of supplements during the treatment phase. To maintain the double blind, all Participants from each group will take 2 Ginst15 or placebo before meals, and 2 FBCx or placebo after meals.

All Participants will be asked to return their IP and study product containers (SPC) for reconciliation during the treatment period.

The study Dietitian will provide a written guide as to the specific types of foods and the quantities that can be bought and consumed. Participants will be required to keep a 3 day diet diary every 4 weeks (2 working days and 1 weekend day). See Appendix Q.

7.3 Comparator Justification

Based upon the properties of FBCx and Ginst15, the current double blinded, randomised controlled trial is designed to investigate the beneficial effects of these products in overweight and obese Participants with pre diabetes. It is proposed that these products may over-lap in their function and therefore are being compared in the same trial in the prevention of type 2 diabetes.

7.4 Administration

During the treatment phase for 168 +/-7 days (24 weeks) (6 months) Participants will take a total of 12 pills per day (6 tablets and 6 capsules). Participants will receive a 1 month supply of IP (as described in Figure 7.4). Neither the Participants nor the study staff will know to which tablets or capsules each Participant is randomised, therefore each Participant takes exactly the same amount of capsules and tablets.

FBCx group:

Participants in the FBCx group will be asked to take 2 placebo capsules prior to each meal, and 2 x 1 gram FBCx tablets directly (within 20 minutes) after each meal 3 times per day. Participants will take a total of 12 pills per day (6 tablets and 6 capsules) for 168 +/-7 days (24 weeks) (6 months).

Ginst15 group:

Participants in the Ginst15 group will be asked to take 2 x 160 mg Ginst15 capsules prior to each meal, and 2 placebo tablets directly (within 20 minutes) after each meal 3 times per day. Participants will take a total of 12 pills per day (6 tablets and 6 capsules) for 168 +/-7 days (24 weeks) (6 months).

Combined therapy group:

Participants in the combined therapy group will take 2 x 160 mg of Ginst15 capsules prior to each meal and 2 x 1 gram FBCx tablets directly (within 20 minutes) after each meal 3 times per day. Participants will take a total of 12 pills per day (6 tablets and 6 capsules) for 168 +/-7 days (24 weeks) (6 months).

Behavioural Management Programme:

Participants in the standard behavioural management programme alone will be required to take 2 placebo capsules prior to each meal, and 2 placebo tablets directly (within 20 minutes) after each meal 3 times per day. Participants will take a total of 12 pills per day (6 tablets and 6 capsules) for 168 +/-7 days (24 weeks) (6 months).

Figure 7.4 Four arms of the trial

**FBCx**	6x 1 placebo Capsules 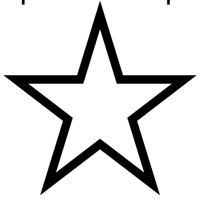	6 x 1 gram FBCx tablets 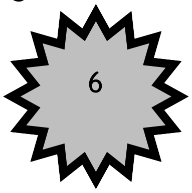
**Ginst15**	6 x 1 GINST Capsules 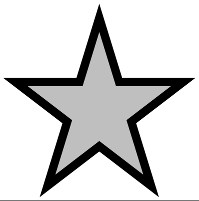	6 x 1 placebo tablets 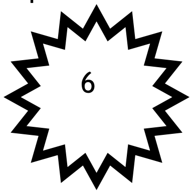
**FBCx + Ginst15 combined**	6x 1 GINST Capsules 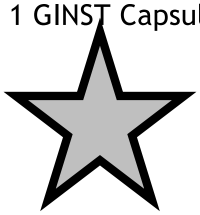	6 x 1 gram FBCx tablets 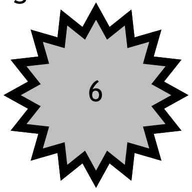
**Placebo**	6 x 1 placebo Capsules 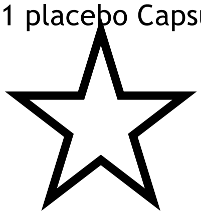	6 x 1 placebo tablets 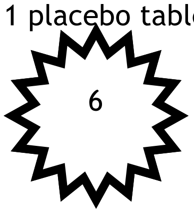

7.5 Randomisation

All treatment groups will include a standard behavioural management programme (nutrition, exercise, and behavioural advice for weight loss) and be allocated to one of four treatment groups (FBCx; Ginst15; FBCx + Ginst15 combined; or placebo using a randomisation programme (FileMaker Pro).

Randomisation will be based on a simple block randomisation.

The Investigators (except for the data manager (Associate Professor Gareth Denyer), Participants and Sponsor will be blinded as to their treatment allocation. All containers will look identical between the groups other than the additional information of A, B, C or D.

At the commencement of the weight loss maintenance period (month 6), Participants will be randomised to one of three follow-up weight maintenance groups. Random group allocation will be by initial intervention arm and stratified by weight loss during Baseline to 6 months.

7.6 Unblinding

A treatment code break should only be requested in a medical emergency where the identity of the study treatment is essential for the medical management of the Participant.

When requesting a code break, do not reveal the study treatment to any of the study team, including CRA, Principal Investigator (PI) or pharmacist.

To break the code:

1. Contact the data manager (Associate Professor Gareth Denyer) and request computer treatment code break.
2. Document the reason for unblinding the Participant in the source notes.
3. Restrict as much as possible the number of people informed of the identity of the study treatment (site and Sponsor level).

7.7 Product Labelling

Each SPC will have 2 perforated labels. One label will stay attached to the SPC while the other half of the label will be detached and placed into the Participant's folder.

The labels contain:

- Study name
- Participant number and initials
- Directions for use
- Storage Instructions
- Bottle code
- Date Dispensed
- Visit number
- Date and Quantity Returned
- Site contact details

- For “clinical trial use only”
- Keep out of reach of children

7.8 Handling and Storage of Study Drugs

All study products are to be stored at room temperature. The study site will confirm receipt of the IP/Placebo in SPC to the study site. The study site will check the shipment and confirm the SPC is intact, not damaged, and can be reconciled with the inventory. Any discrepancies must be acted upon immediately.

The study site must maintain inventory records of the SPC. Records must be made available to the Sponsor and to representatives of regulatory and other governmental bodies, as required by law, regulation or contractual agreement.

The study site must complete an accountability log (medication compliance) for each Participant for both the FBCx/Placebo and the Ginst15/Placebo in each arm - recording the date, the visit, and amount dispensed, as well as the date, the visit and amount returned. The accountability log (medication compliance) must be available for inspection at any time. A copy of the dispensing log will be given to the Sponsor at the end of the study.

At the conclusion of the study, the Investigator and the Sponsor will reconcile all used and unused supplements. The inventory record for returned supplements will be completed. Unused supplements will be destroyed on site following agreement from the Investigator, and a representative of the Sponsor. The Sponsor will keep the original certificate of destruction and copies of this document will be sent to the Investigator.

7.9 Dispensing of Study Drugs

Supplements will be dispensed from the Clinical Trials Unit to the Participants and all dispensing will be recorded in the Clinical Trials Unit IP dispensing log and in the Participant’s eCRF. A product accountability log will be utilized to track all IP to the Clinical Trials Unit.

8 Adverse Events (AE) and Serious Adverse Events (SAE)

The Investigator is responsible for the detection and documentation of events meeting the criteria and definition of an AE or a SAE as provided in this protocol. During the study, when there is a safety evaluation, the Investigator or site staff will be responsible for detecting AEs and SAEs, as detailed in this section of the protocol.

8.1 Definition of an Adverse Event

Any untoward medical occurrence in a Participant temporarily associated with the use of a medicinal product, whether or not considered related to the medicinal product.

An AE can therefore be any unfavourable and unintended sign (including an abnormal laboratory finding), symptom, or disease (new or exacerbated) temporally associated with the use of a medicinal product, whether or not considered related to the medicinal product. For marketed medicinal products, this also includes failure to produce benefits (i.e. lack of efficacy), abuse or misuse.

Examples of an AE include:

- Exacerbation of a chronic or intermittent pre-existing condition including either an increase in frequency and/or intensity of the condition.
- New conditions detected or diagnosed after IP administration even though it may have been present prior to the start of the study.
- Signs, symptoms, or the clinical sequelae of a suspected interaction.
- Signs, symptoms, or the clinical sequelae of a suspected overdose of either IP or a concurrent medication (overdose per se should not be reported as an AE/SAE).

Examples of an AE **do not include** a/an:

- Medical or surgical procedure (e.g. endoscopy, appendectomy); the condition that leads to the procedure is an AE.
- Situations where an untoward medical occurrence did not occur (social and/or convenience admission to hospital).

In this study, AEs may include pre- or post-treatment events that occur as a result of protocol-mandated procedures (i.e. invasive procedures, modification of Participants' previous therapeutic regimen).

8.2 Definition of a Serious Adverse Event (SAE)

A serious adverse event is any untoward medical occurrence that, at any dose:

a) results in death

b) is life threatening

Note: The term 'life-threatening' in the definition of 'serious' refers to an event in which the Participant was at risk of death at the time of the event. It does not refer to an event, which hypothetically might have caused death if it were more severe.

c) requires hospitalisation or prolongation of an existing hospitalisation

Note: In general, hospitalisation signifies that the Participant has been detained (usually involving at least an overnight stay) at the hospital or emergency ward for observation and/or treatment that would not have been appropriate in the physician's office or out-patient setting. Complications that occur during hospitalisation are AEs. If a complication prolongs hospitalisation or fulfils any other serious criteria, the event is serious. When in doubt as to whether 'hospitalisation' occurred or was necessary, the AE should be considered serious.

Hospitalisation for elective treatment of a pre-existing condition that did not worsen from Baseline is not considered an AE.

d) results in disability/incapacity, or

Note: The term disability means a substantial disruption of a person's ability to conduct normal life functions. This definition is not intended to include experiences of relatively minor medical significance such as uncomplicated headache, nausea, vomiting, diarrhoea, influenza, and accidental trauma (e.g. sprained ankle) which may interfere or prevent everyday life functions, but do not constitute a substantial disruption.

e) is a congenital abnormality/birth defect.

Medical and scientific judgement should be exercised in deciding whether reporting is appropriate in other situations, such as important medical events that may not be immediately life-threatening or result in death or hospitalisation, but may jeopardise the Participant, or may require medical or surgical intervention to prevent one of the other outcomes listed in the above definition. These should also be considered serious. Examples of such events are invasive or malignant cancers, intensive treatment in an emergency room or at home for

allergic bronchospasm, blood dyscrasias or convulsions that do not result in hospitalisation, or development of drug dependency or abuse.

8.3 Clinical Laboratory AEs, and SAEs

Abnormal laboratory findings (e.g. clinical chemistry, haematology, urinalysis) or other abnormal assessments (e.g. ECG, vital signs) that are judged by the Investigator as clinically significant will be recorded as AEs or SAEs if they meet the definition of an AE, as defined in Section 8.1, or SAE as defined in Section 8.2.

Clinically significant abnormal laboratory findings or other abnormal assessments that are detected during the study or are present at Baseline and significantly worsen following the start of the study will be reported as AEs or SAEs. However, clinically significant abnormal laboratory findings or other abnormal assessments that are associated with a disease reported in the medical history, unless judged by the Investigator as more severe than expected for the Participant's condition, or that are present or detected at the start of the study and do not worsen, will not be reported as AEs or SAEs.

The Investigator will exercise their medical and scientific judgement in deciding whether an abnormal laboratory finding or other abnormal assessment is clinically significant.

8.4 Time Period, Frequency, and Method of Detecting AEs and SAEs

All AEs will be recorded between the time of consent and the follow-up visit. Each Participant will be monitored regularly by the Investigator and study personnel for AEs occurring throughout the study. During the treatment period, the Investigator or designee will enquire about AEs by asking the following non-leading questions:

At each visit Participants will be asked:

> "How are you feeling?"

At subsequent scheduled intervals Participants will be asked:

> "Since you were last asked, have you felt unwell or different from usual?"

8.5 Recording of AEs and SAEs

When an AE/SAE occurs, it is the responsibility of the Investigator to review all documentation (e.g. hospital progress notes, laboratory, and diagnostic reports) related to the event. The Investigator will then record all relevant information regarding an AE/SAE in to the eCRF. It is

not acceptable for the Investigator to send photocopies of the Participant's medical records to the Sponsor in lieu of completion of the appropriate AE/SAE CRF pages. However, there may be instances when copies of medical records for certain cases are requested by the Sponsor. In this instance, all Participant identifiers will be removed and the study number, Participant's initials, and randomisation number will be written on the copies of the medical records prior to submission to the Sponsor.

For each AE, start and stop dates, action taken, outcome, intensity (see Section 8.8.1), and relationship to study product (causality) (see Section 8.8.2) must be documented. If an AE changes in frequency or intensity during a study, a new entry of the event must be made in the eCRF.

The Investigator will attempt to establish a diagnosis of the event based on signs, symptoms, and/or other clinical information. In the absence of a diagnosis, the individual signs/symptoms should be documented.

All details of any treatments initiated due to the AE should be recorded in the Participant's notes and the eCRF.

8.6 Prompt Reporting of SAEs

Once an Investigator becomes aware that an SAE has occurred in a study Participant, they will immediately notify the Sponsor of the event. The SAE form must be completed as thoroughly as possible with all available details of the event, signed by the Investigator (or appropriately qualified designee), and faxed to the Sponsor within 24 hours of first becoming aware of the event.

If the Investigator does not have all information regarding an SAE, they will not wait to receive additional information before notifying the Sponsor of the event and completing the form. The form will be updated when additional information is received.

The Investigator will always provide an assessment of causality at the time of the initial report as described in Section 8.8.2, "Assessment of Causality". If data obtained after reporting indicates that the assessment of causality is incorrect, then the SAE form may be appropriately amended, signed and dated, and resubmitted to the Sponsor.

In accordance with local requirements, the Investigator must also notify the Human Research Ethics Committee (HREC) who approved the study of any SAEs (according the guidelines of the HREC).

The Investigator, and others responsible for Participant care, should institute any supplementary investigations of serious adverse events based on their clinical judgement of the likely causative factors. This may include seeking further opinion from a specialist in the field of the AE. The Sponsor may also request extra tests. If a Participant dies, any post-mortem findings, including histopathology will be provided to the Sponsor when available. No medical help, diagnosis, or advice should be withheld from the Participant due to an inability to contact the Sponsor.

8.7 Expeditable Events

Expeditable events are those AEs that are **CAUSALLY** related to the study product, **AND** that are both **SERIOUS** (see Section 8.2) and **UNEXPECTED** (see Section 8.8.3). Such events are to be expedited to regulatory authorities and will be reported within the stipulated timelines by the Sponsor or a suitably qualified designee.

8.8 Evaluating AEs and SAEs

8.8.1 Assessment of Intensity

The Investigator will make an assessment of intensity for each AE and SAE reported during the study. The assessment will be based on the Investigator's clinical judgement. The intensity of each AE and SAE recorded in the eCRF should be assigned to one of the following categories:

Mild: An event that is easily tolerated by the Participant, causing minimal discomfort and not interfering with everyday activities.

Moderate: An event that is sufficiently discomforting to interfere with normal everyday activities.

Severe: An event which is incapacitating and prevents normal everyday activities.

An AE that is assessed as severe should not be confused with an SAE. Severity is a category utilised for rating the intensity of an event; and both AEs and SAEs can be assessed as severe. An event is defined as "serious" when it meets one of the pre- defined outcomes as described in Section 8.2 "Definition of an SAE".

8.8.2 Assessment of Causality

The Investigator is obligated to assess the relationship between IP and the occurrence of each AE/SAE. The Investigator will use clinical judgment to determine the relationship. Alternative

causes, such as natural history of the underlying diseases, concomitant therapy, other risk factors, and the temporal relationship of the event to the IP will be considered and investigated. The Investigator will also consult the IB and/or product information in the determination of their assessment.

The causal relationship to the study product assessed by the Investigator (or medically qualified delegate) should be assessed using the following classifications:

Not Related In the Investigator's opinion, there is not a causal relationship between the study product and the AE.

Unlikely The temporal association between the AE and study product is such that the study product is not likely to have any reasonable association with the AE.

Possible The AE could have been caused by the study Participant's clinical state or the study product.

Probable The AE follows a reasonable temporal sequence from the time of study product administration, abates upon discontinuation of the study product and cannot be reasonably explained by the known characteristics of the study Participant's clinical state.

Definitely The AE follows a reasonable temporal sequence from the time of study product administration or reappears when study product is reintroduced.

There may be situations when an SAE has occurred and the Investigator has minimal information to include in the initial report to the Sponsor. However, it is very important that the Investigator always makes an assessment of causality for every event prior to transmission of the SAE form to the Sponsor. The Investigator may change their opinion of causality in light of follow-up information, amending the SAE form accordingly. The causality assessment is one of the criteria used when determining regulatory reporting requirements.

8.8.3 Assessment of Expectedness

Expected An adverse reaction, the nature or severity of which is consistent with the applicable product information (e.g. Investigators' Brochure for an unapproved medicinal product) or product insert.

Unexpected An adverse reaction, the nature or severity of which is not consistent with information in the relevant source document (e.g. Investigators' Brochure for an unapproved medicinal product) or product insert.

8.9 Follow-up of AEs and SAEs

After the initial AE/SAE report, the Investigator is required to proactively follow up each Participant and provide further information to the Sponsor on the Participant's condition.

All AEs and SAEs documented at a previous visit/contact and are designated as ongoing, will be reviewed at subsequent visits/contacts. All AEs and SAEs will be followed until resolution, until the condition stabilises, until the event is otherwise explained, or until the Participant is lost to follow-up. Once resolved, the appropriate AE/SAE eCRF page(s) will be updated. The Investigator will ensure that follow-up includes any supplemental investigations as may be indicated to elucidate the nature and/or causality of the AE or SAE. This may include additional laboratory tests or investigations, histopathological examinations, or consultation with other health care professionals. New or updated information will be recorded on a new SAE form, with all changes signed and dated by the Investigator. The new or updated information should be resent to the Sponsor.

8.10 Post-study AEs and SAEs

A post-study AE/SAE is defined as any event that occurs outside the AE/SAE detection period as defined in Section 8.4 "Time Period, Frequency, and Method of Detecting AEs and SAEs" of the protocol.

Investigators are not obligated to actively seek AEs or SAEs in former study Participants. However, if the Investigator learns of any SAE, including death, at any time after a Participant has been discharged from the study, and he/she considers the event reasonably related to the supplements, the Investigator will promptly notify the Sponsor.

9 Participant Completion and Discontinuation

9.1 Participant Completion

The definition of a completed Participant is if they have completed all visits according to the protocol or have met the rules in the following sections 9.2, 9.3 and 9.4.

9.2 Stopping Rules / Discontinuation Criteria

Participants can be discontinued from study treatment and assessments at any time, at the discretion of the Investigator(s). Specific reasons for discontinuing a Participant from this study are:

- Withdrawal of informed consent
- Lost to follow-up
- Pregnancy in female Participant
- Protocol non-compliance

If the Investigator considers it is not in the best interest of the Participant to continue. Even if discontinued, Investigators will aim to follow up Participants at the pre-determined time points for trial assessments unless they specifically request in writing not to be conducted.

9.3 Voluntary Participant Withdrawal

Participants are free to discontinue their participation in the study at any time, irrespective of reason. The Participant is not obliged to state his or her reason for withdrawal. However, all efforts will be made to see Participants in follow-up at pre-determined time points for trial assessments after stopping the intervention. If Participants are not willing to attend follow-up appointments then permission to maintain phone contact will be sought, unless they request in writing not to do so.

9.4 Early Termination of the Study

The study may be terminated prematurely by the Principal Investigator (PI) or his/her designee and the Sponsor if:

- The number and/or severity of AEs justify discontinuation of the study
- New data become available which raise concern about the safety of the study drug, so that continuation might cause unacceptable risks to Participants.

In addition, the Sponsor and the administrator reserve the right to discontinue the trial prior to inclusion of the intended number of Participants, but intend only to exercise this right for valid scientific or administrative reasons.

After such a decision, the Investigator must contact all participating Participants within two weeks, and written notification must be sent to the HREC.

10 Case Report Form (CRF)

Source notes will be kept in the Participant files at the Boden Institute. Data from the source notes and clinic visits will be entered into the eCRF according to the CTU standard operating procedures. An eCRF will be completed for each study Participant for those assessments which are written on paper. In the case of data and time stamped source data captured electronically, this will be used as the source. In order to retain confidentiality, Participants will be referred to in the eCRF by their Participant number and initials.

The completed eCRF's will be retained by the Investigator for a period of time as determined by local regulations. A copy of the eCRF will be given to the Sponsor at the end of the study.

11 Data Analysis and Statistical Considerations

11.1 Hypotheses

That FBCx will be superior in cholesterol control to control group, and that Ginst15 will improve glycaemic control relative to control group.

11.2 Endpoints

The primary endpoints of this study are to determine:

- The efficacy of FBCx (a formula based on α-cyclodextrin (marketed under the trade name FBCx)) on cholesterol control, and
- The efficacy of Ginst15 (a ginseng extract formula based on Compound K) on glycaemic control.

11.3 Sample Size

A total of 400 Participants will be recruited into the study. The study is powered for the smallest difference between two of the groups for each outcome, with the larger sample size

being adopted. This also ensures that the sample size is sufficient to determine the effect of the combination treatment (FBCx and GinST15).

FBCx (a formula based on α-cyclodextrin): Assuming a mean reduction in fasting total cholesterol from baseline of 0.50 mmol/L in the intervention compared to control group, with a standard deviation (SD) of 0.90 mmol/L and an estimated drop-out rate of 15% (at 6 months), a total of 143 Participants would be required to achieve 80% power to detect a difference between the two groups (at a 2-sided significance level of 2.5%).

Ginst15 (a ginseng extract formula based on Compound K): Assuming a mean reduction in fasting plasma glucose from baseline of 0.47 mmol/L in the intervention compared to control group, with a standard deviation (SD) of 1 mmol/L and an estimated drop-out rate of 15% (at 6 months), a total of 200 Participants would be required to achieve 80% power to detect a difference between the two groups (at a 2-sided significance level of 2.5%).

Based on these calculations, 400 Participants (100 in each group) will be recruited and randomised to one of four treatment groups. All randomised Participants will be included in the final analysis.

11.4 Statistical Analysis

Data will be analysed for normality using the Shapiro-Wilk test. For analysis of differences between treatments, an analysis of covariance (ANCOVA), or the non-parametric equivalent will be used. For within group changes, repeated measures ANOVA will be used. As well as an analysis of completers (those that attend the final visit), an intention to treat (ITT) analysis will also be included. For the ITT analysis, dropouts will be treated using a variety of sensitivity analyses.

Interim analyses will be performed at end of intervention (month 6) and end of follow-up (month 12). Sub-group analyses will also be performed for those with hypercholesterolaemia, hyper-triglyceridaemia, hyper-lipidaemia and different BMI ranges.

With respect to weight loss, demonstration of a clinically significant degree of weight loss of at least 5-10% of baseline weight, which is also at least 5% greater than that associated with placebo, is considered to be a valid efficacy criterion in clinical trials evaluating new anti-obesity treatments.

12 Data Collection and Management

Data will be completed electronically using time and date stamped data collection tools. Questionnaires will be completed by participants on a platform called Qualtrics and source data will be entered directly by investigators at the time of each visit into OpenClinica. Paper food diaries will be collected and entered by participants on an electronic platform. Each of the platforms and the OpenClinica database will be password protected and backed up on The University of Sydney server.

Data collected from the "Australian Type 2 Diabetes Risk Screening Assessment Tool" (Appendix R) will be stored in a secured database (Oracle) which will be backed up on a daily basis on CPU and disc resources.

13 Monitoring and Quality Assurance

The task of the Sponsor is to guarantee the best conduct of the study through frequent contacts by phone and in person with the responsible Investigator with the purpose of facilitating the work and fulfilling the objectives of the study. These site visits will enable the Sponsor to maintain current, personal knowledge of the study through review of the records, comparison with source documents, and observation and discussion of the conduct of the study with the Investigator. The Sponsor is responsible for monitoring adherence to the Protocol and completion of the eCRF, and for the relationship between the Investigator and Sponsor.

In order to ensure the accuracy of data, direct access to source documents by representatives of the Sponsor and regulatory authorities is mandatory. Anonymity of the Participant will be maintained at all times. The Sponsor reserves the right to terminate the conduct of the study at any particular site for refusal of the Investigator/Institution to supply source documentation of work performed in the study.

13.1 Curriculum Vitae and Other Documentation

The present investigation may constitute a part of a national registration file. In order to comply with regulatory requirements in some countries, all Investigators signing the Protocol and all trial staff should provide a current, signed and dated Curriculum Vitae (CV) to be filed by the Sponsor. The CV should include name, title, occupation, education, research experience and present and former positions, be signed and dated every two years. A Staff

Signature List or delegation log is required. Principal Investigators may also be requested to complete a financial disclosure form.

14 Investigator Responsibility

Except where the Principal Investigator's signature is specifically required, it is understood that the term 'Investigator' as used in this Protocol and on the eCRF refers to the PI or an appropriately qualified member of the staff that the PI designates to perform specified duties of the Protocol as delegated on the Staff Signature List or delegation log. The PI is ultimately responsible for the conduct of all aspects of the study.

Each Investigator will comply with the local regulations and guidelines regarding the conduct of clinical trials and the Investigator responsibilities, including but not limited to, the International Conference on Harmonisation (ICH) GCP guidelines, as adopted by the Therapeutic Goods Administration (TGA).

15 Study Report

The final study report will be based on ICH Harmonised Tripartite guidelines E3.
http://www.ich.org/products/guidelines/efficacy/efficacy-single/article/structure-and-content-of-clinical-study-reports.html

16 Administrative Procedures

16.1 Ethical Considerations

Information on side effects of the test and reference formulations is summarised in the Investigator's Brochure or product insert. The monitoring and safety guidelines are outlined in the Monitoring Guidelines for the study. The amount of blood to be sampled in the study is not considered to be excessive in healthy adult Participants. This study will be carried out according to the principals of the Declaration of Helsinki, the National Health and Medical Research Council (NHMRC) National Statement on Ethical Conduct in Research Involving Humans and the Notes for Guidance on Good Clinical Practice as adopted by the Australian Therapeutic Goods Administration (2000) (CPMP/ICH/135/95) (GCP). These documents are available on the internet and will not be annexed to this Protocol.

16.2 Ethical Review Committee

The Protocol will be submitted for approval to the Sydney Local Health District HREC (Royal Prince Alfred Hospital), and written approval obtained, before volunteers are recruited and Participants are enrolled. The Investigators will receive all the documentation needed for submitting the present Protocol to the HREC. A copy of the respective approval letters will be transmitted to the Sponsor before starting the study. The composition of the HREC will also be provided to the Sponsor. If approval is suspended or terminated by the HREC, the Investigator will notify the Sponsor immediately.

It is the responsibility of the Investigator to report study progress to the HREC as required or at intervals not greater than one year.

The PI, or their nominee, will be responsible for reporting any serious adverse events to the HREC as soon as possible, and in accordance with the guidelines of the HREC.

The final study Protocol, including the final version of the Information for Participants, must be approved in writing by the Sydney Local Health District HREC (Royal Prince Alfred Hospital).

The PI is responsible for informing the HREC of any amendment to the Protocol. In addition, the HREC must approve all advertising used for recruitment for the study.

The PI must submit progress reports to the HREC as required.

16.3 Regulatory Authorities

This study will be notified to the Therapeutic Goods Administration (TGA) under the Clinical Trial Notification (CTN) scheme.

Any specific SAEs will be reported to the Sponsor as detailed in this Protocol. In agreeing to the provisions of the Protocol, these responsibilities are accepted by the Investigator.

16.4 Informed Consent

The PI, or a person designated by the PI on the delegation of responsibility sheet, should ensure that the Participant is given full and adequate oral and written information about the nature, purpose and possible risks and benefits of the study.

Participants must also be notified that they are free to discontinue the study at any time. The Participant should be given the opportunity to ask questions and should be allowed time to consider the information provided.

The Participant's signed and dated informed consent must be obtained before conducting any procedure specifically for the study. The consent forms shall be signed and dated by the appropriate parties.

A copy of the Participant Consent Form must be given to the Participant at the time of signing the document. A notation that written informed consent has been obtained will be made on the Participant's case notes. The completed consent forms will be retained by the Investigator for review by the Sponsor.

16.5 Participant Reimbursement

Participants will be given a $20.00 AUD food debit card at each study visit from Baseline through to Month 12. The food debit card works at many of the major supermarket chains and is encrypted to allow the purchase of food only.

If the study is terminated by Sponsor or the Investigator prior to completion or a Participant withdraws or is withdrawn from the study before completion, a pro-rata payment will be made at the discretion of the Investigator(s).

16.6 Emergency Contact with Investigators

All Participants will be provided with a Participant Emergency Contact Card (approved by the HREC) with contact details of whom to contact in the case of an emergency.

16.7 Notification of Primary Care Physician

There is no requirement for the PI to notify the primary care physician of the Participant's participation in the study.

16.8 Investigator Indemnification

The study is being conducted in accordance with the 'Guidelines for Compensation for Injury Resulting from Participation in a Company-Sponsored Clinical Trial' published by the Medicines Australia. The Sponsor will reimburse Participants for costs of medical care that occur as a result of complications directly related to participation in this study.

16.9 Payments

Any payments related to the conduct of the study are subject to a formal agreement between the Sponsor and the Investigator or Institution.

16.10 Protocol Amendments

No changes (amendments) to the Protocol may be implemented without prior approval from the Sponsor.

Changes and amendments to the Protocol can only be made by the Sponsor. Approval of amendments by the Institutional HREC is required prior to their implementation. In some instances, an amendment may require a change to a consent form. The Investigator must receive approval/advice of the revised consent form prior to implementation of the change. In addition, changes to the data collected, if required, will be incorporated in the amendment. The Investigator should not implement any changes to, or deviations from, the Protocol except where necessary to eliminate immediate hazard(s) to Participants.

It is the responsibility of the Investigator to submit the amendment to the HREC for their approval; written approval should be obtained and a copy provided to the Sponsor. The Sponsor is responsible for determining whether or not the local regulatory authority must be notified of the Protocol change. Completed and signed Protocol amendments will be circulated to all those who were on the circulation list for the original Protocol.

The original signed copy of amendments will be kept in the Investigator File with the original Protocol. It should be noted that where an amendment to the Protocol substantially alters the study design or the potential risks to the Participants, each Participant's consent to continue Participation should be obtained.

16.11 Protocol Compliance

The instructions and procedures specified in this Protocol require diligent attention to their execution. Should there be questions or consideration of deviation from the Protocol, clarification will be sought from the Sponsor. Any Participant treated in a manner that deviates from the Protocol, or who is admitted into the study but is not qualified according to the Protocol as amended by Sponsor and the Investigator, may be ineligible for analysis and thereby compromise the study.

Only when an emergency occurs that requires a departure from the Protocol for an individual will there be such a departure. The nature and reasons for the Protocol violation shall be recorded in the source notes and eCRF.

The Investigator and designees will comply with all applicable federal, state and local laws.

16.12 Archives: Retention of Study Records

All source documents, eCRFs and trial documentation will be kept by the Investigator for 15 years following the completion of the study.

Data Protection

The Information for Participants will explain that study data will be stored in a computer database, and that confidentiality will be maintained in accordance with national data legislation. Participants in this database will be identified by initials and Participant number only. The Information for Participants will also explain that, for data verification purposes, authorised representatives, a regulatory authority, or an ethics review committee may require direct access to parts of the hospital or practice records relevant to the study, including a Participant's medical history.

Record Retention

The PI will ensure that all study documentation remains confidential. The PI will also take measures to prevent accidental or premature destruction of these documents.

The PI will retain documents for 15 years following completion of the study.

Use and Completion of Participant Information

It is the responsibility of the PI to maintain accurate information and to record all observations and other data pertinent to the study. All notes should be completed in their entirety in a neat, legible manner to ensure accurate interpretation of the data.

Should a correction be made, the information must not be overwritten. The corrected information will be transcribed by the authorised person next to the previous value, initialled and dated.

16.13 Archives: Retention of Other Study Specific Samples

Blood samples will be stored at -80°C and used for analysis of gut hormones, adipokines, SCFA, and LPS at the end of the trial. All other bloods will be routinely measured during the trial by a commercial laboratory.

Stool samples will be stored at -20°C and used for analysis of faecal SCFA and LPS concentration as the study progresses. Faecal bacterial DNA extracted from the stool samples will be stored at -20°C and used for gut microbiota community profile.

All Participant files will be kept for 15 years from date of last visit.

17 References

Akao T., et al. Appearance of compound K, a major metabolite of ginsenoside Rb-1 by intestinal bacteria, in rat plasma after oral administration - Measurement of compound K by enzyme immunoassay. Biol. Pharm. Bull., 21, 245-249 (1998).

Andreoni, J., et al. 2002. Risk Preferences are not Time Preferences. American Economic Review, 102(7): 3357-76 (2002).

Artiss J., et al. The effects of a new soluble dietary fiber on weight gain and selected blood parameters in rats. Metabolism Clinical and Experimental, 55, 195-202 (2006).

Attele A. S., et al. Antidiabetic effects of Panax ginseng berry extract and the identification of an effective component. Diabetes, 51, 1851-1858 (2002).

Attele A. S., et al. Ginseng pharmacology: multiple constituents and multiple actions. Biochem. Pharmacol., 58, 1685-1693 (1999).

Averett S, "Labor market consequences: Employment, wages, disability, and absenteeism." In: Cawley, J. (ed.). The Oxford Handbook of the Social Science of Obesity. New York: Oxford (2011).

Bolt, D. M., Lu, Y., & Kim, J.-S. Measurement and Control of Response Styles Using Anchoring Vignettes: A Model-Based Approach. Psychological Methods. Advance online publication. http://dx.doi.org/10.1037/met0000016 (2014).

Buset, S. L., Walter, C., Friedmann, A., Weiger, R., Borgnakke, W. S. & Zitzmann, N. U. 2016. Are periodontal diseases really silent? A systematic review of their effect on quality of life. Journal of Clinical Periodontology, 43(4), 333-44.

Carver C., et al. Origins and functions of positive and negative affect: A control-process view. Psychological review, 97:19-35 (1990).

Cawley J, "The Impact of Obesity on Wages." Journal of Human Resources, 39(2): 451-474 (2004).

Cervone D. The role of self-referent cognitions in goal setting, motivation, and performance. (1993).

Cobb-Clark D., et al. Two economists' musings on the stability of locus of control. The Economic Journal 123(570); F358-F400 (2013).

Cobb-Clark D., et al. The stability of the Big-Five personality traits. Economics Letters 115(1); 11-15 (2012).

Cobb-Clark D., et al. Healthy habits: What explains the connection between diet, exercise, and locus of control? Journal of Economic Behavior & Organization 98; 1-28.19 (2014).

Cobb-Clark D., et al. Noncognitive Skills, Occupational Attainment, and Relative Wages. Labour Economics, 18(1),1-13 (2011).

Comerford K., et al. The beneficial effects on blood lipids and weight loss in healthy humans. Obesity, 19, 1200-1204 (2011).

Courtemanche C., et al. Impatience, Incentives and Obesity. The Economic Journal. 125(582); 1-31 (2014).

Chamberlain S.R., et al. Obesity and dissociable forms of impulsivity in young adults. CNS Spectrums. DOI: 10.1017/S109285291400062 (2015).

Dombrowski S.U., et al. Long term maintenance of weight loss with non-surgical interventions in obese adults: systematic review and meta-analyses of randomised controlled trials. BMJ. 2014;348.

Draft Guideline on clinical evaluation of medicinal products used in weight control EMA/CHMP/311805/2014 26 June 2014 1.

Dye, B. A. 2012. Global periodontal disease epidemiology. Periodontology 2000, 58(1), 10-25.

El Ghoch, M., Calugi, S. & Dalle Grave, R. 2016. The Effects of Low-Carbohydrate Diets on Psychosocial Outcomes in Obesity/Overweight: A Systematic Review of Randomized, Controlled Studies. Nutrients, 8(7).

Foster G., et al. Promoting more modest weight losses: a pilot study. Obesity research, 12:1271-1277 (2004).

Foster G., et al. What is a reasonable weight loss? Patients' expectations and evaluations of obesity treatment outcomes. Journal of Consulting and Clinical Psychology, 65:79 (1997).

Genco, R. J. & Genco, F. D. 2014. Common Risk Factors in the Management of Periodontal and Associated Systemic Diseases: The Dental Setting and Interprofessional Collaboration. Journal of Evidence Based Dental Practice, 14, Supplement(4-16.

Geng, J., et al. Ginseng for cognition. *Cochrane Database of Systematic Reviews*(12). doi:10.1002/14651858.CD007769.pub2 (2010).

Grant J.E., et al. Obesity and gambling: neurocognitive and clinical associations. Acta Psychiatr Scand, DOI: 10.1111/acps.12353 (2014).

Grunberger G., et al. The benefits of early intervention in obese diabetic patients with FBCx - a new dietary fibre. Diabetes/Metabolism Research and Reviews, 22, 56-62 (2007).

Guideline for Levels and Kinds of Evidence for Listed Medicines with Indications and Claims for Weight Loss Consultation draft 6 February 2009.

Higgins E., Self-discrepancy: a theory relating self and affect. Psychological Review. 94:319 (1987).

Holt C., et al. Risk Aversion and Incentive Effects. The American Economic Review, 92(5) (2002).

Ikeda S., et al. Hyperbolic discounting, the sign effect and the Body Mass Index. Journal of Health Economics, 29 (2): 268-284 (2010).

Jeffrey R., et al. Are smaller weight losses or more achievable weight loss goals better in the long term for obese patients? Journal of Consulting and Clinical Psychology, 66:641 (1998).

Kern M.L., et al. Conscientiousness, Career Success, and Longevity: A Lifespan Analysis. Annals of Behavioral Medicine 37, 154-163 (2009).

Lee, M., et al. Ginseng for cognitive function in Alzheimer's disease: a systematic review. *JAD, Journal of Alzheimer's Disease, 18*(2), 339-344. Retrieved from <Go to ISI>://CABI:20093302795 (2009).

Lee, S.-T., et al. Ponax Ginseng enhances cognitive performance in Alzheimer disease. *Alzheimer Disease & Associated Disorders, 22*(3), 222-226. doi:10.1097/WAD.0b013e31816c92e6 (2008).

Linde J., et al. Are unrealistic weight loss goals associated with outcomes for overweight women? Obesity Research, 12:569-576 (2004).

Locke E., et al. Building a Practically Useful Theory of Goal Setting and Task Motivation On November 9th, 2011· In Business Models, Business Theories. American Psychologist, 57:705-717 (2002).

Locke E., et al. A theory of goal setting & task performance. Vol 21: Prentice Hall Englewood Cliffs, NJ; (1990).

Loef M., et al. Midlife Obesity and Dementia: Meta-Analysis and Adjusted Forecast of

Dementia Prevalence in the United States and China. *Obesity* 21: E51-E5 (2013).

Maciel, S. S., Feres, M., Gonçalves, T. E. D., Zimmermann, G. S., Silva, H. D. P., Figueiredo, L. C. & Duarte, P. M. 2016. Does obesity influence the subgingival microbiota composition in periodontal health and disease? Journal of Clinical Periodontology, 43(12), 1003-1012.

Madigan C.D., et al. Study protocol: the effectiveness and cost effectiveness of a brief behavioural intervention to promote regular self-weighing to prevent weight regain after weight loss: randomised controlled trial (The LIMIT Study) BMC Public Health 2015;15 (530).

Madigan C.D., et al. Is self-weighing an effective tool for weight loss: a systematic literature review and meta-analysis. International Journal of Behaviour Nutrition and Physical Activity, 2015: 12 (104).

Meng X-F., et al. Midlife Vascular Risk Factors and the Risk of Alzheimer's Disease: A

Systematic Review and Meta-Analysis. *Journal of Alzheimers Disease* **42**: 1295-310 (2014).

Nascimento, G. G., Peres, K. G., Mittinty, M. N., Mejia, G. C., Silva, D. A., Gonzalez-Chica, D. & Peres, M. A. 2016. Obesity and Periodontal Outcomes: A Population-Based Cohort Study in Brazil. Journal of Periodontology, 1-17.

Note for Guidance on Good Clinical Practice (CPMP/GCP/135/95) annotated with Therapeutic Goods Administration (TGA) comments (DSEB, July 2000).

Pradeep, A. R., Nagpal, K., Karvekar, S. & Patnaik, K. 2015. Levels of lipocalin-2 in crevicular fluid and tear fluid in chronic periodontitis and obesity subjects. Journal of Investigative and Clinical Dentistry.

Roberts-Thomson, K. & Do, L. 2007. Oral health status, Australian Institute of Health and Welfare (Media and Publishing Unit).

Scott K. P., et al. Dietary fibre and the gut microbiota. Nutrition Bulletin, 33, 201-211 (2008).

Schurer S., Lifecycle patterns in the socioeconomic gradient of risk preferences. IZA Discussion Paper Nr 8821, IZA Bonn, Germany (2015).

Siervo M., et al. Intentional weight loss in overweight and obese individuals and cognitive function: a systematic review and meta-analysis. Obesity Reviews 12: 968-83 (2011).

Smith P., et al. Are time preference and body mass index associated? Evidence from the National Longitudinal Survey of Youth. Economics and Human Biology, 3(2): 259-270 (2005).

Standards of Medical Care in Diabetes-2012. American Diabetes Association. Diabetes Care, 35, supp 1. January 2012.

Steinberg D.M., et al. The efficacy of a daily self-weighing weight loss intervention using smart scales and email. Obesity. 2013;21(9):1789-1797

Stubbs J., et al. Problems in identifying predictors and correlates of weight loss and maintenance: implications for weight control therapies based on behaviour change. Obesity Reviews, 12:688-708 (2011).

Sumithran P., et al. Long-term persistence of hormonal adaptations to weight loss. N Eng J Med., 365, 1597-1604 (2011).

Suvan, J., D'aiuto, F., Moles, D. R., Petrie, A. & Donos, N. 2011. Association between overweight/obesity and periodontitis in adults. A systematic review. Obesity Reviews, 12(5), e381-e404.

Tawab M. A., et al. Degradation of ginsenosides in humans after oral administration. Drug Metab. Dispos., 31, 1065-1071 (2003).

Tuomilehto J, Lindström J, Eriksson JG, Valle TT, Hämäläinen H, Ilanne-Parikka P, Keinänen-Kiukaanniemi S, Laakso M, Louheranta A, Rastas M, Salminen V, Uusitupa M; Finnish Diabetes Prevention Study Group. Prevention of type 2 diabetes mellitus by changes in lifestyle among participants with impaired glucose tolerance. N Engl J Med., 344(18), 1343-50 (2001).

Tymula A., et al. Like cognitive function, decision making across the life span shows profound age-related changes. Proceedings of the National Academy of Sciences (PNAS) of the United States of America, 110(42), 17143-17148 (2013).

Van Ommen, B et al. Disposition of 14C-alpha-cyclodextrin in germ-free and conventional rats. Regul. Toxicol. Pharmacol. RTP 39 Suppl 1, 57–66 (2004).

Vuksan V., et al. Nutr. Metab. Cardiovasc. Dis., Herbal remedies in the management of diabetes: lessons learned from the study of ginseng. Nutr. Metab. Cardiovasc. Dis. 15, 149-160 (2005).

Wadden T., et al. Efficacy of Lifestyle Modification for Long-Term Weight Control. Obesity Research, 12:151-162 (2004).

Wadden T., et al. Great expectations:" I'm losing 25% of my weight no matter what you say". Journal of Consulting and Clinical Psychology,71:1084 (2003).

Wing R.R., et al. A self-regulation program for maintenance of weight loss. The New England Journal of Medicine. 2006;355:1563-71.

Yamada H., et al. Relating Risk Preference, Water Rewards, and Thirst: Wealth and Utility in Monkeys. PNAS, 110(39) (2013).

Yokozawa T., et al. Studies on the mechanism of the hypoglycemic activity of ginsenoside-Rb2 in streptozotocin-diabetic rats. Chem. Pharm. Bull., 33, 869-872 (1985).

Yoon S. H., et al. Anti-diabetic effects of compound K versus metformin versus compound K-metformin combination therapy in diabetic db/db mice. Biol. Pharm. Bull. 30, 2196-2200 (2007).

Zimmermann, G. S., Bastos, M. F., Dias Goncalves, T. E., Chambrone, L. & Duarte, P. M. 2013. Local and circulating levels of adipocytokines in obese and normal weight individuals with chronic periodontitis. Journal of Periodontology, 84(5), 624-633.

APPENDIX A Faecal Fat collection

Stool Specimen Collection

FREQUENTLY ASKED QUESTIONS

1. Why do we collect stool samples? What can it tell us?

A stool sample can tell us how well your body is working by allowing us to analyse your gut bacteria. Your gut bacteria can affect the digestion, absorption and metabolism of food, immune system function, hormone regulation and brain function. It may also affect how your body processes supplements and pharmaceuticals. Examining gut microbes may therefore help us to improve the effectiveness of these substances.

2. What do I need to do?

We need a 'snapshot' of your body's bacterial community. We would like you to collect stool samples and store them in your freezer at home before returning them at your scheduled clinic visits. You will be provided with a stool collection kit and protection gear.

3. How many samples do I need to collect?

You will be asked to return one sample at Baseline, Month 6 and Month 12 visits. Each sample will need to be collected within three days prior to your clinic visit.

3. Are there any risks to me or my family from handling my stool or storing it in my freezer?

Yes, but the risks are minimal and easily managed by common hygiene precautions (e.g. washing hands). Although the amount of bacteria in a stool sample is higher than normal levels of exposure, it does not expose you to anything new. Unless you have symptoms of gastrointestinal illness (e.g. diarrhoea), the stool essentially contains harmless bacteria.

IMPORTANT: If you currently have diarrhoea do not attempt to collect a sample or store it at home

4. Storing stool samples in my freezer sounds unhygienic. Does it smell? Will it spoil my food?

The risk of smell and contamination of your food is extremely minimal because the sample will be 'double-contained' and frozen. The stool will be contained in a jar which will be placed in a zip-locked bag. The stool jar will not leak if closed properly and the zip-locked bag will prevent direct contact with food. Frozen poo generally does not smell and the airtight zip-locked bag will also prevents gas contact.

5. Why do I need to freeze it?

Keeping the sample below freezing temperature (e.g. in the freezer at home) will preserve its condition so that bacteria and other biochemical composition do not change. If your freezer is unable to make ice, you should not collect or store the sample at home.

6. Collecting a stool sample sounds messy – is it hard?

Not really. Most people find that passing a bowel movement directly into the sample jar far is simpler than they expected. If you still find it difficult, you can pass the stool onto a clean surface or container (e.g. a paper-lined takeaway container), and transfer it to the jar. This method is also very easy and will leave no mess or smell.

Sampling Schedule

Please refer to Table 1 for when you have to pick-up your Stool Specimen Collection Kit (Provision Visits) and drop off your samples (Return Visits). On each of your Return Visits, you will return frozen stool samples collected at home 1-3 days before.

Table 1. Schedule for Stool Sample collection

	Screen	Baseline	Month 1	Month 2	Month 5	Month 6	Month 7	Month 8	Moth 9	Month 12
	Visit 1	Visit 2	Visit 3	Visit 4	Visit 7	Visit 8	Visit 9	Visit 10	Visit 11	Visit 12
**Provision Visit** Pick up stool collection kit	✓ (1 jar)				✓ (1 jar)				✓ (1 jar)	
**Return Visit** Drop-in frozen stool specimen to the clinic		✓ (1 stool)				✓ (1 stool)				✓ (1 stool)

The Stool Specimen Collection Kit

You will receive an insulated bag, in which you will find:

- 1 sterile stool jar with pre-printed label
- 1 zip lock plastic bag
- 1 pair latex gloves
- Specimen collection instructions

Date of Collection

This is the day you collect your sample and store it in your freezer at home. If you receive one stool collection jar, this means you collect one stool within three days before the return visit.

Example

You receive an insulated bag which contains one stool collection jar. Your next visit is Monday, 18 August 2015. You can collect your sample on either the Friday, Saturday,

or Sunday proceeding your next appointment. On Monday 18th August, return one frozen stool specimen to the clinic.

Please turn to the next page for your instructions >>>>

Instruction for Stool Collection

Please read the information carefully before collecting your stool sample

- Please put the stool specimen in the freezer immediately after collection.
- Use the pictures in the presentation to complement the steps explained in this instruction sheet.
- On your Return Visit, you must take the specimen from the freezer just before transport.

Before You Start

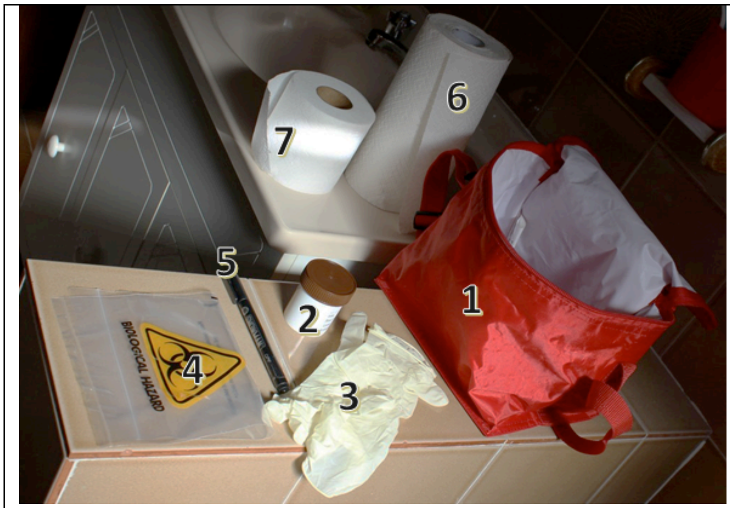

Make sure you have everything you need within arm's reach

You will receive:

1. Insulated bag
2. Stool collection jar
3. Gloves
4. Zip-lock bag

You have to provide:

5. Waterproof marker
6. Paper/kitchen towel
7. Toilet paper

11 Steps to Collect Your Stool Specimen

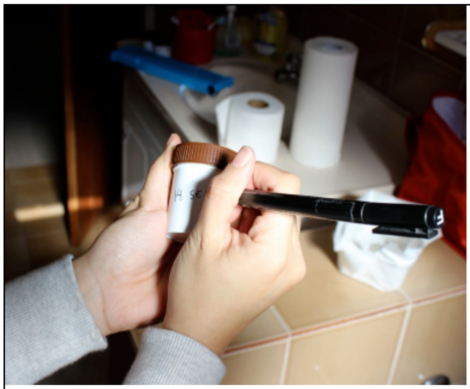	**STEP 1**<br>**Mark your jar**<br><br>Use the waterproof marker to write the date of collection on the jar (e.g. 14 SEPT 2014).
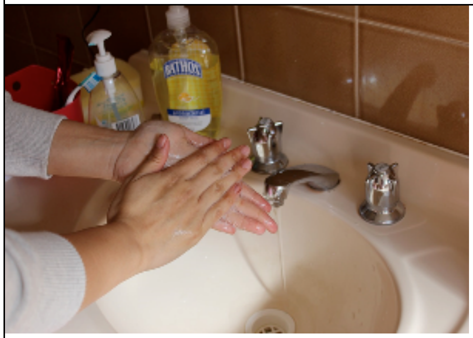	**STEP 2**<br>**Wash your hands**<br><br>Use soap and water. Lather and scrub for 20 seconds. Rinse with water for 10 seconds. Dry with clean paper towels
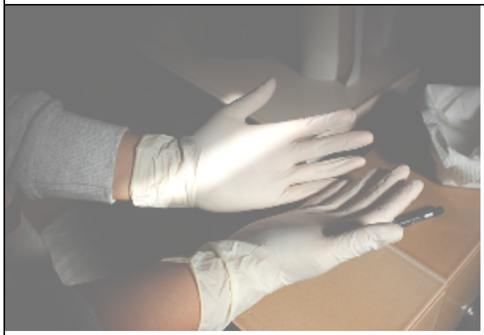	**STEP 3**<br>**Put on your gloves**<br><br>Latex can be delicate, so please do it carefully.

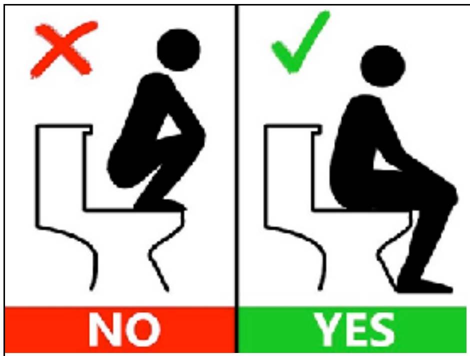

STEP 4

Sit on the toilet

To avoid contact of your sample with urine, it is best to urinate before you attempt to collect your stool. Flush the toilet after you urinate.

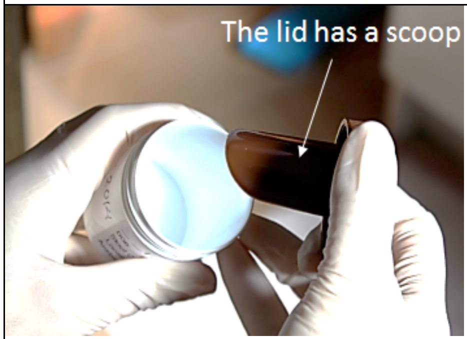

STEP 5

Open the stool sample Jar

Twist left to open.

The lid has a scoop attached to assist the collection process.

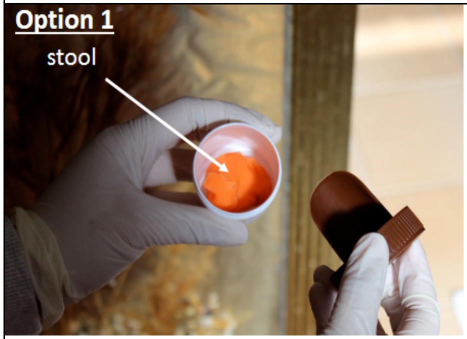

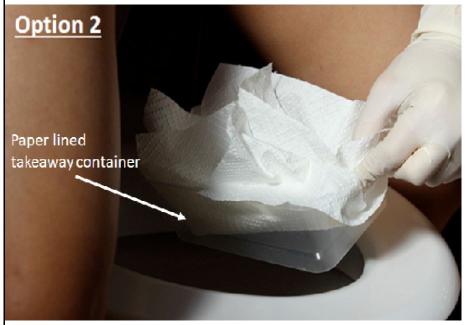

STEP 6

Collect your stool

Avoid contact with urine or water in the toilet.

Option 1

Hold the collection jar under your seat while sitting on the toilet. Pass stool directly into the jar. This method is preferred because it is the simplest and least messy.

Option 2

Hold a clean paper-lined takeaway container under your seat while sitting on the toilet. Pass your stool onto the container. Use the scoop on the jar lid to collect your sample.

<table>
<tr>
<td>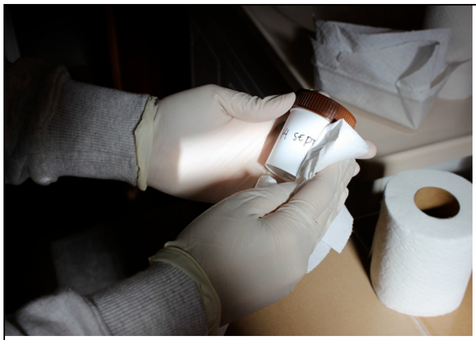</td>
<td><h2>STEP 7</h2><h2>Close and clean the jar</h2> Twist lid to the right to close firmly. Use toilet paper to clean the jar. </td>
</tr>
<tr>
<td>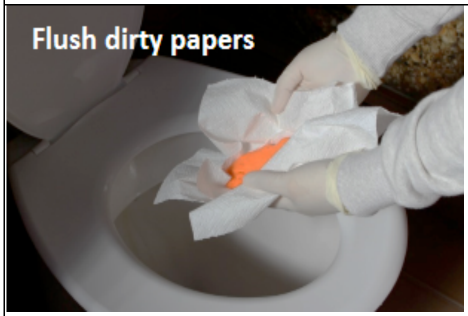

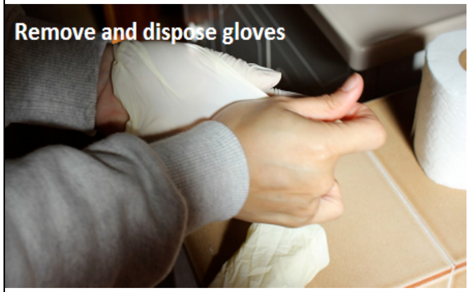
</td>
<td><h2>STEP 8</h2><h2>Clean Up</h2> Flush dirty papers and excess stool down the toilet. Remove your gloves and throw into the rubbish bin. </td>
</tr>
<tr>
<td>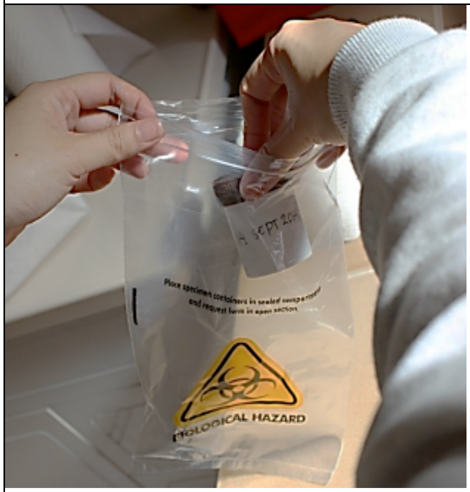</td>
<td><h2>STEP 9</h2><h2>Seal the stool jar in a zip-lock plastic bag</h2></td>
</tr>
</table>

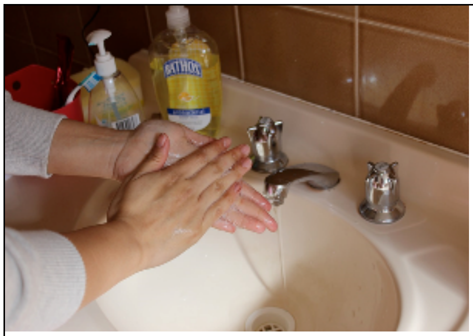

STEP 10

Wash your hands again

Use soap and water. Lather and scrub for 20 seconds. Rinse with water for 10 seconds. Dry with clean paper towels.

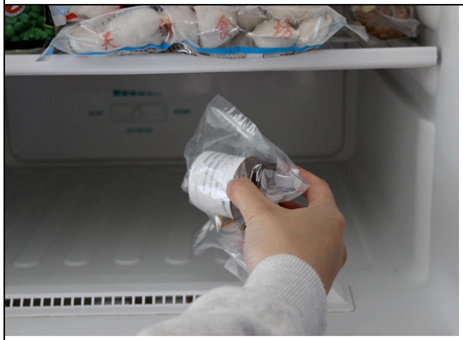

STEP 11

Store your specimen in the freezer <u>immediately</u>.

The stool has to be frozen overnight before returning it to the clinic.

<u>On the Day of Your Return Visit</u>

- ⚠ You should remove the specimen from the freezer <u>just before transport</u>.
- ⚠ Put the zip plastic bag (containing the stool jar) into the insulated bag and return it to the clinic.
- ⚠ Please take care not to leave your specimen at ambient temperature for longer than 2 hours.
- ⚠ Should your travel take longer than that, please notify the staff upon the return of the specimen at the clinic.

<u>You have completed the stool collection process.</u>

APPENDIX B SF-36® Health Survey

1. In general, would you say your health is:

Excellent	1
Very good	2
Good	3
Fair	4
Poor	5

(Circle One Number on Each Line)

2. Compared to one year ago, how would you rate your health in general now?

Much better now than one year ago	1
Somewhat better now than one year ago	2
About the same	3
Somewhat worse now than one year ago	4
Much worse now than one year ago	5

(Circle One Number on Each Line)

The following items are about activities you might do during a typical day. Does your health now limit you in these activities? If so, how much?

	Yes, Limited a Lot	Yes, Limited a Little	No, Not limited at All
3. Vigorous activities, such as running, lifting heavy objects, participating in strenuous sports	1	2	3
4. Moderate activities, such as moving a table, pushing a vacuum cleaner, bowling, or playing golf	1	2	3
5. Lifting or carrying groceries	1	2	3
6. Climbing several flights of stairs	1	2	3
7. Climbing one flight of stairs	1	2	3
8. Bending, kneeling, or stooping	1	2	3
9. Walking more than a mile	1	2	3
10. Walking several blocks	1	2	3
11. Walking one block	1	2	3
12. Bathing or dressing yourself	1	2	3

(Circle One Number on Each Line)

During the past 4 weeks, have you had any of the following problems with your work or other regular daily activities as a result of your physical health?

	Yes	No
13. Cut down the amount of time you spent on work or other activities	1	2
14. Accomplished less than you would like	1	2
15. Were limited in the kind of work or other activities	1	2
16. Had difficulty performing the work or other activities (for example, it took extra effort)	1	2

(Circle One Number on Each Line)

During the past 4 weeks, have you had any of the following problems with your work or other regular daily activities as a result of any emotional problems (such as feeling depressed or anxious)?

	Yes	No
17. Cut down the amount of time you spent on work or other activities	1	2
18. Accomplished less than you would like	1	2
19. Didn't do work or other activities as carefully as usual	1	2

(Circle One Number on Each Line)

20. During the past 4 weeks, to what extent has your physical health or emotional problems interfered with your normal social activities with family, friends, neighbors, or groups?

Not at all	1
Slightly	2
Moderately	3
Quite a bit	4
Extremely	5

(Circle One Number)

21. How much bodily pain have you had during the past 4 weeks?

None	1
Very mild	2
Mild	3
Moderate	4
Severe	5
Very severe	6

(Circle One Number)

22. During the past 4 weeks, how much did pain interfere with your normal work (including both work outside the home and housework)?

Not at all	1
Slightly	2
Moderately	3
Quite a bit	4
Extremely	5

(Circle One Number)

These questions are about how you feel and how things have been with you during the past 4 weeks. For each question, please give the one answer that comes closest to the way you have been feeling.

How much of the time during the past 4 weeks

	All of the Time	Most of the Time	A Good Bit of the Time	Some of the Time	A Little of the Time	None of the Time
23. Did you feel full of pep?	1	2	3	4	5	6
24. Have you been a very nervous person?	1	2	3	4	5	6
25. Have you felt so down in the dumps that nothing could cheer you up?	1	2	3	4	5	6
26. Have you felt calm and peaceful?	1	2	3	4	5	6
27. Did you have a lot of energy?	1	2	3	4	5	6
28. Have you felt downhearted and blue?	1	2	3	4	5	6
29. Did you feel worn out?	1	2	3	4	5	6
30. Have you been a happy person?	1	2	3	4	5	6
31. Did you feel tired?	1	2	3	4	5	6

(Circle One Number on Each Line)

32. During the past 4 weeks, how much of the time has your physical health or emotional problems interfered with your social activities (like visiting with friends, relatives, etc.)?

All of the time	1
Most of the time	2
Some of the time	3
A little of the time	4
None of the time	5

(Circle One Number)

How TRUE or FALSE is each of the following statements for you.

	Definitely True	Mostly True	Don't Know	Mostly False	Definitely False
33. I seem to get sick a little easier than other people	1	2	3	4	5
34. I am as healthy as anybody I know	1	2	3	4	5
35. I expect my health to get worse	1	2	3	4	5
36. My health is excellent	1	2	3	4	5

(Circle One Number on Each Line)

APPENDIX C Assessment of Quality of Life (AQOL) 8D Questionnaire

Tick the box that best describes your situation as it has been over the past week

aqol1 Thinking about how much energy you have to do the things you want to do: I am

- ❑ always full of energy
- ❑ usually full of energy
- ❑ occasionally energetic
- ❑ usually tired and lacking energy
- ❑ always tired and lacking energy

aqol2 How often do you feel socially excluded or left out?

- ❑ never
- ❑ rarely
- ❑ sometimes
- ❑ often
- ❑ always

aqol3 Thinking about how easy or difficult it is for you to get around by yourself outside your house (e.g., shopping, visiting):

- ❑ getting around is enjoyable and easy
- ❑ I have no difficulty getting around outside my house
- ❑ a little difficulty
- ❑ moderate difficulty
- ❑ a lot of difficulty
- ❑ I cannot get around unless somebody is there to help me

aqol4 Thinking about your health and your role in your community (that is to say neighbourhood, sporting, work, church or cultural groups):

- ❑ my role in the community is unaffected by my health
- ❑ there are some parts of my community role I cannot carry out
- ❑ there are many parts of my community role I cannot carry out
- ❑ I cannot carry out any part of my community role

aqol5 How often do you feel sad?

- ❑ never
- ❑ rarely
- ❑ some of the time
- ❑ usually
- ❑ nearly all the time

aqol6 Thinking about how often you experience serious pain: I experience it

- ❑ very rarely
- ❑ less than once a week
- ❑ three to four times a week
- ❑ most of the time

aqol7 How much confidence do you have in yourself?

- ❑ Complete confidence
- ❑ A lot
- ❑ A moderate amount
- ❑ A little
- ❑ None at all

aqol8 When you think about whether you are calm and tranquil or agitated: I am

- ❑ always calm and tranquil
- ❑ usually calm and tranquil
- ❑ sometimes calm and tranquil, sometimes agitated
- ❑ usually agitated
- ❑ always agitated

aqol9 Thinking about your health and your relationship with your family:

- ❑ my role in the family is unaffected by my health
- ❑ there are some parts of my family role I cannot carry out
- ❑ there are many parts of my family role I cannot carry out
- ❑ I cannot carry out any part of my family role

aqol10 Your close relationships (family and friends) are:

- ❑ very satisfying
- ❑ satisfying
- ❑ neither satisfying nor dissatisfying
- ❑ dissatisfying
- ❑ unpleasant
- ❑ very unpleasant

aqol11 When you communicate with others, e.g. by talking, listening, writing or signing:

- ❑ I have no trouble speaking to them or understanding what they are saying
- ❑ I have some difficulty being understood by people who do not know me. I have no trouble understanding what others are saying to me
- ❑ I am understood only by people who know me well. I have great trouble understanding what others are saying to me.
- ❑ I cannot adequately communicate with others

Tick the box that best describes your situation as it has been over the past week

aqol12 How often do you have trouble sleeping?

- ❑ never
- ❑ almost never
- ❑ sometimes
- ❑ often
- ❑ all the time

aqol13 How often do you feel worthless?

- ❑ never
- ❑ almost never
- ❑ sometimes
- ❑ usually
- ❑ always

aqol14 How often do you feel angry?

- ❑ never
- ❑ almost never
- ❑ sometimes
- ❑ often
- ❑ all the time

aqol15 Thinking about your mobility, including using any aids or equipment such as wheelchairs, frames, sticks:

- ❑ I am very mobile
- ❑ I have no difficulty with mobility
- ❑ I have some difficulty with mobility (for example, going uphill)
- ❑ I have difficulty with mobility. I can go short distances only
- ❑ I have a lot of difficulty with mobility. I need someone to help me
- ❑ I am bedridden

aqol16 Do you ever feel like hurting yourself?

- ❑ never
- ❑ rarely
- ❑ sometimes
- ❑ often
- ❑ all the time

aqol17 How enthusiastic do you feel?

- ❑ extremely
- ❑ very
- ❑ somewhat
- ❑ not much
- ❑ not at all

aqol18 And still thinking about the last seven days, how often did you feel worried?

- ❑ never
- ❑ occasionally
- ❑ sometimes
- ❑ often
- ❑ all the time

aqol19 Thinking about washing yourself, toileting, dressing, eating or looking after your appearance:

- ❑ these tasks are very easy for me
- ❑ I have no real difficulty in carrying out these tasks
- ❑ I find some of these tasks difficult, but I manage to do them on my own
- ❑ many of these tasks are difficult, and I need help to do them
- ❑ I cannot do these tasks by myself at all

aqol20 How often do you feel happy?

- ❑ all the time
- ❑ mostly
- ❑ sometimes
- ❑ almost never
- ❑ never

aqol21 How much do you feel you can cope with life's problems?

- ❑ completely
- ❑ mostly
- ❑ partly
- ❑ very little
- ❑ not at all

aqol22 How much pain or discomfort do you experience:

- ❑ none at all
- ❑ I have moderate pain
- ❑ I suffer from severe pain
- ❑ I suffer unbearable pain

aqol23 How much do you enjoy your close relationships (family and friends)?

- ❑ immensely
- ❑ a lot
- ❑ a little
- ❑ not much
- ❑ I hate it

Tick the box that best describes your situation as it has been over the past week

aqol24 How often does pain interfere with your usual activities?

- ❑ never
- ❑ rarely
- ❑ sometimes
- ❑ often
- ❑ always

aqol25 How often do you feel pleasure?

- ❑ always
- ❑ usually
- ❑ sometimes
- ❑ almost never
- ❑ never

aqol26 How much of a burden do you feel you are to other people?

- ❑ Not at all
- ❑ A little
- ❑ A moderate amount
- ❑ A lot
- ❑ totally

aqol27 How content are you with your life?

- ❑ extremely
- ❑ mainly
- ❑ moderately
- ❑ slightly
- ❑ not at all

aqol28 Thinking about your vision (using your glasses or contact lenses if needed):

- ❑ I have excellent sight
- ❑ I see normally
- ❑ I have some difficulty focusing on things, or I do not see them sharply e.g. small print, a newspaper or seeing objects in the distance
- ❑ I have a lot of difficulty seeing things. My vision is blurred. I can see just enough to get by with
- ❑ I only see general shapes. I need a guide to move around
- ❑ I am completely blind

aqol29 How often do you feel in control of your life?

- ❑ always
- ❑ mostly
- ❑ sometimes
- ❑ only occasionally
- ❑ never

aqol30 How much help do you need with jobs around the house (e.g. preparing food, cleaning the house or gardening):

- ❑ I can do all these tasks very quickly and efficiently without any help
- ❑ I can do these tasks relatively easily without help
- ❑ I can do these tasks only very slowly without help
- ❑ I cannot do most of these tasks unless I have help
- ❑ I can do none of these tasks by myself

aqol31 How often do you feel socially isolated?

- ❑ never
- ❑ rarely
- ❑ sometimes
- ❑ often
- ❑ always

aqol32 Thinking about your hearing (using your hearing aid if needed):

- ❑ I have excellent hearing
- ❑ I hear normally
- ❑ I have some difficulty hearing or I do not hear clearly. I have trouble hearing softly-spoken people or when there is background noise
- ❑ I have difficulty hearing things clearly. Often I do not understand what is said. I usually do not take part in conversations because I cannot hear what is said
- ❑ I hear very little indeed. I cannot fully understand loud voices speaking directly to me
- ❑ I am completely deaf

aqol33 How often do you feel depressed?

- ❑ never
- ❑ almost never
- ❑ sometimes
- ❑ often
- ❑ very often
- ❑ all the time

aqol34 Your close and intimate relationships (including any sexual relationships) make you:

- ❑ very happy
- ❑ generally happy
- ❑ neither happy nor unhappy
- ❑ generally unhappy
- ❑ very unhappy

aqol35 How often did you feel in despair over the last seven days?

- ❑ never
- ❑ occasionally
- ❑ sometimes
- ❑ often
- ❑ all the time

APPENDIX D Patients' Global Impression of Change (PGIC) Scale

Date: ______________________

Name: ____ DOB: ________________

Chief Complaint (Presenting Problem): ____

Since beginning treatment at this clinic, how would you describe the change (if any) in ACTIVITY LIMITATIONS, SYMPTOMS, EMOTIONS, and OVERALL QUALITY OF LIFE, related to your painful condition? Please circle the number below, that matches your degree of change since beginning care at this clinic for the above stated chief complaint.

No change	Almost the same	A little better	Somewhat better	Moderately better	Better	A great deal better
1	2	3	4	5	6	7

Explanation:

1 = No change (or condition has got worse)

2 = Almost the same, hardly any change at all

3 = A little better, but no noticeable change

4 = Somewhat better, but the change has not made any real difference

5 = Moderately better, and a slight but noticeable change

6 = Better, and a definite improvement that has made a real and worthwhile difference

7 = A great deal better, and a considerable improvement that has made all the difference

Patient's signature: ____

NOTE TO HEALTH CARE PROVIDER

A significant, favorable change is a score of 5-7

No significant change is a 1-4 response.

Note, this a dichotomous scale (5-7 = yes; 1-4 = no).

A 2-point change is significant from their last reported score.

Reference: Hurst H, Bolton J. Assessing the clinical significance of change scores recorded on subjective outcome measures. Journal of Manipulative Physiological Therapeutics (JMPT) 2004;27:26-35.

APPENDIX E Pain rating scales

Visual analogue scale (VAS)

Instructions for producing a VAS Bedside card:

OPTION 1

- Double-side print or photocopy the next two diagrams ensuring that the lines are exactly 10cm in length and superimposed
- Laminate the VAS Bedside card for patient use

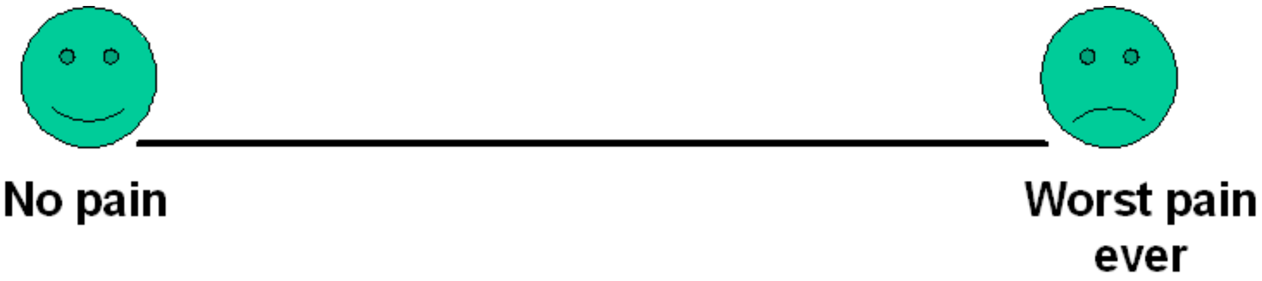

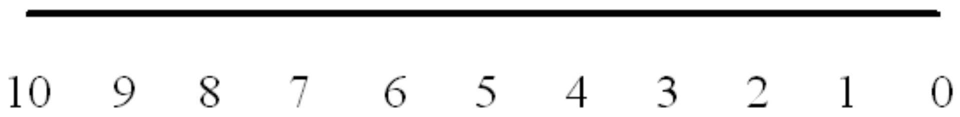

PLEASE NOTE: For purposes of double-sided print, the numbers on this scale are reversed.

OPTION 2

- Print or photocopy the next two diagrams on an A4 sheet ensuring that the lines are exactly 10cm in length
- Fold at the dotted line
- Do not show the patient the numbered scale

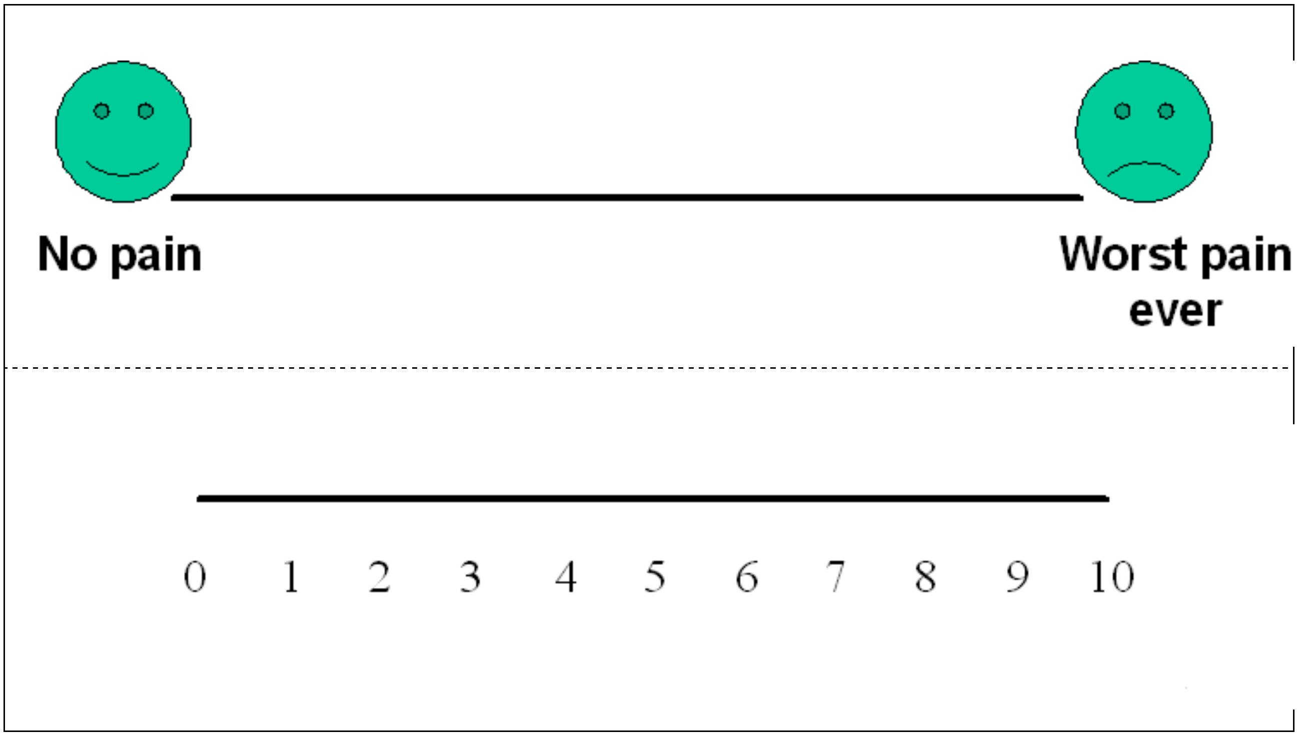

Numerical rating scale (NRS)

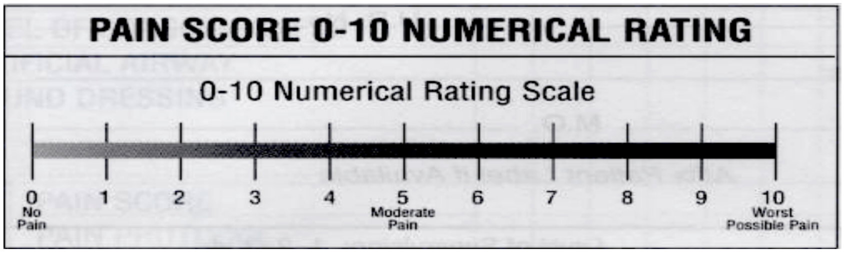

Faces rating scale (FRS)

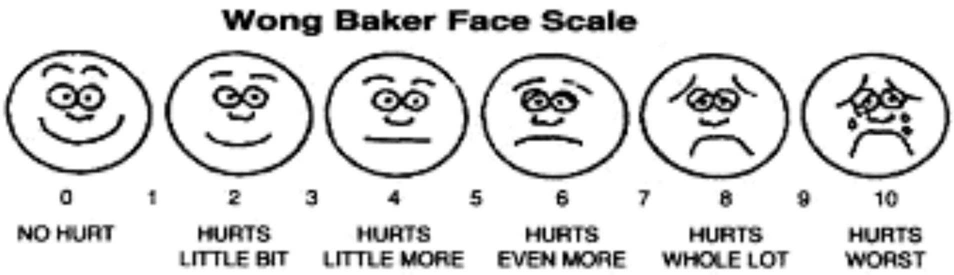

Behavioural rating scale

For patients unable to provide a self-report of pain: scored 0–10 clinical observation

**Face**	**0**<br>Face muscles relaxed	**1**<br>Facial muscle tension, frown, grimace	**2**<br>Frequent to constant frown, clenched jaw	**Face score:**
**Restlessness**	**0**<br>Quiet, relaxed appearance, normal movement	**1**<br>Occasional restless movement, shifting position	**2**<br>Frequent restless movement may include extremities or head	**Restlessness score:**
**Muscle tone***	**0**<br>Normal muscle tone	**1**<br>Increased tone, flexion of fingers and toes	**2**<br>Rigid tone	**Muscle tone score:**
**Vocalisation****	**0**<br>No abnormal sounds	**1**<br>Occasional moans, cries, whimpers and grunts	**2**<br>Frequent or continuous moans, cries, whimpers or grunts	**Vocalisation score:**
**Consolability**	**0**<br>Content, relaxed	**1**<br>Reassured by touch, distractible	**2**<br>Difficult to comfort by touch or talk	**Consolability score:**
**Behavioural pain assessment scale total (0–10)**				**/10**

Functional activ
score#

(Cough/movement)

A – No limitation
B – Mild limitation

C – Severe limitatic

#Relative to baselir

* Assess muscle tone in patients with spinal cord lesion or injury at a level above the lesion injury. Assess patients with hemiplegia on the unaffected side.

** This item cannot be measured in patients with artificial airways.

Pain rating scales instructions:

Subjective pain score

All patients are to have a functional activity score recorded in addition to the chosen subjective score.

Visual analogue scale (VAS)

Instruct the patient to point to the position on the line between the faces to indicate how much pain they are currently feeling. The far left end indicates ‘No pain’ and the far right end indicates ‘Worst pain ever’.

Numerical rating scale (NRS)

Instruct the patient to choose a number from 0 to 10 that best describes their current pain. 0 would mean ‘No pain’ and 10 would mean ‘Worst possible pain’.

Faces rating scale (FRS)

Adults who have difficulty using the numbers on the visual/numerical rating scales can be assisted with the use of the six facial expressions suggesting various pain intensities. Ask the patient to choose the face that best describes how they feel. The far left face indicates ‘No hurt’ and the far right face indicates ‘Hurts worst’. Document number below the face chosen.

Behavioural rating scale

The behavioural pain assessment scale is designed for use with non-verbal patients unable to provide self-reports of pain.

- Rate each of the five measurement categories (0, 1 or 2)
- Add these together
- Document the total pain score out of 10

Functional activity score

This is an activity-related score. Ask your patient to perform an activity related to their painful area (for example, deep breathe and cough for thoracic injury or move affected leg for lower limb pain).

Observe your patient during the chosen activity and score A, B or C.

A – No limitation meaning the patient’s activity is unrestricted by pain

B – Mild limitation means the patient’s activity is mild to moderately restricted by pain

C – Severe limitation means the patient ability to perform the activity is severely limited by pain

*Relative to baseline refers to any restriction above any pre–existing condition the patient may already have.

APPENDIX F Weight Locus of Control (WLOC) Questionnaire

		Strongly disagree	Disagree	Tend to disagree	Tend to agree	Agree	Strongly agree
1	Whether I gain, lose or maintain my weight is entirely up to me	**1**	**2**	**3**	**4**	**5**	**6**
2	Being the right weight is largely a matter of good fortune	**1**	**2**	**3**	**4**	**5**	**6**
3	No matter what I intend to do, if I gain or lose weight, or stay the same in the near future, it is just going to happen	**1**	**2**	**3**	**4**	**5**	**6**
4	If I eat properly, and get enough exercise and rest, I can control my weight in the way I desire	**1**	**2**	**3**	**4**	**5**	**6**

APPENDIX G Target Weight (Goal Weight)

Do you have a target weight (or goal weight) for the present weight loss attempt?

- ❑ Yes
- ❑ No

If No Is Selected, Then Skip To How much do you expect to weigh in 6 months?

What is your target weight (in kilograms) for the present weight loss attempt in 6 months time? (Please do not enter your goal weight loss, but rather your goal weight).

APPENDIX H Goal Commitment Scale

Please select the number that best describes how confident you are that the statement is true of you.

	1 (Strongly disagree) (1)	2 (2)	3 (3)	4 (4)	5 (Strongly agree) (5)
It's hard to take this goal seriously (1)	❍	❍	❍	❍	❍
Quite frankly, I don't care if I achieve this goal. (2)	❍	❍	❍	❍	❍
I am strongly committed to this goal. (3)	❍	❍	❍	❍	❍
It wouldn't take much to make me abandon this goal. (4)	❍	❍	❍	❍	❍
I think this is a good goal to shoot for. (5)	❍	❍	❍	❍	❍

APPENDIX I Effort Scale

Please select the number that best describes how confident you are that the statement is true of you.

	1 (Not at all)	2	3	4	5	6	7	8 (Very much)
How much effort will you invest in the next 6 months in achieving your target weight?	❍	❍	❍	❍	❍	❍	❍	❍
To what extent will you do your best to attain your target weight in the next 6 months?	❍	❍	❍	❍	❍	❍	❍	❍
How much energy will you spend in achieving your target weight in the next 6 months?	❍	❍	❍	❍	❍	❍	❍	❍

APPENDIX J Expected Weight

How much do you expect to weigh (in kg) in 6 months time?

APPENDIX K General Self-Efficacy Scale

Please select the number that best describes how confident you are that the statement is true of you.

	1 (Not at all true) (1)	2 (2)	3 (3)	4 (Exactly true) (4)
I can always manage to solve difficult problems if I try hard enough. (1)	❍	❍	❍	❍
If someone opposes me, I can find the means and ways to get what I want. (2)	❍	❍	❍	❍
It is easy for me to stick to my aims and accomplish my goals. (3)	❍	❍	❍	❍
I am confident that I could deal efficiently with unexpected events. (4)	❍	❍	❍	❍
Thanks to my resourcefulness, I know how to handle unforeseen situations. (5)	❍	❍	❍	❍
I can solve most problems if I invest the necessary effort. (6)	❍	❍	❍	❍
I can remain calm when facing difficulties because I can rely on my coping	❍	❍	❍	❍

abilities. (7)				
When I am confronted with a problem, I can usually find several solutions. (8)	❍	❍	❍	❍
If I am in trouble, I can usually think of a solution. (9)	❍	❍	❍	❍
I can remain calm when facing difficulties because I can rely on my coping abilities. (10)	❍	❍	❍	❍
I can usually handle whatever comes my way. (11)	❍	❍	❍	❍

APPENDIX L Five Factor Model and Vignettes

A1 How well do the following words describe you? For each word, cross one box to indicate how well that word describes you. There are no right or wrong answers.

Does not describe me at all						Describes me very well
1	2	3	4	5	6	7
Tick X one box for each word						

A1a Talkative

A1b Sympathetic

A1c Orderly

A1d Envious

A1e Deep

A1f Withdrawn

A1g Harsh

A1h Systematic

A1i Moody

A1j Philosophical

A1k Bashful

A1l Kind

A1m Inefficient

A1n Touchy

A1o Creative

A1p Quiet

A1q Cooperative

A1r Sloppy

A1s Jealous

A1t Intellectual

A1u Extroverted

A1v Cold

A1w Disorganized

A1x Temperamental

A1y Complex

A1z Shy

A1aa Warm

A1ab Efficient

A1ac Fretful

A1ad Imaginative

A1ae Enthusiastic

A1af Selfish

A1ag Careless

A1ah Calm

A1ai Traditional

A1aj Lively

B Your perceptions about others

Below you will find descriptions of the behaviour of three people. Please rate for each person his or her personality similar to how you have rated yourself in Part A.

[Note: Survey participant will be presented with three randomly chosen sketches from the seven sketches listed below]

B1 How well do the following words describe [Name]. For each word, cross once box to indicate how well that word describes [Name]. There are no right or wrong answers.

Does not describe me at all						Describes me very well
1	2	3	4	5	6	7
Tick X one box for each word						

**Adjectives**	**Name 1**	**Name 2**	**Name 3**
C1a Orderly			
C1b Philosophical			
C1c Systematic			
C1d Inefficient			
C1e Creative			
C1f Sloppy			
C1g Intellectual			
C1h Disorganized			
C1i Complex			
C1j Imaginative			
C1k Efficient			
C1l Careless			
C1m Deep			

[Hypothetical person sketch starts here: 3/8 persons are randomly selected and out of these we randomly assign a female or male gender – see below for gender-reversed vignettes]

a) [Mary] runs a company she founded on her own, raises three children and takes care of her household meticulously. In addition, she is active in sports and in community life. Despite her wide range of activities, she has time for her parents and to go hiking with friends. She likes reading and discussing philosophy and experimenting with new foods.
b) Already as a child [Anette] wanted to become a doctor. At school she was a moderate student lacking depth and creativity and her teachers did not believe she would be admitted to university. She did not succeed the first time, but [Anette] did not give up, she worked as an orderly at a hospital for a year, took private lessons and at second attempt she was admitted to university. Presently [Anette] is a registered doctor and the manager of a small practice.
c) [Nancy] discontinued her studies and she hasn't been able to find a steady job for 10 years. She lives with her parents, who have difficulty coping financially. Due to being overweight [Nancy] has tried many diets unsuccessfully, she now has heart problems and doctors have advised her to be physically active. In spite of this [Nancy] seldom leaves the house and most of the day she watches TV.
d) Generally [Allan's] friends trust him and enjoy his company because of his ability to think deep and see things from different perspectives. Sometimes, however, they have been really annoyed by him. For example, [Allan] does not always return the things he has borrowed on time. Sometimes he completely forgets about his promises.
e) Five years ago [Tom] finished his medical studies at the university and started working as a surgeon in a local hospital. His colleagues consider him a very good surgeon and lately he was appointed department head in the hospital. In case of problems [Tom] is very dependable. According to [Tom's] wife and her friends, who work as artists and graphic designers, he lacks creativity and rarely tries out new experiences
f) Since childhood [Bruno] has wanted to achieve a lot in his life and he has worked a lot for it. Despite extreme poverty at his parental home [Bruno] managed to get a good education. Continuous self-education and long hours at work have made him a very valued specialist and he has received ever better job offers. [Bruno] enjoys reading different newspapers to broaden his views.
g) [Jeanette] is a very creative young girl. She loves reading and writing, and taking her own time to develop her thoughts. She has been a member of a writer's club for many years, and has written several short stories. [Jeanette] is good in school, but she often daydreams during class, arrives late, and has difficulty meeting deadlines.
h) [Gerry] used to be a handsome man and competitive tennis player in his early 20s. Now in his late 30s he watches a lot of TV and enjoys a drink with his friends, although he doesn't like meeting new people. He works as a key account manager of a large wealth management firm. [Gerry] is reliable in his day-to-day job duties, but does not take the initiative to improve his performance or learn new things.

Reverse gender

a) [Mark] runs a company he founded on his own, raises three children and takes care of his household meticulously. In addition, he is active in sports and in community life. Despite his wide range of activities, he has time for his parents and to go hiking with friends. He likes reading and discussing philosophy and experimenting with new foods.
b) Already as a child [Adam] wanted to become a doctor. At school he was a moderate student lacking depth and creativity and his teachers did not believe he would be admitted to university. He did not succeed the first time, but [Adam] did not give up, he worked as an orderly at a hospital for a year, took private lessons and at second attempt he was admitted to university. Presently [Adam] is a registered doctor and the manager of a small practice.
c) [Nick] discontinued his studies and he hasn't been able to find a steady job for 10 years. He lives with his parents, who have difficulty coping financially. Due to being overweight [Nick] has tried many diets unsuccessfully, he now has heart problems and doctors have advised him to be physically active. In spite of this [Nick] seldom leaves the house and most of the day he watches TV.
d) Generally [Amy's] friends trust her and enjoy her company because of her ability to think deep and see things from different perspectives. Sometimes, however, they have been really annoyed by her. For example, [Amy] does not always return the things she has borrowed on time. Sometimes she completely forgets about her promises.
e) Five years ago [Tina] finished her medical studies at the university and started working as a surgeon in a local hospital. Her colleagues consider her a very good surgeon and lately she was appointed department head in the hospital. In case of problems [Tina] is very dependable. According to [Tina's] husband and his friends, who work as artists and graphic designers, she lacks creativity and rarely tries out new experiences
f) Since childhood [Beth] has wanted to achieve a lot in her life and she has worked a lot for it. Despite extreme poverty at her parental home [Beth] managed to get a good education. Continuous self-education and long hours at work have made her a very valued specialist and she has received ever better job offers. [Beth] enjoys reading different newspapers to broaden her views.
g) [Jim] is a very creative young boy. He loves reading and writing, and taking his own time to develop his thoughts. He has been a member of a writer's club for many years, and has written several short stories. [Jim] is good in school, but he often daydreams during class, arrives late, and has difficulty meeting deadlines.
h) [Gwyneth] used to be a beautiful woman and competitive tennis player in her early 20s. Now in her late 30s she watches a lot of TV and enjoys a drink with her friends, although she doesn't like meeting new people. She works as a key account manager of a large wealth management firm. [Gwyneth] is reliable in her day-to-day job duties, but does not take the initiative to improve her performance or learn new things.

APPENDIX M Risk and Time Preferences

Evaluation of Risk and Time Preferences

General Instructions

You will now have a one off opportunity to make some extra money. There are two short tasks. The first task has 10 questions and the second task has 20 questions. All questions involve you making a choice between two options. All together you will make 30 choices. You will choose between different monetary options for each question. After you finish answering all the questions, the computer will randomly generate a number between 1 and 30. The number that the computer generates determines which of your 30 choices will count towards your payment and whether you will be paid at all. You will find out the money you have won at the end of the task. Each decision is equally likely to be chosen. The money will be paid to you in cash during your visit at the CPC or at an indicated date during your follow-up visits at the CPC.

There are no right or wrong answers and people differ in what they choose. By choosing honestly the option that you prefer in every case, you can make sure that you get that payment that reflects your choice. You will be making your choices on your own and we will not show them to anybody else in your family.

Task 1 Instructions

For this task, choose between either the option on the left or the option on the right. You should have **one** answer for *every* question. Choosing the option on the left means you get paid that amount for sure, choosing the option on the right means you get to play a lottery later.

Payment for this task will be calculated in the following ways:

- If you chose the option on the left '$15 for sure', this means you will get that amount ($15).
- If you chose the lottery (option on the right), you have a 50/50 chance of getting the lottery amount or $0 (nothing). To decide whether you win or lose, the computer generates randomly a number 0 or 1. If the number is 1you will receive the lottery amount. If the number is 0, you will receive $0 (nothing).

Example 1: If you were presented with this choice:

☐	$5 for sure	*or*	☐	50% chance of $15

If this question was the one the computer generates for you to win and if you chose the option on the left, you would get $5 now. If you chose the option on the right, you would have an equal chance of getting $5 or getting nothing.

Task 1

Question number					
1	☐	$15 for sure	*or*	☐	50% chance of $15
2	☐	$15 for sure	*or*	☐	50% chance of $24
3	☐	$15 for sure	*or*	☐	50% chance of $27
4	☐	$15 for sure	*or*	☐	50% chance of $34
5	☐	$15 for sure	*or*	☐	50% chance of $38
6	☐	$15 for sure	*or*	☐	50% chance of $46
7	☐	$15 for sure	*or*	☐	50% chance of $57
8	☐	$15 for sure	*or*	☐	50% chance of $63
9	☐	$15 for sure	*or*	☐	50% chance of $77
10	☐	$15 for sure	*or*	☐	50% chance of $86

Task 2 Instructions

Choose between the option on the left and the option on the right. You should have one answer for every single question. In this task, you will answer questions about receiving money in the future. If you end up being paid based on your choice to a question 11-30, the money will be paid to you at the specified follow-up visit. For all payment dates (today, in 1 month, in 3 months, and in 4 months) we are using the same payment method – Next business day transfer into your bank account at the indicated date.

Example 1: If you were presented with this choice:

☐	$5 today	*or*	☐	$6 in 3 months (90 days)

If this question was the one the computer generates for you to win and if you chose the option on the left, we would transfer $5 to you later today. If you chose the option on the right, we would transfer $6 to you in 3 months.

Example 2: If you were presented with this choice:

☐	$5 in 1 month	*or*	☐	$10 in 4 months (120 days)

If this question was the one the computer generates for you to win and you chose the option on the left, you would get $5 transferred to you in one month. If you chose the option on the right, we would transfer $10 to you in 4 months.

Question number					
11	☐	$40 today	*or*	☐	$43 in 3 months (90 days)
12	☐	$40 today	*or*	☐	$46 in 3 months (90 days)
13	☐	$40 today	*or*	☐	$48 in 3 months (90 days)
14	☐	$40 today	*or*	☐	$51 in 3 months (90 days)
15	☐	$40 today	*or*	☐	$56 in 3 months (90 days)
16	☐	$40 today	*or*	☐	$61 in 3 months (90 days)
17	☐	$40 today	*or*	☐	$66 in 3 months (90 days)
18	☐	$40 today	*or*	☐	$71 in 3 months (90 days)
19	☐	$40 today	*or*	☐	$76 in 3 months (90 days)
20	☐	$40 today	*or*	☐	$81 in 3 months (90 days)
21	☐	$40 in 1 month (30 days)	*or*	☐	$43 in 4 months (120 days)
22	☐	$40 in 1 month (30 days)	*or*	☐	$46 in 4 months (120 days)
23	☐	$40 in 1 month (30 days)	*or*	☐	$48 in 4 months (120 days)
24	☐	$40 in 1 month (30 days)	*or*	☐	$51 in 4 months (120 days)
25	☐	$40 in 1 month (30 days)	*or*	☐	$56 in 4 months (120 days)
26	☐	$40 in 1 month (30 days)	*or*	☐	$61 in 4 months (120 days)
27	☐	$40 in 1 month (30 days)	*or*	☐	$66 in 4 months (120 days)
28	☐	$40 in 1 month (30 days)	*or*	☐	$71 in 4 months (120 days)
29	☐	$40 in 1 month (30 days)	*or*	☐	$76 in 4 months (120 days)
30	☐	$40 in 1 month (30 days)	*or*	☐	$81 in 4 months (120 days)

APPENDIX N Outcome-Expectancies Scale

What do you think the personal consequences for yourself would be if you lose weight?

	1 (Very uncertain)	2	3	4	5	6	7	8 (Very certain)
I would feel physically more attractive. (1)	❍	❍	❍	❍	❍	❍	❍	❍
I will feel better mentally. (2)	❍	❍	❍	❍	❍	❍	❍	❍
I would have no (or fewer body weight problems). (3)	❍	❍	❍	❍	❍	❍	❍	❍

APPENDIX O Dissatisfaction Scale

To what extent do you feel dissatisfied with your current weight loss progress?

APPENDIX P Economics of Obesity

PART I:

We are now going to ask you some questions about your income. This is an important part of this study, so we appreciate your cooperation and accuracy. Remember that everything you tell us will remain confidential and NO information that would identify you will be used in any data analysis.

Do you currently receive income from wages or salary?

1) YES

2) NO

If No, then skip to Part IV. If yes, then continue to PART II.

PART II

[Please show the next three questions simultaneously on the screen, so the respondent sees that they can choose to report their pay by week, fortnight, month, or year and either before or after tax.]

What was the amount of your most recent pay? It will help to answer this question if you can refer to your last pay-slip. Please enter amount in dollars.

And is that before tax or after tax is taken out?

1) Before tax is taken out
2) After tax

And what period does that cover?

1) Week
2) Fortnight
3) Month
4) Year

Continue to Part III.

PART III

Including any paid or unpaid overtime, how many hours PER WEEK do you usually work in all your jobs?

(Please enter the actual number of hours you work per week)

In the past month, how frequently have you had trouble performing the duties of your work because of ill health or disability?

(1) Often; (2) Sometimes; (3) Rarely; (4) Never.

In the past month, how many days did you miss work because of physical illness or injury, or a mental or emotional problem such as stress or depression?

(Please enter actual number of days missed)

In the past month, how frequently have you been discriminated against in the workplace (for example, not hired for a job, not given a promotion or opportunity, fired, or paid less) because of your weight?

(1) Often; (2) Sometimes; (3) Rarely; (4) Never.

How frequently in the past month have you been treated with less courtesy or respect at work because of your weight?

(1) Often; (2) Sometimes; (3) Rarely; (4) Never.

End survey.

PART IV:

In the past month, have you been looking for paid work?

1) Yes; 2) No.

If answered Yes, then ask the next two questions; otherwise (if answered No) then end the survey.

In the past month, how frequently have you had trouble looking for or getting paid work because of ill health or disability?

(1) Often; (2) Sometimes; (3) Rarely; (4) Never;

In the past month, how frequently have you been discriminated against in the labor market (for example, not hired for a job or offered less pay) because of your weight?

(1) Often; (2) Sometimes; (3) Rarely; (4) Never;

End survey.

APPENDIX Q Participant Food Diary

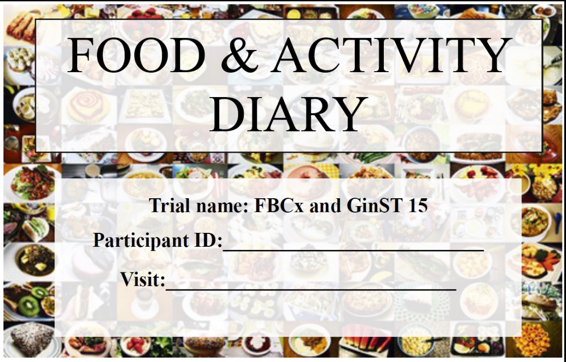

© Alice Gibson, Accredited Practicing Dietitian. Adapted by Mackenzie Fong, Accredited Practicing Dietitian
The Boden Institute

PLEASE READ THROUGH THE FOLLOWING PAGES BEFORE BEGINNING YOUR FOOD DIARY

Please keep a record of everything you eat and drink for 3 days (2 working days and 1 weekend day) prior to your appointment. Please include **ALL** foods and drinks consumed at home and outside the home e.g. at work, friend's houses, restaurants etc. Remember that it is normal to sometimes eat or drink things that you may prefer nobody else knows about. However, for our study we need the most accurate records as possible, so it is very important that you do not avoid writing something down because you think it may be a less desirable option or change what you eat and drink because you are keeping a record. We appreciate that at times completing this diary accurately will take you some time and you may find the process tiresome. We wish to take this opportunity to sincerely **thank you** for your contribution to our research.

2

HOW TO RECORD YOUR FOOD INTAKE

1. Please write clearly and legibly, use a blue or black ball point pen.
2. Start each day on a **new** page and write the **date** at the top.
3. Each time you eat or drink something, record the **time** and **place**. It is best to record at the time of eating or drinking **NOT** from memory at the end of the day. The diary was designed to be a convenient size for a handbag or pocket, so please carry it with you. If you forget to take it with you, try writing on another piece of paper at the time of eating rather than trying to remember later on.

3

HOW TO RECORD YOUR FOOD INTAKE CONTINUED

Describe the food or drink in as much **detail** as possible. Be very **specific** and include **brand names** if possible, **package claims** (e.g. 98% fat free), and the **cooking method** (e.g. boiled, fried, grilled) For example:

Instead of...	Write specifics such as....
Cereal	Weetbix, All Bran, Rice Bubbles
Milk	Skim, lite, or whole milk
Bread	Tip Top white, Helgas wholemeal
Chicken	Grilled, skinless chicken breast, fat trimmed
Sushi	Salmon, avocado, mayonnaise sushi
Muesli bar	Weight watches, almond & apricot muesli bar

4

5. Estimate the amount of food or drink you consumed using:
   1. **Numbers**: e.g. 2 Weetbix
   2. **Weight/volume**: in grams (g) or millilitres (mLs)
   3. **Household measures:** e.g. spoons, cups
   4. **Hands:** fists, finger tips and thumbs
   5. **Dimensions:** measure width, height and length
   6. **Takeaway container size and shape:** small, medium, large

   The instructions on pages 8-14 will show you how!

6. For **recipes**: list **all** the ingredients and indicate **how much** of the recipe you ate (see example on page 6 and 7). Tip: if you have the same recipe or meal again (e.g. as leftovers for lunch), you can just write the name and how much you had.
7. At the end of each day go through the **checklist** of **commonly forgotten foods** on page 15. If you have missed something, go back and write them into your diary. For this reason it is a good idea to leave 1-2 lines between each eating

5

EXAMPLE DAY **DATE:** *Wednesday 1/1/2014*

Time & place	Food description & amount
7.30am,home	*2 weetbix*<br>*1 cup whole milk*<br>*½ large banana*<br>*1 mug of instant coffee with 2 tsp sugar and 2 tbsp whole milk*
11am, café	*1 small skim latte*
11:30am, work	*1 slice chocolate, store bought mud cake (1/8 plate)*
1.30pm, work	*Med ☐ T/A, 1/2 fried rice, 1/2 beef black bean stir fry*<br>*375ml can Pepsi Max*

EXAMPLE

6

3.00pm, work	1 Yoplait 98% fat free berry yoghurt (100 g)
5.30pm, home	Plain Sakata rice crackers (1/2 100 g packet)<br>2 heaped tbs black swan hummus
7.30pm, home	Spaghetti bolognaise (serves 4)<br>520 g lean beef mince<br>1 tbs olive oil<br>1 brown onion<br>2 garlic cloves<br>1 can chopped tin tomatoes<br>1 500 g packet of spaghetti<br>} I ate 1 serving of this recipe
8.30pm, home	6 squares Cadbury milk chocolate

Comments: cake at work for colleague's birthday morning tea (not usual)

EXAMPLE

7

ESTIMATING PORTIONS SIZES

1. Numbers

Example:

For….

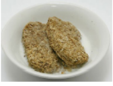

You would write….

2 Weetbix

2. Weight/volume

If you wish to weigh or measure amounts of foods and drinks you consume you may do so, but you are not requested to. A simple way to record the weights or volumes of foods or beverages consumed is to make a note of the weights or volumes from packages and containers.

Example:

For….

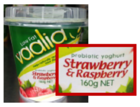

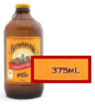

You would write…. *Low fat strawberry and raspberry Vaalia yoghurt 160 g* *Bundaberg ginger beer 375 mL*

8

3. Household measures - Spoons

To estimate food intake using household measures, you can use **measuring spoons** or you can estimate using **regular teaspoons and tablespoons.** Be sure to indicate whether the spoon was level or heaped. Note that 1 teaspoon holds 5 mL and 1 tablespoon holds 20 mL.

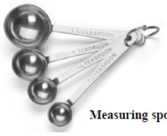

OR

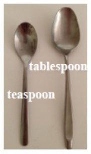

Example:

For...

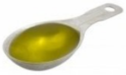

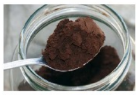

You would write…. *1 level tbsp olive oil* *1 heaped tsp cocoa*

9

3. Household measures continued - cups

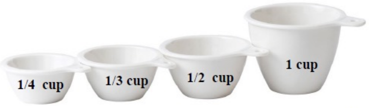

You can **measure or estimate** your portions using **household measuring cups** if desired. Note that 1 cup holds 250 mL.

Example:

For….

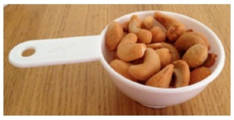

You would write… *1/4 cup of unsalted cashews*

10

4. Hands - fists, fingertips and thumbs

Hands are a very useful and easy way to estimate portions and can be used in different ways to estimate different types of foods.

Fists provide a useful measure of foods that come in clumps or mounds - such as rice, mashed potato, pasta and cous cous. You can record in part fists as well, such as 1 1/2 or 1/2 a fist.

Fingertips and **thumbs** can also be used. Fingertips are measured from the tip to the first knuckle and thumbs to where the thumb joins the hand. Fingertips and thumbs are useful when knives are used - for example for spreads, such as margarine or peanut butter.

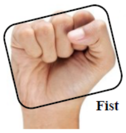

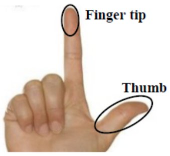

Example:

For….

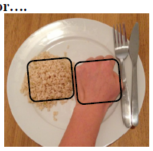

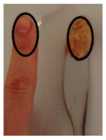

You would write…

brown rice (1 fist size) *1 fingertip of peanut butter*

11

5. Dimensions

Dimensions can be used to estimate the **size** of a portion. This approach works well for food such as meat, chicken fish or tofu and foods that come in slices such as lasagna or pie. It is also useful when you are unsure of the recipe. Measure the **length**, **width and height (thickness)** using a ruler. **A ruler has been provided for you on page 34-35 of this booklet.** Measure the thickness of a food at it's thickest point.

Example:

For….

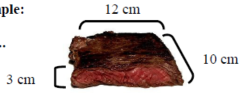

You would write…

Beef steak (fat trimmed)
3 cm x 12 cm x 10 cm

For….

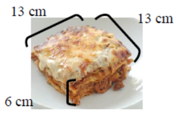

You would write…

Beef and tomato lasagna with white sauce and cheese
6 cm x 13 cm x 13 cm

12

6. Takeaway container size and shape

For food that comes in takeaway containers you can estimate your portion size by indicating the type of container (rectangular or circular) or and size (small, medium or large).

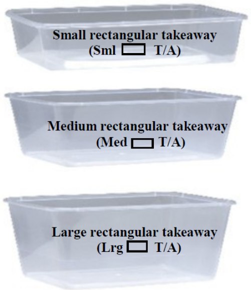

Example:

For….

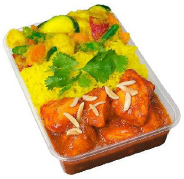

You would write…

Sml ▭ T/A w/ 1/3 vegetable curry, 1/3 yellow rice and 1/3 butter chicken.

13

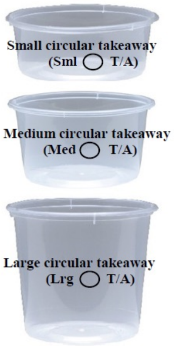

For….

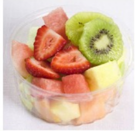

You would write…

Med ◯ T/A fruit salad (kiwi, strawberry, watermelon, rockmelon)

16

CHECKLIST OF COMMONLY FORGOTTEN FOODS

Read through the list below each day and add anything you may have forgotten to your food diary

- Salad dressing
- Sauces
- Oils
- Spreads
- Condiments
- Coffee
- Tea
- Milk in tea/coffee
- Sugar in tea/coffee
- Soft drink
- Juice
- Water
- Beer
- Wine
- Other alcoholic drinks
- Biscuits
- Lollies
- Chocolate
- Ice-cream
- Chips
- Crackers
- Nuts
- Fruits
- Vegetables
- Cheese
- Breads (rolls/wraps)
- Other snack foods

17

PLEASE READ THE FOLLOWING PAGES BEFORE BEGINNING YOUR ACTIVITY DIARY

Please record your activity for **5 days** prior to your appointment. You will be provided with an accelerometer which will count the number of steps you walk. Wear the device from the moment you **wake up** to the moment you **go to bed**. Once you have taken it off at night, record your step count in the diary on page 31 and **reset** it using the 'Reset' button on the bottom right hand corner. Please note the device is **not water proof** and should not be worn in the shower or while swimming.

Example:

Date	Number of steps
1/1/2014	4500
2/1/2014	7000
3/1/2014	10000

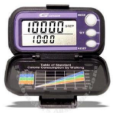

18

HOW TO WEAR THE ACTIVITY MONITOR

It is important that you wear the activity monitor on your waist band., with the monitor positioned on your hip bone (see image below). Ensure that the activity monitor **fits you snugly** as shown in the picture below. It will not record your activities accurately if it is loose. You can wear the activity monitor underneath or on top of your clothes.

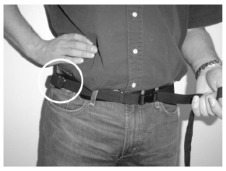

19

DAY 1 Food DATE: ______________________

Time & place	Food description & amount

20

Comments:

21

DAY 1 Food (continued from previous page)

Time & place	Food description & amount
	Have you forgotten anything? Use the checklist on page x to help.

Comments:

22

DAY 2 Food DATE: ______________________

Time & place	Food description & amount

23

Comments:

24

DAY 2 Food (continued from previous page)

Time & place	Food description & amount
	Have you forgotten anything? Use the checklist on page x to help.

Comments:

25

DAY 3 Food DATE:____________________

Time & place	Food description & amount

26

Comments:

27

DAY 3 Food (continued from previous page)

Time & place	Food description & amount (include lots of detail)
	Have you forgotten anything? Use the checklist on page x to help.

Comments:

28

ACTIVITY DIARY

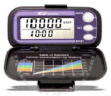

Record your activity for 5 days prior to your appointment. Please wear your accelerometer all day from when you wake up until when you go to bed (but not in the water). Record your step count each night in the table below, and reset the device for the next day.

Date	Number of steps

29

Ruler

Use this ruler to measure the dimensions of your food. Measure the **length, width and height (thickness).** See page 12 for more information on how to record dimensions.

1 2 3 4 5 6 7 8 9 10 11 12

30

13 14 15 16 17 18 19 20 21 22 23 24

31

This booklet is extremely valuable to us because it contains irreplaceable research data.

If found, please return to:
Mackenzie Fong: The Boden Institute, Charles Perkins Centre D17, The University of Sydney, NSW 2006
Or call:
(02) 8627 1931

© Alice Gibson, Accredited Practicing Dietitian. Adapted by Mackenzie Fong, Accredited Practicing Dietitian The Boden Institute, the University of Sydney

APPENDIX R Australian Type 2 Diabetes Risk Assessment Tool

The Australian Type 2 Diabetes Risk Assessment Tool (AUSDRISK) ID:

Name: My GP is:

Date of Birth: / / Date completed: / /

1. Your age group?

Under 35 years	0 points
35 - 44 years	2 points
45 - 54 years	4 points
55 - 64 years	6 points
65 years or over	8 points

2. Your gender?

Female	0 points
Male	3 points

3. Your Ethnicity/Country of birth:

3a. Are you of Aboriginal, Torres Strait Islander, Pacific Islander or Maori descent?

No	0 points
Yes	2 points

3b.Where were you born?

Asia (including the Indian subcontinent), Middle East, North Africa, Southern Europe	2 points
Other	0 points

4. Have either of your parents, or any of your brothers or sisters been diagnosed with diabetes (type 1 or type 2)?

No	0 points
Yes	3 points

5. Have you ever been found to have high blood glucose (sugar) (for example, in a health examination, during an illness, during pregnancy)?

No	0 points
Yes	6 points

6. Are you currently taking medication for high blood pressure?

No	0 points
Yes	2 points

7. Do you currently smoke cigarettes or any other tobacco products on a daily basis?

No	0 points
Yes	2 points

8. How often do you eat vegetables or fruit?

Every day	0 points
Not every day	1 point

9. On average, would you say you do at least 2.5 hours of physical activity per week (for example, 30 minutes a day on 5 or more days a week)?

Yes	0 points
No	2 points

10. Your waist measurement taken below the ribs (usually at the level of the navel)?

cm

For those of Asian or Aboriginal or Torres Strait Islander descent:

Men	Women	
Less than 90 cm	Less than 80 cm	0 Points
90 - 100 cm	80 – 90 cm	4 points
More than100 cm	More than 90 cm	7 points

For all others:

Men	Women	
Less than 102 cm	Less than 88 cm	0 points
102 – 110 cm	88 – 100 cm	4 points
More than 110 cm	More than100 cm	7 points

Add up your score

Your risk of developing type 2 diabetes within 5 years*:

5 or less: Low risk
Approximately one person in every 100 will develop diabetes.

6-14: Intermediate risk
For scores of 6-8, approximately one person in every 50 will develop diabetes.

For scores of 9-14, approximately one person in every 20 will develop diabetes.

15 or more: High risk
For scores of 15-19, approximately one person in every 7 will develop diabetes.

For scores of 20 and above, approximately one person in every 3 will develop diabetes.

If you scored 15 or more points, it is important that you discuss your score with your doctor.

*The overall score may overestimate the risk of diabetes in those aged less than 25 years and underestimate the risk of diabetes in people of Aboriginal and Torres Strait Islander descent.

The Australian Type 2 Diabetes Risk Assessment Test was originally developed by the International Diabetes Institute on behalf of the Australian, State and Territory Governments as part of the COAG *reducing the risk of type 2 diabetes* initiatives

Information for GPs

Risk Score is the risk of developing type 2 diabetes within the next 5 years.

Risk Score <15

1-5: Low Risk
Approx 1 in 100 develops diabetes
6-14: Intermediate Risk
6-8: Approx 1 in 50 develops diabetes
9-14: Approx 1 in 20 develops diabetes

If Risk Score is 1- 5:

Congratulate patient on low risk status.
Encourage patient to remain low risk through:

- Maintaining a healthy weight (BMI <25).
- Eating a healthy diet.
- Being physically active (at least 30 minutes of physical activity every day).

If Risk Score is 6-14:

- Further testing may be required.
- Provide appropriate advice regarding diet and physical activity as above.
- Suggest regular GP visits to check for diabetes risk.

Diabetes identified – refer to usual care

Risk Score ≥15

≥15: High Risk
15-19: Approx 1 in 7 develops diabetes
≥20: Approx 1 in 3 develops diabetes

Please consider you patient for the *Live Life Well* Diabetes Prevention Program.

Organise FPG test (to exclude diabetes) and full lipid profile (total cholesterol, HDL, LDL triglycerides).

Note: If diabetes has been excluded by a FPG test within the last 3 months, refer patient to *Live Life Well* Diabetes Prevention Program.

Encourage patient to make an appointment to discuss FPG test results.

Diabetes excluded – complete *Live Life Well* Diabetes Prevention Program referral form.

Remember your patient is ineligible if they:

- Are taking or have taken Metformin or other hypoglycaemic medication within the past month
- Are taking prescribed weight loss medication
- Have any of the following conditions:
  - Type 1 or 2 diabetes (offer routine diabetes care and referrals)
  - Pregnancy
  - End stage congestive heart failure
  - Severe cognitive impairment or behavioural disturbances
  - Untreated sever aortic stenosis or other structural heart disease
  - Progressive or terminal cancer
  - Unstable abdominal, thoracic or cerebral aneurysm
  - Malignant arrhythmias
  - Unstable CAD

APPENDIX S

A double blinded, randomised controlled trial to determine the efficacy of FBCx (a formula based on α-cyclodextrin) on cholesterol control, and the efficacy of Ginst15 (a ginseng extract formula based on Compound K) on glycaemic control.

Oral Examination Sub-study

DENTAL DATA COLLECTION SHEET

PARTICIPANT

Name: ______________________ **Group:**_________

Age: _________ **Gender:**_________ **Postcode:**_________

DMFT/dmft

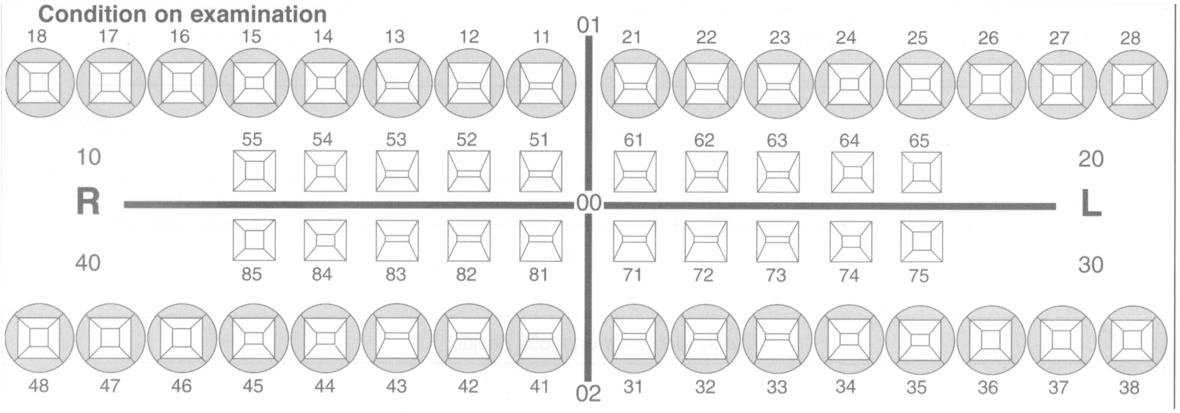

APPENDIX T

A double blinded, randomised controlled trial to determine the efficacy of FBCx (a formula based on α-cyclodextrin) on cholesterol control, and the efficacy of Ginst15 (a ginseng extract formula based on Compound K) on glycaemic control.

Oral Examination Sub-study

Questions adopted from: NATIONAL SURVEY OF ADULT ORAL HEALTH 2004 – 2005

ARCPOH
Australian Research Centre for POPULATION ORAL HEALTH

How to answer

Most items are answered by ticking one box that best describes your answer *(Example 1).*

EXAMPLE 1

	Strongly disagree	Disagree	Neutral	Agree	Strongly agree
I was satisfied with the dental care I received.	☐ 1	☑ 2	☐ 3	☐ 4	☐ 5

Others are answered by writing a number in the box *(Example 2).*

EXAMPLE 2

How many glasses of plain water did you drink yesterday?	6<br>Glasses	*Write '0' if you did not drink plain water yesterday*

The following questions ask about dental health behaviours.

A1. In the last week, how many times did you **brush** your **teeth**?	Brush	*Write '0' if you did not brush your teeth.*
A2. In the last week, how many times did you use a **mouth rinse** or **mouth wash**?	Mouthrinse	*Write '0' if you did not use mouth rinse.*
A3. In the last week, how many times did you chew **sugar-free gum** for at least 10 minutes continuously?	Sugar-free gum	*Write '0' if you did not chew sugar-free gum.*

A4. Please indicate whether you regularly use either dental floss, dental tape, or an interdental brush.<br>*(If you use more than one, please tick the **one** most often used.)*	**No, I use none of these** *(Go to page 3)*	Yes, dental floss	Yes, dental tape	Yes, interdental brush

A5. In the last week, how many times did you clean **between** your **teeth** (using dental floss, dental tape or an interdental brush)?	Clean between teeth	*Write '0' if you did not clean between your teeth.*

The questions below ask about troubles that people may have in daily life because of dental problems. Oral Health Impact Profile

HOW OFTEN during the last year	*Please tick ONE box that best describes your experience*				
	Very Often	Fairly Often	Occasionall y	Hardly Ever	Never
B1… have you had trouble **pronouncing any words** because of problems with your teeth, mouth or dentures?	☐1	☐2	☐3	☐4	☐5
B2… have you felt that your **sense of taste** has worsened because of problems with your teeth, mouth or dentures?	☐1	☐2	☐3	☐4	☐5
B3… have you had **painful aching** in your mouth?	☐1	☐2	☐3	☐4	☐5
B4… have you found it **uncomfortable to eat any foods** because of problems with your teeth, mouth or dentures?	☐1	☐2	☐3	☐4	☐5
B5… have you been **self conscious** because of problems with your teeth, mouth or dentures?	☐1	☐2	☐3	☐4	☐5

	Very Often	Fairly Often	Occasionally	Hardly Ever	Never
B6… have you **felt tense** because of problems with your teeth, mouth or dentures?	☐1	☐2	☐3	☐4	☐5

	Very Often	Fairly Often	Occasionall y	Hardly Ever	Never
B7… has your **diet been unsatisfactory** because of problems with your teeth, mouth or dentures?	☐1	☐2	☐3	☐4	☐5

	Very Often	Fairly Often	Occasionally	Hardly Ever	Never
B8… have you had to **interrupt meals** because of problems with your teeth, mouth or dentures?	☐1	☐2	☐3	☐4	☐5

	Very Often	Fairly Often	Occasionall y	Hardly Ever	Never
B9… have you found it **difficult to relax** because of problems with your teeth, mouth or dentures?	☐1	☐2	☐3	☐4	☐5

	Very Often	Fairly Often	Occasionall y	Hardly Ever	Never
B10… have you been a bit **embarrassed** because of problems with your teeth, mouth or dentures?	☐1	☐2	☐3	☐4	☐5

	Very Often	Fairly Often	Occasionall y	Hardly Ever	Never
B11… have you been a bit **irritable with other people** because of problems with your teeth, mouth or dentures?	☐1	☐2	☐3	☐4	☐5

	Very Often	Fairly Often	Occasionall y	Hardly Ever	Never
B12… have you had **difficulty doing your usual jobs** because of problems with your teeth, mouth or dentures?	☐1	☐2	☐3	☐4	☐5

	Very Often	Fairly Often	Occasionall y	Hardly Ever	Never
B13… have you felt that life in general was **less satisfying** because of problems with your teeth, mouth or dentures?	☐1	☐2	☐3	☐4	☐5

	Very Often	Fairly Often	Occasionall y	Hardly Ever	Never
B14… have you been totally **unable to function** because of problems with your teeth, mouth or dentures?	☐1	☐2	☐3	☐4	☐5

Dental visiting behaviour question

**HOW OFTEN during the last year**	*Please tick ONE box that best describes your experience*			
C1. Which is your usual reason for visiting a dental professional, for check-ups or when you have a dental problem?	Check-up	Problem	Don't know/refused	

Thank you for completing this questionnaire.
