# Supplementary material for: Effects of α-Cyclodextrin on Cholesterol Control and Hydrolyzed Ginseng Extract on Glycemic Control in People With Prediabetes: A Randomized Clinical Trial
Source: JAMA Netw Open. 2020 Nov 17;3(11):e2023491. doi: 10.1001/jamanetworkopen.2020.23491 (PMC7672512; doi:10.1001/jamanetworkopen.2020.23491)
Supplement: Supplement 2. — eTable 1. Comparison of Outcomes at Six Months Between Participants Taking Alpha-Cyclodextrin (n = 159) and Participants Taking Placebo (n = 174) in the Completers Analysis eTable 2. Comparison of Outcomes at Six Months Between Participants Taking Alpha-Cyclodextrin (n = 86) and Participants Taking Placebo (n = 90) in the Subsample of Participants Who Were Compliant With the Dosing Regimen eTable 3. Comparison of Outcomes at Six Months Between Participants Taking Alpha-Cyclodextrin (n = 109) and Participants Taking Placebo (n = 119) in the Subsample of Participants Who Had High Cholesterol at Baseline eTable 4. Comparison of Outcomes at Six Months Between Participants Taking Hydrolysed Ginseng Extract (n = 171) and Participants Taking Placebo (n = 162) in the Completers Analysis eTable 5. Comparison of Outcomes at Six Months Between Participants Taking Hydrolysed Ginseng Extract (n = 99) and Participants Taking Placebo (n = 96) in the Subsample of Participants Who Were Compliant With the Dosing Regimen eTable 6. Comparison of Outcomes at Six Months Between Participants Taking Hydrolysed Ginseng Extract (n = 109) and Participants Taking Placebo (n = 117) in the Subsample of Participants Who Met the Guidelines for Prediabetes at Baseline eTable 7. Comparison of Outcomes at Six Months Between Participants Taking Hydrolysed Ginseng Extract (n = 79) and Participants Taking Placebo (n = 85) in the Subsample of Participants Who Were Compliant With the Dosing Regimen for Both Supplements eTable 8. Number of Participants Reporting Adverse Events Rated as “Possibly” or “Probably” Related to the Investigational Products Between Baseline and Six Months [file jamanetwopen-e2023491-s002.pdf]

Supplemental Online Content

Bessell E, Fuller NR, Markovic TP, et al. Effects of α-cyclodextrin on cholesterol control and hydrolyzed ginseng extract on glycemic control in people with prediabetes: a randomized clinical trial. *JAMA Netw Open*. 2020;3(11):e2023491. doi:10.1001/jamanetworkopen.2020.23491

eTable 1. Comparison of Outcomes at Six Months Between Participants Taking Alpha-Cyclodextrin (n = 159) and Participants Taking Placebo (n = 174) in the Completers Analysis

eTable 2. Comparison of Outcomes at Six Months Between Participants Taking Alpha-Cyclodextrin (n = 86) and Participants Taking Placebo (n = 90) in the Subsample of Participants Who Were Compliant With the Dosing Regimen

eTable 3. Comparison of Outcomes at Six Months Between Participants Taking Alpha-Cyclodextrin (n = 109) and Participants Taking Placebo (n = 119) in the Subsample of Participants Who Had High Cholesterol at Baseline

eTable 4. Comparison of Outcomes at Six Months Between Participants Taking Hydrolysed Ginseng Extract (n = 171) and Participants Taking Placebo (n = 162) in the Completers Analysis

eTable 5. Comparison of Outcomes at Six Months Between Participants Taking Hydrolysed Ginseng Extract (n = 99) and Participants Taking Placebo (n = 96) in the Subsample of Participants Who Were Compliant With the Dosing Regimen

eTable 6. Comparison of Outcomes at Six Months Between Participants Taking Hydrolysed Ginseng Extract (n = 109) and Participants Taking Placebo (n = 117) in the Subsample of Participants Who Met the Guidelines for Prediabetes at Baseline

eTable 7. Comparison of Outcomes at Six Months Between Participants Taking Hydrolysed Ginseng Extract (n = 79) and Participants Taking Placebo (n = 85) in the Subsample of Participants Who Were Compliant With the Dosing Regimen for Both Supplements

eTable 8. Number of Participants Reporting Adverse Events Rated as “Possibly” or “Probably” Related to the Investigational Products Between Baseline and Six Months

This supplemental material has been provided by the authors to give readers additional information about their work.

© 2020 Bessell E et al. *JAMA Network Open.*

eTable 1. Comparison of Outcomes at Six Months Between Participants Taking Alpha-Cyclodextrin (n = 159) and Participants Taking Placebo (n = 174) in the Completers Analysis

	αCD (n=159)		Placebo (n=174)			
**Characteristic [a]**	**Baseline**	**Month 6**	**Baseline**	**Month 6**	**Adjusted Difference (95%CI) [b]**	**P-value**
Weight (kg)	95.5 (19.3)	92.6 (19.8)	98.8 (20.2)	95.2 (19.8)	+0.78 (-0.15, 1.70)	0.099
BMI (kg/m^2)	33.8 (5.9)	32.7 (6.1)	34.8 (6.5)	33.6 (6.5)	+0.23 (-0.10, 0.56)	0.166
Weight loss (%)	..	3.1 (4.0)	..	3.7 (4.2)	-0.63 (-1.53, 0.26)	0.162
Fasting glucose (mg/dL)	99 (11)	96 (13)	99 (9)	96 (11)	+0.9 (-1.1, 2.9)	0.371
HbA1c (%)	5.6 (0.4)	5.5 (0.4)	5.6 (0.3)	5.5 (0.3)	+0.02 (-0.02, 0.07)	0.342
Total cholesterol (mg/dL)	216 (39)	212 (39)	224 (39)	220 (39)	-3.5 (-8.9, 2.3)	0.230
LDL cholesterol (mg/dL)	139 (35)	135 (31)	143 (35)	143 (35)	-5.0 (-9.7, 0.0)	0.052
HDL cholesterol (mg/dL)	54 (12)	54 (12)	54 (12)	54 (12)	+0.0 (-1.2, 1.5)	0.815
Triglycerides (mg/dL)	124 (62)	133 (89)	142 (80)	133 (71)	+6.2 (-8.0, 19.5)	0.385

[a] Values are means (SD) unless otherwise indicated. [b] Adjusted for baseline observation.

SI conversion factors: To convert fasting glucose to mmol/L, multiply by 0.0555; total, LDL and HDL cholesterol to mmol/L, multiply by 0.0259; and triglycerides to mmol/L, multiple by 0.0113.

αCD – alpha-cyclodextrin. BMI – body mass index. HDL – high-density lipoprotein. LDL – Low-density lipoprotein. SD – standard deviation.

© 2020 Bessell E et al. *JAMA Network Open.*

eTable 2. Comparison of Outcomes at Six Months Between Participants Taking Alpha-Cyclodextrin (n = 86) and Participants Taking Placebo (n = 90) in the Subsample of Participants Who Were Compliant With the Dosing Regimen

Characteristic [a]	αCD (n=86)	Placebo (n=90)	Adjusted Difference (95%CI) [b]	P-value
Weight (kg)	88.0 (18.3)	92.5 (19.2)	+0.79 (-0.56, 2.14)	0.251
Weight loss (%)	4.3 (4.2)	5.2 (4.7)	-0.87 (-2.20, 0.46)	0.201
Total cholesterol (mg/dL)	216 (39)	220 (35)	+1.2 (-7.0, 9.3)	0.801
LDL cholesterol (mg/dL)	135 (35)	139 (31)	-0.4 (-7.3, 6.6)	0.924
HDL cholesterol (mg/dL)	54 (12)	54 (12)	-0.8 (-2.7, 1.5)	0.570
Triglycerides (mg/dL)	133 (97)	133 (80)	+15.0 (-8.0, 38.1)	0.190

[a] Values are means (SD) unless otherwise indicated. [b] Adjusted for baseline observation.

SI conversion factors: To convert fasting glucose to mmol/L, multiply by 0.0555; total, LDL and HDL cholesterol to mmol/L, multiply by 0.0259; and triglycerides to mmol/L, multiple by 0.0113.

αCD – alpha-cyclodextrin. BMI – body mass index. HDL – high-density lipoprotein. LDL – Low-density lipoprotein. SD – standard deviation.

© 2020 Bessell E et al. *JAMA Network Open.*

eTable 3. Comparison of Outcomes at Six Months Between Participants Taking Alpha-Cyclodextrin (n = 109) and Participants Taking Placebo (n = 119) in the Subsample of Participants Who Had High Cholesterol at Baseline

Characteristic [a]	αCD (n=109)	Placebo (n=119)	Adjusted Difference (95%CI) [b]	P-value
Total cholesterol (mg/dL)	236 (27)	236 (35)	+0.8 (-6.6, 8.1)	0.819
LDL cholesterol (mg/dL)	151 (27)	154 (31)	-1.5 (-8.1, 4.6)	0.607

[a] Values are means (SD) unless otherwise indicated. [b] Adjusted for baseline observation.

SI conversion factors: To convert fasting glucose to mmol/L, multiply by 0.0555; total, LDL and HDL cholesterol to mmol/L, multiply by 0.0259; and triglycerides to mmol/L, multiple by 0.0113.

αCD – alpha-cyclodextrin. BMI – body mass index. HDL – high-density lipoprotein. LDL – Low-density lipoprotein. SD – standard deviation.

© 2020 Bessell E et al. *JAMA Network Open.*

eTable 4. Comparison of Outcomes at Six Months Between Participants Taking Hydrolysed Ginseng Extract (n = 171) and Participants Taking Placebo (n = 162) in the Completers Analysis

	HGE (n=171)		Placebo (n=162)			
**Characteristic [a]**	**Baseline**	**Month 6**	**Baseline**	**Month 6**	**Adjusted Difference (95%CI) [b]**	**P-value**
Weight (kg)	97.6 (20.5)	94.0 (20.3)	96.9 (19.2)	93.9 (19.3)	-0.54 (-1.46, 0.38)	0.252
BMI (kg/m^2)	34.4 (6.6)	33.1 (6.6)	34.3 (5.9)	33.2 (6.1)	-0.24 (-0.57, 0.08)	0.145
Weight loss (%)	..	3.6 (4.3)	..	3.2 (4.0)	+0.51 (-0.38, 1.40)	0.259
Fasting glucose (mg/dL)	97 (9)	96 (13)	99 (11)	96 (11)	+0.2 (-1.8, 2.2)	0.863
HbA1c (%)	5.6 (0.3)	5.5 (0.3)	5.6 (0.4)	5.6 (0.4)	-0.02 (-0.07, 0.02)	0.357
Total cholesterol (mg/dL)	220 (39)	220 (39)	220 (39)	216 (39)	+1.5 (-4.3, 7.0)	0.600
LDL cholesterol (mg/dL)	139 (35)	139 (35)	139 (31)	139 (31)	+0.8 (-4.3, 5.8)	0.731
HDL cholesterol (mg/dL)	54 (12)	54 (12)	54 (12)	54 (12)	-0.0 (-1.5, 1.2)	0.819
Triglycerides (mg/dL)	133 (62)	133 (80)	133 (71)	133 (71)	+3.5 (-9.7, 17.7)	0.581

[a] Values are means (SD) unless otherwise indicated. [b] Adjusted for baseline observation.

SI conversion factors: To convert fasting glucose to mmol/L, multiply by 0.0555; total, LDL and HDL cholesterol to mmol/L, multiply by 0.0259; and triglycerides to mmol/L, multiple by 0.0113.

BMI – body mass index. HDL – high-density lipoprotein. HGE – hydrolysed ginseng extract. LDL – Low-density lipoprotein. SD – standard deviation.

© 2020 Bessell E et al. *JAMA Network Open.*

eTable 5. Comparison of Outcomes at Six Months Between Participants Taking Hydrolysed Ginseng Extract (n = 99) and Participants Taking Placebo (n = 96) in the Subsample of Participants Who Were Compliant With the Dosing Regimen

Characteristic [a]	αCD (n=99)	Placebo (n=96)	Adjusted Difference (95%CI) [b]	P-value
Fasting glucose (mg/dL)	96 (11)	96 (9)	+1.1 (-1.3, 3.4)	0.394
HbA1c (%)	5.5 (0.3)	5.5 (0.3)	-0.02 (-0.07, 0.04)	0.528

[a] Values are means (SD) unless otherwise indicated. [b] Adjusted for baseline observation.

SI conversion factors: To convert fasting glucose to mmol/L, multiply by 0.0555; total, LDL and HDL cholesterol to mmol/L, multiply by 0.0259; and triglycerides to mmol/L, multiple by 0.0113.

BMI – body mass index. HDL – high-density lipoprotein. HGE – hydrolysed ginseng extract. LDL – Low-density lipoprotein. SD – standard deviation.

© 2020 Bessell E et al. *JAMA Network Open.*

eTable 6. Comparison of Outcomes at Six Months Between Participants Taking Hydrolysed Ginseng Extract (n = 109) and Participants Taking Placebo (n = 117) in the Subsample of Participants Who Met the Guidelines for Prediabetes at Baseline

Characteristic [a]	HGE (n=109)	Placebo (n=117)	Adjusted Difference (95%CI) [b]	P-value
Fasting glucose (mg/dL)	99 (13)	99 (11)	-0.0 (-2.7, 2.7)	0.994
HbA1c (%)	5.6 (0.3)	5.7 (0.4)	-0.01 (-0.07, 0.05)	0.759

[a] Values are means (SD) unless otherwise indicated. [b] Adjusted for baseline observation.

SI conversion factors: To convert fasting glucose to mmol/L, multiply by 0.0555; total, LDL and HDL cholesterol to mmol/L, multiply by 0.0259; and triglycerides to mmol/L, multiple by 0.0113.

BMI – body mass index. HDL – high-density lipoprotein. HGE – hydrolysed ginseng extract. LDL – Low-density lipoprotein. SD – standard deviation.

© 2020 Bessell E et al. *JAMA Network Open.*

eTable 7. Comparison of Outcomes at Six Months Between Participants Taking Hydrolysed Ginseng Extract (n = 79) and Participants Taking Placebo (n = 85) in the Subsample of Participants Who Were Compliant With the Dosing Regimen for Both Supplements

Characteristic [a]	HGE (n=79)	Placebo (n=85)	Adjusted Difference (95%CI) [b]	P-value
Weight (kg)	91.0 (20.0)	90.8 (18.5)	-1.63 (-3.03, -0.22)	0.023*
BMI (kg/m^2)	32.5 (7.0)	32.0 (5.8)	-0.68 (-1.20, -0.16)	0.010*
Weight loss (%)	5.6 (4.4)	4.0 (4.5)	+1.72 (0.35, 3.10)	0.015*

[a] Values are means (SD) unless otherwise indicated. [b] Adjusted for baseline observation.

SI conversion factors: To convert fasting glucose to mmol/L, multiply by 0.0555; total, LDL and HDL cholesterol to mmol/L, multiply by 0.0259; and triglycerides to mmol/L, multiple by 0.0113.

BMI – body mass index. HDL – high-density lipoprotein. HGE – hydrolysed ginseng extract. LDL – Low-density lipoprotein. SD – standard deviation.

© 2020 Bessell E et al. *JAMA Network Open.*

eTable 8. Number of Participants Reporting Adverse Events Rated as "Possibly" or "Probably" Related to the Investigational Products Between Baseline and Six Months

	HGE + αCD (n = 101)	HGE only (n = 101)	αCD only (n = 99)	Placebo (n = 100)
**Gastrointestinal**				
Abdominal pain	4	9	4	1
Bloating	5	3	2	3
Bowel urgency	1	0	0	2
Constipation	8	3	8	1
Diarrhoea	2	9	6	1
Flatulence	2	2	1	0
Heartburn or reflux	2	3	2	2
Indigestion	2	0	1	2
Loose bowels	3	2	1	3
Nausea	3	3	5	0
**Other**				
Cough	5	0	3	1
Rash or pruritus	7	6	2	0
Throat discomfort	0	0	0	1
Sleeplessness	3	2	0	1

αCD – alpha-cyclodextrin. HGE – hydrolysed ginseng extract.

© 2020 Bessell E et al. *JAMA Network Open.*
