# Supplementary material for: Effects of α-Cyclodextrin on Cholesterol Control and Hydrolyzed Ginseng Extract on Glycemic Control in People With Prediabetes: A Randomized Clinical Trial
Source: JAMA Netw Open. 2020 Nov 17;3(11):e2023491. doi: 10.1001/jamanetworkopen.2020.23491 (PMC7672512; doi:10.1001/jamanetworkopen.2020.23491)
Supplement: Supplement 3. — Data Sharing Statement [file jamanetwopen-e2023491-s003.pdf]

Data Sharing Statement

Bessell. Effects of α-Cyclodextrin on Cholesterol Control and Hydrolyzed Ginseng Extract on Glycemic Control in People With Prediabetes. *JAMA Netw Open*. Published November 17, 2020. 10.1001/jamanetworkopen.2020.23491

Data
Data available: Yes
Data types: Deidentified participant data
How to access data: Send requests to principal investigator Dr Nicholas Fuller (nick.fuller@sydney.edu.au)
When available: With publication

Supporting Documents
Document types: None

Additional Information
Who can access the data: Send requests to principal investigator Dr Nicholas Fuller (nick.fuller@sydney.edu.au)
Types of analyses: Send requests to principal investigator Dr Nicholas Fuller (nick.fuller@sydney.edu.au)
Mechanisms of data availability: Send requests to principal investigator Dr Nicholas Fuller (nick.fuller@sydney.edu.au)
